# A Chiral Saddle‐Shaped Nanographene With Two Heptagon‐Embedded [4]Helicenes

**DOI:** 10.1002/anie.3481279

**Published:** 2026-06-02

**Authors:** Felix Trautner, Boris Borrisov, Philipp Royla, Hongqing Zhao, Hartmut Komber, Li Wan, Evgenia Dmitrieva, Ji Ma, Jan J. Weigand, Qun Yang, Xinliang Feng, Wenhui Niu

**Affiliations:** ^1^ Max Planck Institute of Microstructure Physics Halle Germany; ^2^ Center for Advancing Electronics Dresden (CFAED) & Faculty of Chemistry and Food Chemistry TECHNISCHE Universität Dresden Dresden Germany; ^3^ Chair of Inorganic Molecular Chemistry Faculty of Chemistry and Food Chemistry Technische Universität Dresden Dresden Germany; ^4^ Faculty of Chemistry and Pharmacy Ludwig‐Maximilian‐Universität München Munich Germany; ^5^ Leibniz‐Institut Für Polymerforschung Dresden e. V. Dresden Germany; ^6^ Leibniz Institute for Solid State and Materials Research (IFW) Dresden Dresden Germany; ^7^ College of Materials Science and Optoelectronic Technology & Center of Materials Science and Optoelectronics Engineering University of Chinese Academy of Science Beijing China

**Keywords:** chiral saddle, cocrystal, heptagon, non‐benzenoid nanographene, supramolecular complexation

## Abstract

Chiral non‐benzenoid nanographenes (NGs) remain highly appealing, yet underexplored synthetic targets due to the lack of feasible synthetic strategies to simultaneously construct non‐hexagonal rings and stable chirality. Herein, we demonstrate a novel synthetic strategy to construct a chiral saddle‐shaped nanographene (**cSNG**) via stepwise oxidation of an anthracene‐containing oligophenylene precursor. Scholl‐type oxidation first yields a key intermediate featuring *sp* ^3^‐defect heptagons, and subsequent oxidative dehydrogenation furnishes target **cSNG** incorporating two heptagon‐embedded [4]helicenes. The structure of **cSNG** is unambiguously confirmed by single‐crystal analysis, demonstrating its saddle‐shaped geometry with intrinsic chirality. Remarkably, due to the heptagon‐embedded [4]helicene subunits, **cSNG** exists as configurationally stable enantiomers which enable chiral resolution, leading to pronounced chiroptical responses and enhanced dissymmetry factors, compared with its *sp* ^3^‐defect precursor. Moreover, profiting from chiral saddle geometry with shape‐adaptive character, **cSNG** presents strong host–guest interactions with fullerenes, with association constants up to 2.3 × 10^4^ M^−1^ for C_60_ and 8.7 × 10^4^ M^−1^ for C_70_, which are among the highest values reported for negatively curved nanographenes. Crystallographic analysis of cocrystals of **cSNG** with C_60_ further revealed a distinct 1:3 binding mode for supramolecular complexation, featuring two fullerenes accommodated within the saddle cavity and two additional fullerenes externally shared between neighboring saddle units.

## Graphical abstract

We present a novel synthetic strategy toward a chiral saddle‐shaped nanographene (**cSNG**) via a two‐step oxidation of a prochiral anthracene precursor. The Scholl‐type oxidation first yields a key intermediate with *sp* ^3^‐defect heptagons, and subsequent oxidative dehydrogenation affords fully conjugated **cSNG**. Owing to heptagon‐embedded helicene subunits, **cSNG** displays pronounced chiroptical properties and strong host–guest interactions with fullerenes.

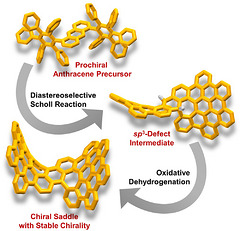

## 1 Introduction

**FIGURE 1:**
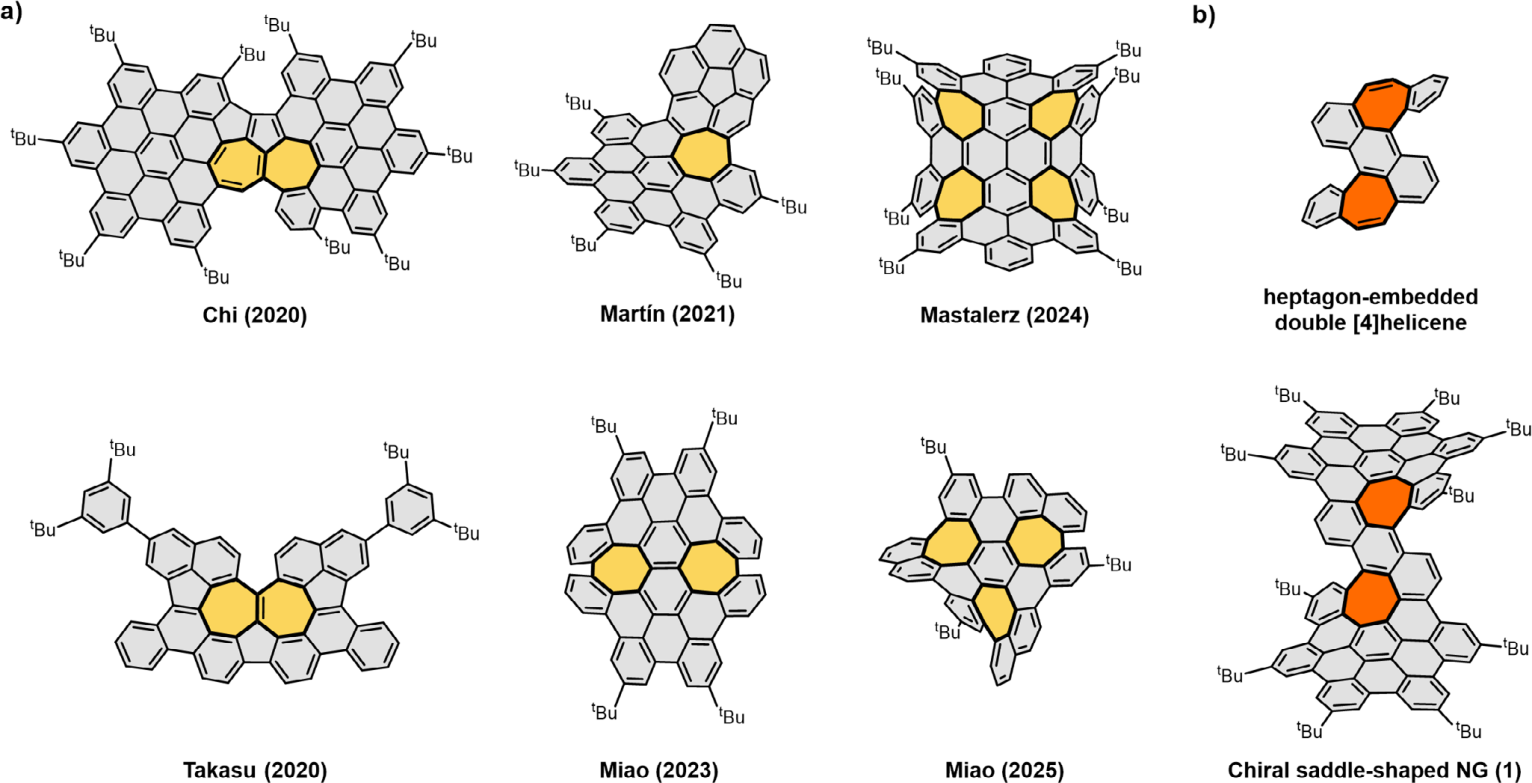
(a) Representative examples of reported NGs containing seven‐membered rings. (b) Chiral saddle‐shaped nanographene (**cSNG**) presented in this work.

Non‐benzenoid nanographenes (NGs) incorporating pentagons or heptagons have gained significant attention over the past decades owing to their unique topologies, aromaticity, and exotic optoelectronic properties, compared to their benzenoid counterparts [1, 2, 3, 4, 5, 6, 7, 8, 9, 10, 11]. In contrast to pentagonal‐ring‐embedded NGs, which display positive curvature and bowl‐shaped structures, the incorporation of heptagonal rings induces negative curvature and unique saddle‐shaped geometries, providing impetus to the development of elusive carbon allotropes, such as carbon Schwarzites and Mackay crystals [12]. Such negatively curved systems exhibit intriguing 3D architectures and interesting aromatic characteristics [4, 13, 14, 15]. For the synthesis of heptagon‐embedded NGs, the formation of precursors containing corannulene, naphthalene, or triphenylene fragments followed by a ring‐closure reaction has been a widely used strategy (Figure 1a) [16]. For example, the groups of Itami in 2013 and Martín in 2018 reported heptagon‐embedded NGs, where the seven‐membered rings were realized by the Scholl reaction of corannulene‐based oligophenylenes [17, 18]. In contrast, in 2020, Chi and coworkers reported that Scholl‐type oxidation of naphthalene‐containing oligophenylenes can lead to unexpected structural rearrangements, resulting in azulene‐embedded nanographenes [2]. Similarly, the group of Narita reported a negatively curved framework incorporating a [5]helicene and two heptagons obtained via an aryl rearrangement during the Scholl reaction of a naphthalene‐containing precursor [19]. In the same year, Takasu et al. reported a helical structure incorporating two heptagons via a Scholl reaction of a seven‐membered ring embedded precursor [20]. In 2023, Miao and coworkers showcased a general strategy to form multiple heptagons via Scholl reaction of macrocyclic precursors containing naphthalene moieties [21]. Saddle‐shaped structures, warped by the inclusion of heptagonal rings can also be obtained, as demonstrated independently by the group of Mastalerz and us [7, 8]. In both cases, negatively curved NGs embedded with four heptagons were achieved by the Scholl reaction of precursors containing triphenylene subunits. More recently, Liu et al. demonstrated the synthesis and characterization of a small π‐system containing three consecutive adjourned heptagons facilitated by the Heck reaction of a naphthalene‐containing precursor [22]. In addition, Miao and coworkers reported a NG bearing three heptagons by stepwise Scholl reaction with a triple [6]helicene intermediate [23]. Very recently, Mastalerz and coworkers reported a series of NGs embedded with two heptagons and multiple helicene units in which the odd‐membered rings were pre‐constructed by a Knoevenagel‐type condensation reaction [24]. Despite these significant synthetic achievements, the above heptagon‐embedded NGs are either highly symmetric or exhibit rapid intramolecular dynamics, therefore, making it challenging to achieve configurationally highly stable chirality as well as chirality‐related properties. To address this limitation, the introduction of helical moieties into NGs has emerged as a complementary strategy to modulate their geometry and physicochemical properties [25, 26, 27, 28]. More specifically, helical NGs present intrinsic chirality with distinct dynamic characteristics and exceptional chiroptical responses, holding great potential for applications, for example, circularly polarized luminescence (CPL) or chirality‐induced spin selectivity (CISS) [29, 30, 31, 32, 33, 34, 35]. Therefore, the incorporation of helical motifs in saddle‐shaped π‐systems can endow structures with chiral curvature and exotic chiroptical properties, which remains largely unexplored so far, due to the lack of feasible synthetic strategies.

Herein, we demonstrate a novel synthetic strategy toward a chiral saddle‐shaped NG (**cSNG**, **1**) containing two heptagons (Figure 1b) via a two‐step oxidation reaction of an anthracene‐containing oligophenylene precursor. First, Scholl‐type oxidation yields NG **2** as a key intermediate featuring two *sp* ^3^‐defect heptagons. Interestingly, this Scholl reaction proceeds with diastereoselectivity, exclusively affording the (*S*,*S*)‐**2** and (*R*,*R*)‐**2** enantiomers as a racemic mixture. The subsequent oxidative dehydrogenation furnishes fully conjugated NG **1** incorporating two heptagon‐embedded [4]helicene subunits. The single‐crystal structure of **1** clearly displays two highly bent, saddle‐shaped conformers with depths and widths of 5.8 and 16.9 Å for form **1A** and 6.5 and 15.7 Å for form **1B**, in which two heptagon‐embedded hexabenzocoronene (HBC) units are linked by a single benzene ring, giving rise to a relatively flexible π‐skeleton. Profiting from the heptagon‐embedded [4]helicene and its π‐extension, resultant NG **1** adopts a chiral saddle‐shaped structure with a high isomerization barrier of 42 kcal mol^−1^ (derived by theoretical calculations), thus enabling chiral resolution and subsequent circular dichroism (CD) measurements. Chiroptical characterization of NG **1** revealed pronounced Cotton effects up to 550 nm with a 6.5‐fold amplification of the absorption dissymmetry factor (*g*_abs_), compared with its *sp* ^3^‐defect analogue NG **2**. More interestingly, owing to its unique chiral saddle framework and shape‐adaptive character, NG **1** presents strong host–guest behavior, associating with fullerenes to form a 1:1 complex in solution, as well as a 1:3 complex in the solid state. The determined association constants (from CD titration) were calculated to be 2.3 × 10^4^ M^−1^ for the **1**⊃C_60_ complex and 8.7 × 10^4^ M^−1^ for the **1**⊃C_70_ complex, which are among the highest values for reported fully fused polycyclic aromatic hydrocarbons with negative curvature. These results highlight the potential of this approach for developing future chiral non‐benzenoid nanographenes with pronounced chiroptical properties and intriguing supramolecular behavior.

## 2 Results and Discussion

### 2.1 Synthesis and Characterization

**FIGURE 2:**
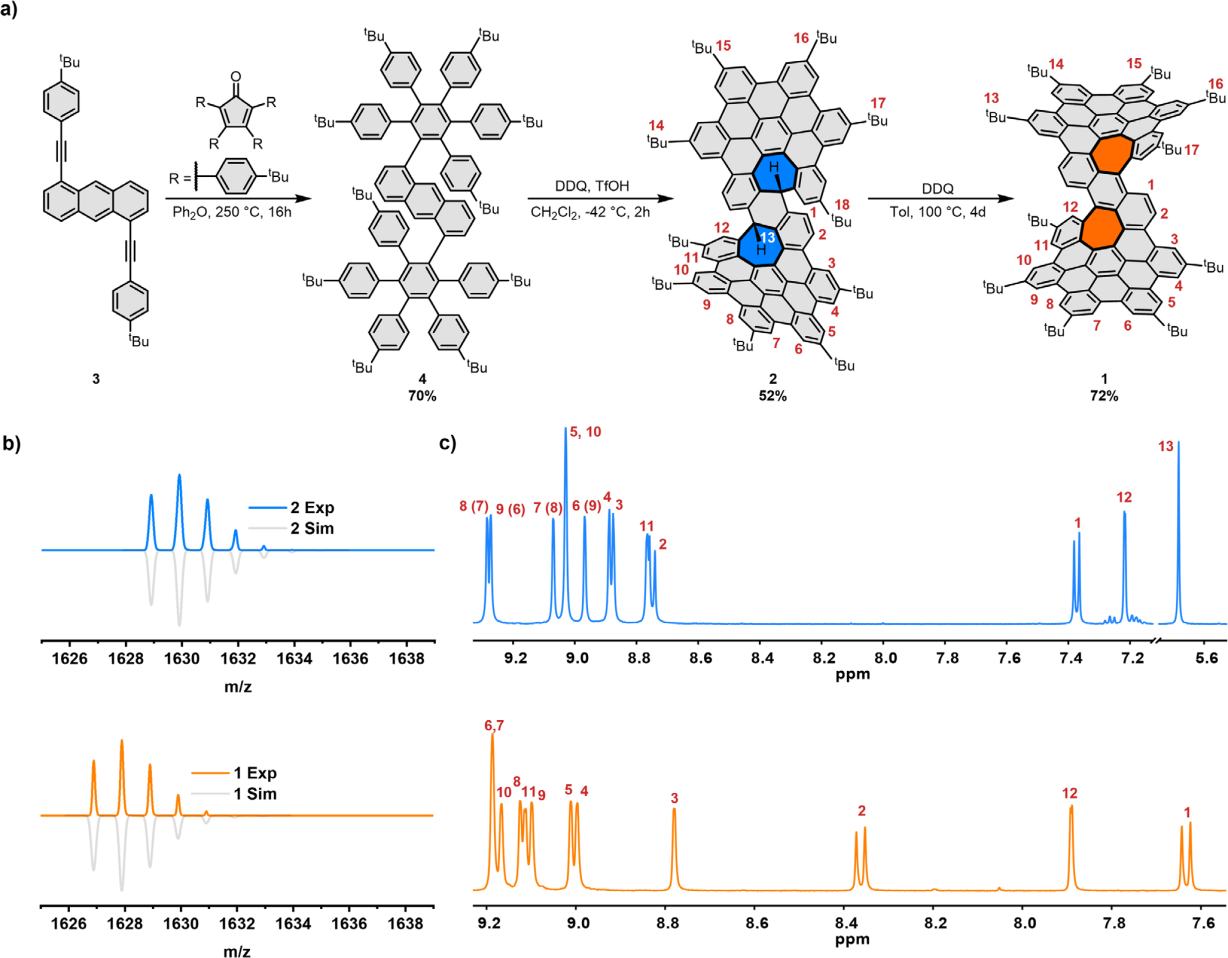
(a) Synthetic route towards sp^3^‐defect NG **2** and target chiral saddle‐shaped NG **1**. (b) HR MALDI‐TOF mass spectra of **2** (top) and **1** (bottom). (c) Aromatic region of the ^1^H NMR spectra of NG **2** (tetrachloroethane‐d_2_, 500 MHz, top) and NG 1 (toluene‐d_8_, 500 MHz, bottom); numbers in parentheses indicate ambiguous assignment.

The synthetic route towards chiral saddle‐shaped NG **1** is depicted in Figure 2a. Commercially available 1,5‐dibromoanthracene was subjected to Sonogashira cross coupling reaction with 1‐(*tert*‐butyl)‐4‐ethynylbenzene giving rise to 1,5‐bis((4‐(*tert*‐butyl)phenyl)ethynyl)anthracene (**3**) in 94% yield. Diels‐Alder [4 + 2] cycloaddition of **3** with 2,3,4,5‐tetrakis(4‐(*tert*‐butyl)phenyl)cyclopenta‐2,4‐dien‐1‐one cleanly afforded oligophenylene **4** in 70% yield. Scholl reaction of precursor **4** with 2,3‐dichloro‐5,6‐dicyano‐1,4‐benzoquinone (DDQ) and trifluoromethylsulfonic acid (TfOH) in dichloromethane (CH_2_Cl_2_) at −42° C afforded a NG containing two *sp* ^3^‐defect heptagons (**2**) in 52% yield. Notably, only a racemic mixture of the (*S,S)‐* **2** and (*R,R)‐* **2** enantiomers was obtained, in which the two hydrogen atoms on the *sp* ^3^‐carbon atoms are positioned on the same side of the molecular skeleton, as determined by single crystal x‐ray diffraction (SCXRD) (see Figure S1). To gain deeper insight into the diastereoselective formation of NG **2**, the reaction mechanism was studied by density functional theory (DFT) calculations (see details in chapter 3.4 in the Supporting Information). The calculated energy difference between the competing transition states (ΔΔ*G* ^‡^) of *syn*‐ and anti‐intermediates is 40 kJ mol^−1^ (Figure S17), corresponding to a syn/anti ratio of ∼ 1 × 10^9^:1, demonstrating a strong kinetic preference for the syn‐pathway, in agreement with the experimentally observed diastereoselectivity. The unsaturated structure encouraged us to further oxidize the system. Therefore, compound **2** was treated with an excess of DDQ in toluene at 100° C to afford fully conjugated NG **1** (yield over four steps: 25%). To better understand the harsh conditions for the oxidative dehydrogenation, DFT calculations at the ωB97X‐D level of theory were conducted, and revealed a high activation barrier of ca. 147 kJ mol^−1^ (Figure S18), consistent with the elevated temperature required. First indications of the successful isolation of target NG **1** and intermediate NG **2** were inferred by high‐resolution matrix‐assisted laser desorption/ionization time‐of‐flight (MALDI‐TOF) mass spectrometry, with the experimental isotopic distribution patterns matching well with the calculated ones (Figure 2b). In addition, the oxidation of **2** to target **1** result in a loss of two *m/z* units in the MALDI‐TOF MS spectrum (Figure 2b). Subsequently, 1D and 2D NMR analyses were performed to validate the chemical structures of both **1** and **2** (Figures S26–S37), resulting in a complete ^1^H signal assignment for both compounds. Characteristic for the *sp* ^3^‐defect embedded NG **2** is the singlet at *δ* = 5.68 ppm (Figure 2c), which was assigned to the protons H_13_ attached to the *sp* ^3^‐hybridized carbon atoms. The observation of the single signal confirms the diastereoselective formation of a racemic mixture of (*S,S)‐* **2** and (*R,R)‐* **2**, excluding the presence of (*R*,*S*)‐**2** species. Apart from the two doublets for the vicinal protons H_1_ and H_2_, only singlets were observed, sometimes broadened or split by small ^4^ *J*_HH_ couplings. As expected from the MALDI‐TOF MS result, the signal of the two *sp^3^*‐CH protons disappears in the spectrum of conjugated chiral saddle NG **1** (Figure S32).

**FIGURE 3:**
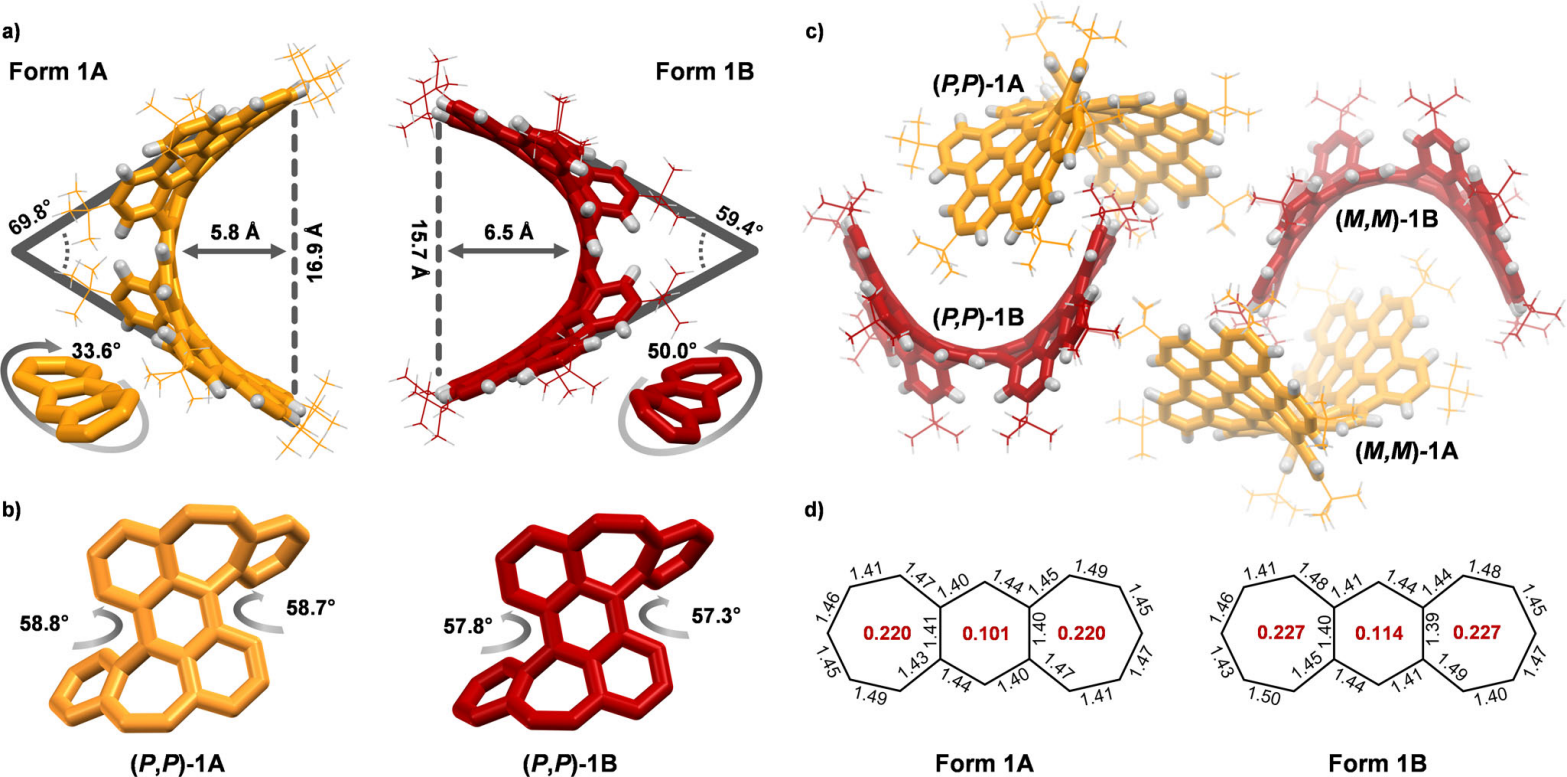
(a) Geometrically different forms **1A** and **1B** as determined by SCXRD. (b) Dihedral angles of (*P,P*)‐**1A** and (*P,P*)‐**1B**. (c) Packing mode of **1**. Aromatic skeleton depicted as capped‐sticks and *tert*‐butyl groups depicted as wireframes; solvent residues and disordered *tert*‐butyl groups are omitted for clarity. (d) Bond length analysis and non‐planarity values of 7‐6‐7 motif in **1A** and **1B**.

Next, the structures of NGs **1** and **2** were unambiguously confirmed by means of single crystal x‐ray diffraction (Figures 3, S1). The crystals of racemic saddle‐shaped NG **1** were obtained by cooling a concentrated solution in CH_2_Cl_2_ to 8 °C for 4 h. NG **1** crystallizes in the triclinic space‐group $P\bar{1}$ with *Z *= 4. Remarkably, there are two crystallographically independent conformers of **1** (**1A**, colored golden; **1B**, colored red) observed from the solid state with markedly different saddle geometries (Figure 3a). Form **1A** displays a width of 16.9 Å, and variant **1B** presents a smaller width of 15.7 Å. These distances were measured between the *tert*‐butyl functionalized C‐atoms C_15_/C_63_ and C_141_/C_189_ (see Figure S5), respectively. The bent π‐scaffold of **1** spans an angle of 69.8° for **1A** and 59.4° for **1B**, with a remarkable difference of 10.4° between the two forms. The depth of the respective saddles was determined by measuring the distance from the bottom of the saddle to the centroid of the central six‐membered ring in the anthracene fragment (detailed information in the determination of the above parameters is provided in Chapter 2 in the Supporting Information). Accordingly, form **1A** possesses a depth of 5.8 Å, while form **1B** displays a depth of 6.5 Å, demonstrating that NG **1** is a deep saddle, similar to previously reported seven‐membered ring incorporated nanographene saddles [8, 36]. Further, the anthracene core of the two forms displays significantly different end‐to‐end twists (Figure 3a), with a distortion angle of 33.6° for form **1A**, and 50.0° for form **1B**. The heptagon‐embedded [4]helicenes display dihedral angles between the terminal rings of 58.7°/58.8° in form **1A** and 57.3°/57.8° in form **1B** (Figure 3b), much larger than that of pristine benzenoid [4]helicene (26°) [37]. This increase can be attributed to the nonplanarity of the embedded seven‐membered rings. Notably, in the unit cell there are two pairs of **1A** and **1B** of which each pair consists of two enantiomers, that is, *(M,M*)‐**1A** and (*M,M*)‐**1B**, as well as *(P,P*)‐**1A** and (*P,P*)‐**1B** (Figure 3c). According to non‐planarity analysis [7, 36, 38] of individual rings in the chiral saddle (Figure 3d), the three‐dimensional incurvation is mainly expressed by the seven‐membered rings (0.220 in form **1A**, 0.227 in form **1B**) and by the central benzene ring in the anthracene fragment (0.101 in form **1A**, 0.114 in form **1B**) (for a full list of non‐planarity values see Figure S6 and Table S5). Regardless of the differences in width, depth, bending angle, and twist of the saddle core, the bond lengths in the central 7‐6‐7 moiety are essentially unchanged between **1A** and **1B** (Figure 3d). The maximum deviations between corresponding bonds are below 0.01 Å (≈ 1 pm), that is, on the order of ≤ 0.7% and comparable to the experimental uncertainty of the refinement. Thus, the pronounced differences in the global saddle geometry do not arise from significant changes in local bonding, but rather from variations in torsion angles and ring orientations around the relatively flexible 7‐6‐7 hinge. These observations suggest that **1** possesses a flexible π‐skeleton, which can be attributed to the narrow connection (only one benzene‐ring width) in the center of the molecule.

### 2.2 Density Functional Theory (DFT) Calculations

**FIGURE 4:**
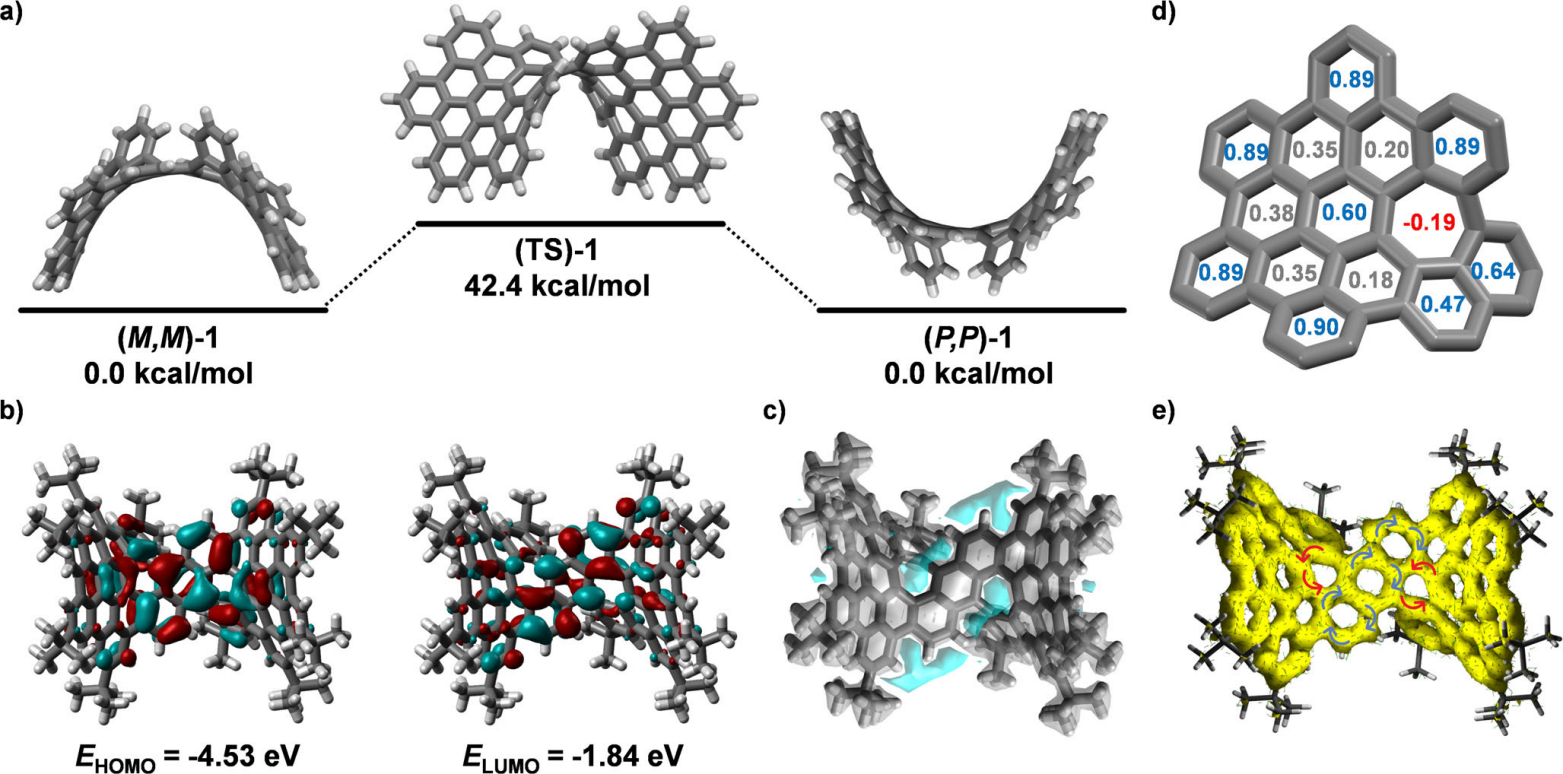
(a) Gibb's energies of isomerization process of **1** calculated at the basis set B3LYP/6‐31G(d). *Tert*‐butyl groups omitted for calculation simplicity. (b) FMOs of NG **1**, isosurface values set at 0.02. (c) ICSS*_zz_*‐plot for **1**, gray represents positive magnetic shielding and cyan represents deshielding; isovalues set at 12.0. (d) HOMA values of DFT optimized structure of **1**. Blue: aromatic; gray: slightly aromatic/non‐aromatic; red: antiaromatic. Only asymmetric unit shown; H‐atoms and *tert*‐butyl groups omitted for clarity. (e) ACID plot of **1**; red arrows: paratropic ring currents, blue arrows: diatropic ring currents; isovalues set at 0.05; the direction of the magnetic field is orthogonal to the XY plane and points upward.

The racemization barrier of saddle **1** was investigated by means of transition state calculations at the B3LYP/6‐31G(d) level of theory (Figure 4a). The conversion of chiral saddle (*M,M*)‐**1** to (*P,P*)‐**1** surprisingly goes through a twisted transition state (TS)‐**1** where both helicene subunits (see Figure 3b for chiral unit) reverse their chirality at the same time. A racemization barrier of 42.4 kcal/mol was determined, which is significantly higher than those reported for related [4]helicene‐containing nanographenes (4‒12.2 kcal/mol) [39], suggesting high configurational stability for this π‐extended heptagon‐embedded [4]helicene. Therefore, the resolution of enantiomers from inherently chiral saddle **1** was feasible via high performance liquid chromatography (HPLC) (Figure S58). In addition, DFT calculations reveal that the frontier molecular orbitals (FMOs) of NG **1** are concentrated around the central anthracene fragment and on the seven‐membered rings (Figures 4b, S11). To enrich our understanding of the aromaticity of the embedded heptagonal rings, the isochemical shielding surface in *z*‐direction (ICSS_zz_) of **1** was calculated (Figure 4c) [40]. As depicted, the seven‐membered rings are significantly deshielded, revealing their antiaromatic character. Meanwhile, the residual π‐skeleton displays positive magnetic shielding. In addition, the harmonic oscillator model of aromaticity (HOMA) calculation also demonstrates the slight antiaromaticity of the seven‐membered ring (Figure 4d). Furthermore, anisotropy of the induced current density (ACID) calculations was conducted (Figure 4e). Counterclockwise paratropic ring currents were observed for the seven‐membered rings, further substantiating the antiaromatic character of the heptagons. These results are in line with previously reported heptagon‐embedded graphenoid molecules [7, 8].

### 2.3 Optoelectronic Properties

**FIGURE 5:**
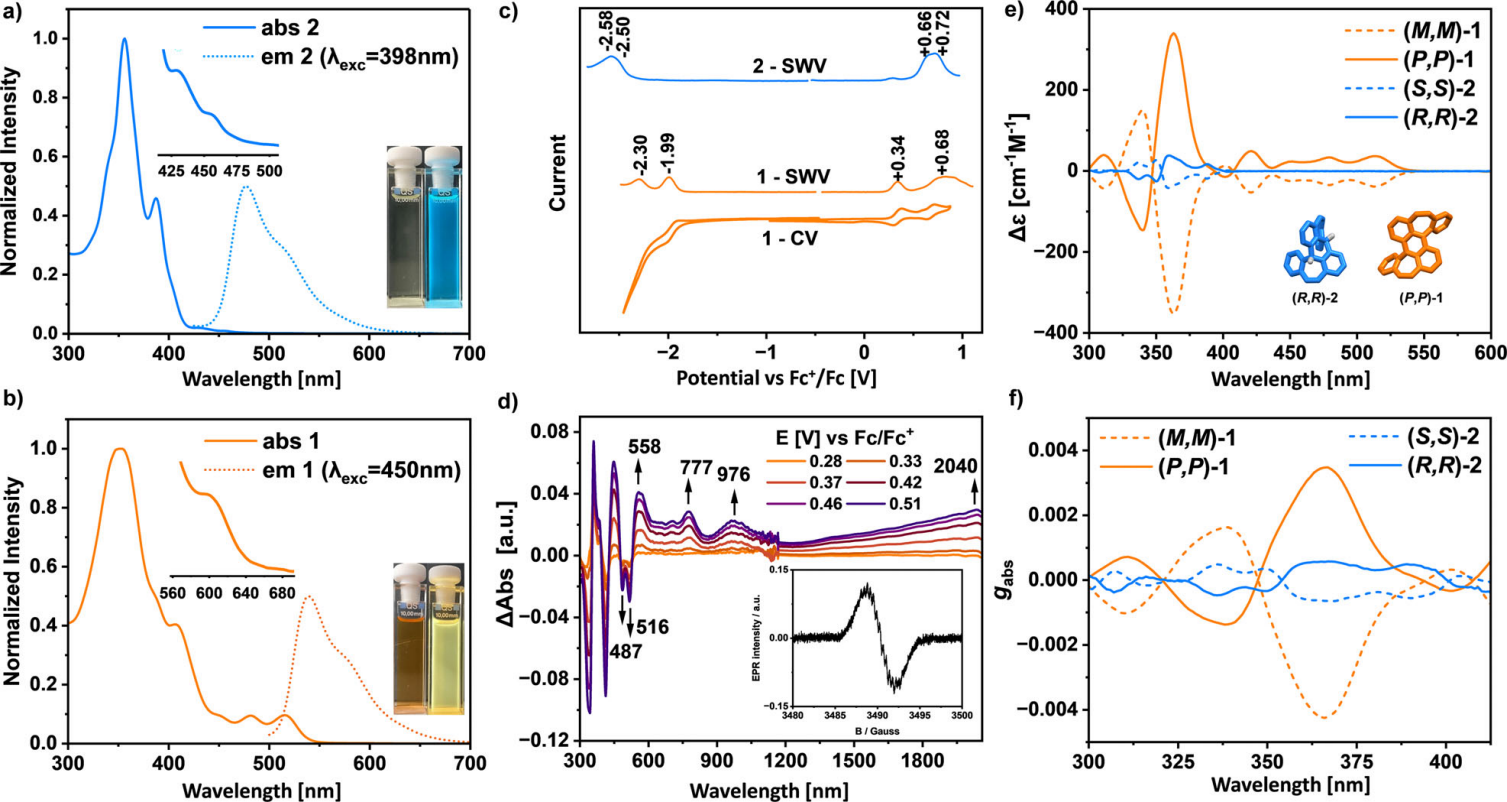
UV–vis absorption (solid lines) and photoluminescence (dotted lines) of **2** (a) and **1** (b); inserts: respective solutions under ambient (left) and UV light (300 nm, right). (c) SWV spectra of **2** as well as SWV and CV spectra of **1**. (d) Spectroelectrochemical characterization of the radical cation **1^•+^**; inset EPR spectrum. (e) CD spectra of chiral NGs **1** and **2**. (f) Dissymmetry factor over wavelength of NGs **1** and **2**. All measurements were performed in CH_2_Cl_2_ solution at room temperature.

To investigate the optical properties of both heptagon‐embedded molecules, UV–vis absorption and photoluminescence experiments of NGs **1** and **2** were performed in CH_2_Cl_2_. Compared to **2** containing two defective *sp* ^3^‐C atoms (Figure 5a), NG **1** presents an obviously redshifted absorption and luminescence due to the extended conjugated skeleton (Figure 5b). Compound **2** shows a maximum absorption peak (λ_max_) at 455 nm with tailing up to 500 nm (see inset in Figure 5a), corresponding to an optical energy gap of 2.61 eV (tangent method). The photoluminescence displays a maximum emission at 477 nm, with a photoluminescence quantum yield (PLQY) of 6.3%. Notably, the optical properties of NG **2** are similar to a spiro‐fused hexa‐*peri*‐hexabenzocoronene dimer (energy gap: 2.63 eV; emission peak: 467 nm, PLQY: 6.3%) [41], which indicates the non‐conjugated nature of **2** attributed to the presence of the *sp* ^3^‐hybridized carbon atoms in the heptagons. Meanwhile, chiral saddle **1** displays a λ_max_ at 597 nm with tailing up to 690 nm (see inset in Figure 5b), resulting in a reduced optical energy gap of 1.94 eV. In addition, **1** emits yellow fluorescence with a PLQY of 20.2% and emission peak at 539 nm, further demonstrating its enhanced π‐conjugation.

Moreover, the electrochemical properties of NGs **1** and **2** were also studied by cyclic voltammetry (CV) and square wave voltammetry (SWV) in anhydrous CH_2_Cl_2_ (Figure 5c). The cyclic voltammetry experiment for *sp* ^3^‐defect embedded NG **2** showed irreversible oxidations and reductions located near the limit of the electrolyte's electrochemical window. The SWV data of **2** is plotted with two reductions at peak potential of −2.58 and −2.50 V and two oxidations at +0.66 and +0.72 V versus Fc^+^/Fc. Meanwhile, chiral saddle **1** displays two reversible oxidations at half‐wave potential of +0.34 and +0.68 V and two reversible reductions at ‐1.99 and −2.30 V. Accordingly, the electrochemical energy gap of **2** and **1** is calculated to be 2.60 and 1.97 eV, respectively, based on the onset potentials of their first oxidation (+0.40 V for **2**; +0.15 V for **1**) and first reduction (−2.20 V for **2**; −1.82 V for **1**) waves (see chapter 4 in the Supporting Information). The electrochemical energy gaps are in alignment with the optical ones determined from UV–vis spectroscopy.

Considering the lower oxidation potential of chiral saddle **1**, its redox behavior was further studied by in situ electron paramagnetic resonance (EPR)/UV‐Vis‐NIR spectroelectrochemistry (SEC) (Figure 5d). Upon the first oxidation process, in situ EPR spectra reveal a broad signal with a *g* value of 2.0026 and a hyperfine structure. Unfortunately, the analysis of the spectrum was not possible due to its low quality. The appearance of the EPR signal (inset Figure 5d) unambiguously confirms the formation of a radical cationic π‐extended species [25, 42]. The absorption bands at 558, 777, 976, and 2040 nm occur in the UV‐Vis‐NIR spectra together with the EPR signal and can be, therefore, assigned to the radical cation. Likely, the radical cation will be stabilized by the formation of formal tropylium moieties [7, 8, 43].

Upon successful separation of the enantiomers of both NGs **1** and **2** by chiral HPLC (see Figures S57 and S58), their chiroptical properties were investigated by CD spectroscopy. As shown in Figure 5e, the enantiomers of NG **2** display a series of mirror‐image Cotton effects up to 400 nm, indicating chirality transfer from the *sp* ^3^‐C to the π‐framework. In sharp contrast, the enantiomers of NG **1** present pronounced Cotton effects up to 550 nm, with a significant increase in chiroptical response at λ = 363 nm (Δ*ε*: ‐352 M^−1^ cm^−1^ for **1** and −37 M^−1^ cm^−1^ for **2**), profiting from the full conjugation of the helical unit (helicene subunits of **1** and **2** can be found as insets in Figure 5e). According to the equation of the absorption dissymmetry factor (*g*_abs_ = Δ*ε/ε*), the relationship between *g*_abs_ and wavelength is described in Figure 5f. Compared to *sp* ^3^‐defect **2** featuring a maximum *g*_abs_ of 6.5 × 10^−4^ at 366 nm, the *g*_abs_ value of conjugated saddle **1** was found to be amplified to 4.2 × 10^−3^ at 366 nm, with a remarkable 6.5‐fold magnification. To gain a deeper understanding of the significant increase in chiroptical response, TD‐DFT calculations were performed at the B3LYP/6‐31G(d) level of theory (see Figures S13–S15). The calculation results reveal that the CD response of *g*_abs,max_ can be assigned to the S_0_→S_17_ excitation for **1** and to the S_0_→S_13_ excitation for **2** (Table S8). According to the formula for the theoretical dissymmetry factor *g*_calc_ = 4 cos(*θ*) × |*µm*|/|*µ_e_* |, the ratio between the magnetic and electric transition dipole moments |*µm*|/|*µ_e_* | plays an important role in the determination of the *g*_calc_ value. Therefore, the drastic increase in the |*µm*|/|*µ_e_* | ratio from **2** to **1**, corresponding to a fivefold enhancement, significantly contributes to the observed increase in *g*_calc_ value. To further investigate the potential of chiral saddle **1** as a CPL emitter, the CPL spectra of the isolated enantiomers were also recorded in anhydrous CH_2_Cl_2_ (Figure S19). Enantiomers of **1** present pronounced luminescence responses with mirror‐like images in the range of 500–700 nm, with *g*_lum_ values of approximately 0.003 for (*M*,*M*)‐**1** and ‐0.003 for (*P*,*P*)‐**1** at 550 nm, highlighting its potential in chiral optoelectronics. In addition, the *g*_abs_ and *g*_lum_ values of chiral saddle **1** and related chiral nanographenes are summarized in Table S11.

### 2.4 Host–Guest Interaction With Fullerenes

**FIGURE 6:**
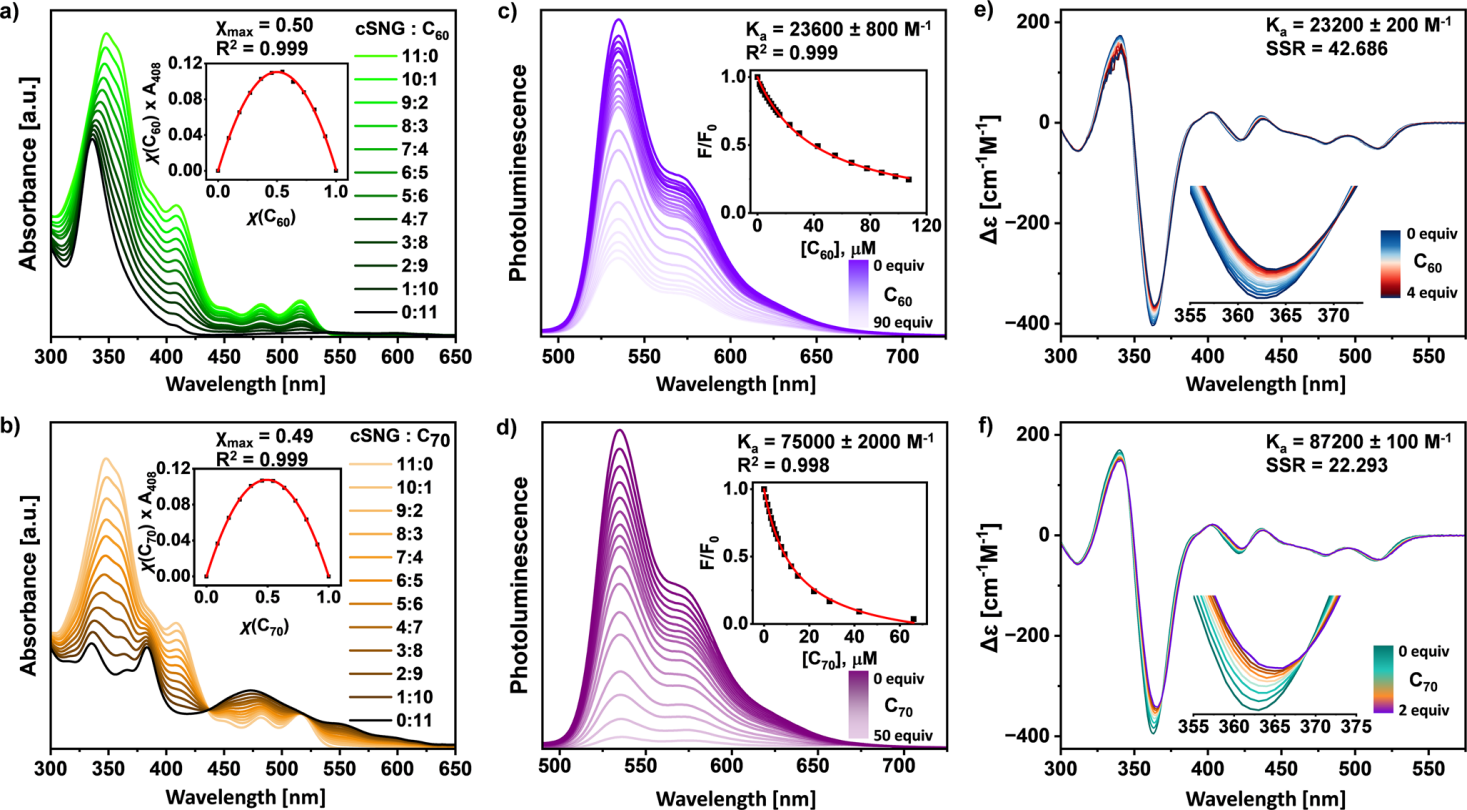
Stacked UV–vis spectra of NG **1** with varying amounts of C_60_ (a) and C_70_ (b). Stern‐Volmer titrations of **1** with C_60_ (c) and C_70_ (d). Stacked CD spectra of **1** in the presence of varying amounts of C_60_ (e) and C_70_ (f). All measurements were performed in toluene solution at room temperature.

The flexible conformation of chiral saddle **1** provides highly shape‐adaptive character, rendering it a promising candidate for host–guest chemistry experiments. To this end supramolecular interactions of chiral saddle **1** with fullerenes were investigated both in solution and solid state. First, the binding mode of the host–guest interaction with the respective fullerene was analyzed by means of Job's plot analysis (Figure 6a,b). Based on UV–vis titration experiments of a toluene solution of **1** (approximately 10 µM) with varying concentrations of C_60_ or C_70_, Job's plots were constructed, revealing a 1:1 association ratio between the saddle host and the fullerene guest in the micromolar solution.

Furthermore, Stern‐Volmer analyses were conducted to determine the binding constants (*K*_a_) (Figure 6c,d). For this, solutions of chiral saddle **1** (approximately 1 µM) were titrated with increasing amounts of fullerenes C_60_ and C_70_, respectively, and the emission intensity was monitored. Accordingly, the corresponding *K*_a_ was determined by a non‐linear fit (see Equation 3 in the Supporting Information), with a *K*_a_ of 2.36 ± 0.08 × 10^4^ M^−1^ for **1**⊃C_60_ and a *K*_a_ of 7.50 ± 0.20 × 10^4^ M^−1^ for **1**⊃C_70_.

In addition, titrations of chiral saddle **1** with both C_60_ and C_70_ with concurrent CD measurements were carried out (concentration of host approximately 10 µM in toluene). Compared with fluorescence titration, CD titration is inherently sensitive to chiral environments and is able to exclude the interference from spectral overlap and fluorescence quenching, thereby allowing a more accurate estimation of *K*_a_. Upon stepwise addition of 0.25 equivalents of C_60_/C_70_, the corresponding CD responses were recorded with an obvious redshift for the Cotton effects across the measured wavelength region, indicating an alteration in the electronic structure of the host molecule (Figure 6e,f). The Δε values in the range of 367–362 nm was used to determine the *K*_a_ of the interaction between **1** and the respective fullerene (see Figures S24 and S25). Accordingly, the *K*_a_ was derived to be 2.32 ± 0.02 × 10^4^ M^−1^ for **1**⊃C_60_ and 8.72 ± 0.01 × 10^4^ M^−1^ for **1**⊃C_70_, which are among the highest values for reported fully fused polycyclic aromatic hydrocarbons with negative curvature (Table S18) [7, 8, 11, 18, 22, 44, 45, 46]. The higher binding affinity observed for the **1**⊃C_70_ complex relative to **1**⊃C_60_ is likely due to a larger π‐overlap between host and guest, and the slightly higher electron affinity of C_70_ [7, 8]. This remarkable host–guest interaction might be explained by the previously discussed flexibility of the π‐system, which reflects the ability of the host framework to adapt its conformation to accommodate the guest species.

To further evaluate the adaptability of the chiral saddle with fullerene guests, ^1^H NMR titration experiments of **1** with both C_60_ and C_70_ (host concentrations of 1  and 0.33 mM in toluene‐d_8_, respectively) were conducted (Figures S21 and S23). Fitting the obtained chemical shift displacement values for the titration with C_60_ (Table S14) with BindFit v0.5 [47] to a 1:1 model, a low *K*_a_ value was obtained (2.8 ± 0.2 × 10^3^ M^−1^). This is about one order of magnitude lower than the binding constant previously determined via CD titration and was therefore ruled out. Applying a 1:2 fit gave binding constant values of *K*_11_ = 2.10 ± 0.36 × 10^4^ M^−1^ and *K*_12_ = 1.8 ± 0.1 × 10^2^ M^−1^ (see Figure S20). The much lower *K*_12_ relative to *K*_11_ indicates a very weak secondary binding site. Similarly, the NMR titration with C_70_ (Table S15) revealed a 1:2 complex with binding constants calculated to 8.42 ± 0.77 × 10^4^ M^−1^ for *K*_11_ and 2.4 ± 0.1 × 10^2^ M^−1^ for *K*_12_. For NMR titration with both C_60_ and C_70_, *K*_11_ is in agreement with the determined results from CD titration (Figure 6e,f). The binding strength is illustrated by the plot of the molar fractions of **1**, **1**⊃C_70_, as well as **1**⊃(C_70_)_2_ against the equivalents of C_70_ (Figure S22). Upon addition of one equivalent of fullerene, more than 80% of chiral saddle **1** is transformed to **1**⊃C_70_. However, even with four equivalents of C_70_, the molar fraction of **1**⊃(C_70_)_2_ is less than 20%, demonstrating the weak second binding site in solution. Notably, the binding constants obtained from several independent titration methods (fluorescence quenching, CD titration, and NMR titration) over a broad concentration range (from approximately 1 µM to 1 mM) are in good agreement with each other, highlighting the robustness and reproducibility of the determined values.

**FIGURE 7:**
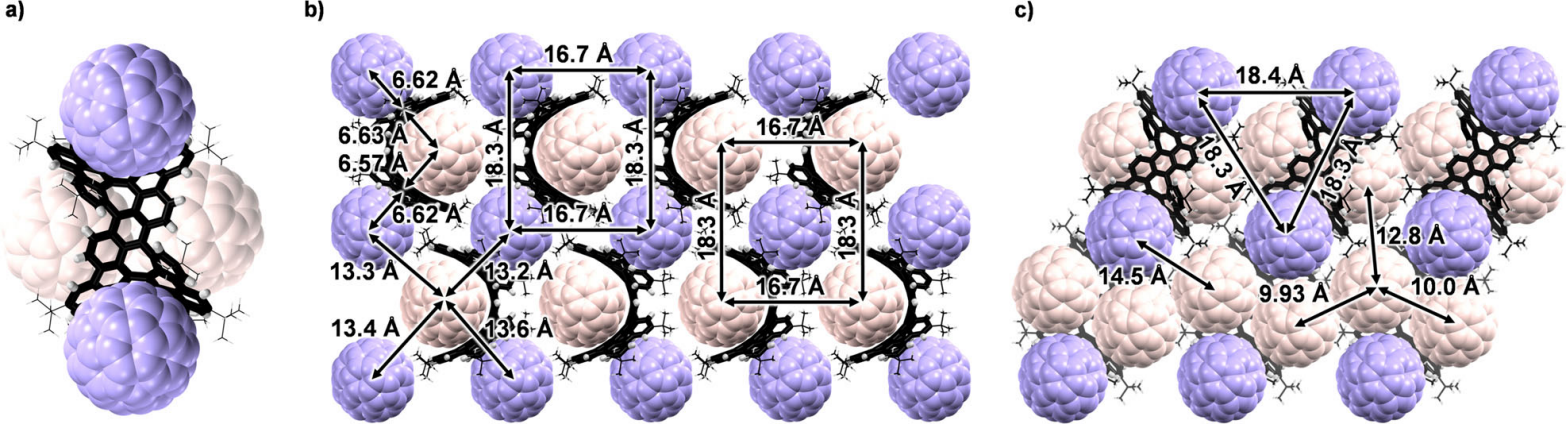
(a) View of saddle **1** associated with four individual C_60_ along crystallographic *b*‐axis. (b) Crystalline packing of **1**⊃(C_60_)_3_; fullerene–saddle and fullerene–fullerene distances depicted. (c) Staggered view of packing along the crystallographic *b*‐axis; fullerene–fullerene distances between stacks of 1D strands depicted. Saddle **1** depicted as capped sticks with *tert*‐butyl groups as wireframe; C_60_ molecules depicted as space‐fill models; C_60_ molecules located inside the saddle cavity in off‐white, C_60_ molecules located outside the saddle cavity in lavender.

To gain further insight into the binding mode in the solid state, the supramolecular complexation was investigated by cocrystal analysis. Slow diffusion of hexane into a toluene solution of the mixture of chiral saddle **1** and C_60_ (ratios ranging from 1:1, 1:2, and up to 1:3.4) exclusively gave single crystals of the supramolecular complex as **1**⊃(C_60_)_3_. The structure crystallized in the space‐group P2/n with *Z *= 2 and *Z’ *= 0.5. Driven by solid‐state packing effects, each chiral saddle has been found to associate with four individual molecules of C_60_, with two buckyballs accommodated inside the saddle cavity (off‐white colored), while the other two (lavender colored) are shared over the concave outer faces of the π‐skeleton to the neighboring saddles (Figure 7a). Structural analysis of the saddle in complex revealed a depth of 6.7 Å (pristine saddle: 5.8/6.5 Å), and a width of 14.9 Å (pristine saddle: 16.9/15.7 Å), suggesting a more curved geometry in the supramolecular complex. This finding further highlights the flexible and shape‐adaptive nature of chiral saddle **1**. The distance of the centroid of the bridging fullerene molecule to the concave π‐system is 6.62 Å on both sides of the molecule. This corresponds to a π–π distance of 3.16 Å, indicating strong interactions between the concave and convex shapes. The distances of the cavity‐facing fullerene molecules to the π‐system are 6.63 Å and 6.57 Å (π–π distances of 3.18 and 3.08 Å, respectively) (Figure 7b upper left corner).

The individual **cSNG** molecules are organized in alternating 1D strands of homochiral stacks forming a pseudo‐2D sheet (Figure 7b). Remarkably, the bridging fullerenes (colored in lavender) and the cavity‐facing fullerenes (colored in off‐white) establish two individual rectangles of the same dimensions. The edges parallel to the strands have a length of 16.7 Å, while the edges perpendicular to the stacks display a length of 18.3 Å. This finding can be interpreted as the coexistence of two individual sublattices of fullerenes in the crystal structure. One is formed by the lavender‐colored fullerenes, the other is formed by the off‐white‐colored fullerenes. The distances between a cavity‐facing fullerene and the bridging fullerenes accompanying the respective saddle are in the range between 13.2 and 13.6 Å. This means that the sublattices are slightly displaced relative to each other by the presence of the chiral saddle‐shaped structure. When considering the distances between bridging fullerenes of different layers, an almost equilateral triangle can be observed (Figure 7c). The edges of this triangle show lengths of 18.3 to 18.4 Å. On the other hand, the cavity‐facing fullerenes form 2D zigzag chains connecting individual layers. Noteworthy, the fullerenes inside a saddle cavity are slightly further apart (10.0 Å) than the fullerenes of two neighboring saddles (9.93 Å). The smallest distance between individual zigzag chains is 12.8 Å. The closest arrangement of a bridging fullerene and a cavity‐facing fullerene of separate saddles is 14.5 Å. All of the above values are measured by the centroid–centroid distances of the respective fullerenes.

## 3 Conclusion

In summary, we developed a novel synthetic strategy toward a chiral saddle‐shaped nanographene via a two‐step oxidation reaction of an anthracene‐containing oligophenylene precursor. The crystal structure unambiguously revealed its saddle‐shaped geometries with inherent chiral heptagon‐containing [4]helicene subunits. The high isomerization barrier of the chiral saddle allows chiral resolution and chiroptical properties investigation, revealing a 6.5‐fold amplification of the *g*_abs_ value, compared to that of the *sp* ^3^‐defect analogue. Noteworthy, chiral saddle **1** exhibits significant flexibility in its π‐skeleton with shape‐adaptive character, thus giving rise to high binding constants of 2.3 × 10^4^ M^−1^ for C_60_ and 8.7 × 10^4^ M^−1^ for C_70_, superior to reported NGs. Moreover, cocrystal structure analysis revealed a remarkable **1**⊃(C_60_)_3_ complex in the solid state. This work provides an effective strategy to synthesize a saddle‐shaped NG with stable chirality and pronounced chiroptical responses, which may offer a potential platform to develop chiral nanoelectronics in the future.

## Author Contributions

**Felix Trautner**: Writing – original draft, conceptualization, and investigation. **Hartmut Komber**: Writing – review and editing, and investigation. **Boris Borrisov**: Writing – original draft, and investigation. **Hongqing Zhao**: Investigation. **Philipp Royla**: Writing – review and editing, investigation. **Li Wan**: Investigation. **Jan J. Weigand**: Writing – review and editing. **Evgenia Dmitrieva**: Writing – review and editing, investigation. **Wenhui Niu**: Supervision, writing – review and editing. **Qun Yang**: Investigation. **Ji Ma**: Writing – review and editing. **Xinliang Feng**: Supervision, writing – review and editing.

## Conflicts of Interest

The authors declare no conflicts of interest.

## Supporting information




**Supporting File 1**: Experimental section, ^1^H, ^13^C NMR and 2D NMR spectra, single crystal information, DFT calculations and additional supporting figures/tables. The authors have cited additional references within the Supporting Information.

**Supporting File 2**: anie72921‐sup‐0002‐DataFile.zip.

## Data Availability

The data that support the findings of this study are available from the corresponding author upon reasonable request.
