# Supplementary material for: A Chiral Saddle‐Shaped Nanographene With Two Heptagon‐Embedded [4]Helicenes
Source: Angew Chem Int Ed Engl. 2026 Jun 2;65(31):e3481279. doi: 10.1002/anie.3481279 (PMC13411393; doi:10.1002/anie.3481279)
Supplement: Supplementary file 1 — Supporting File 1: Experimental section, 1H, 13C NMR and 2D NMR spectra, single crystal information, DFT calculations and additional supporting figures/tables. The authors have cited additional references within the Supporting Information. [file ANIE-65-e3481279-s001.docx]

**Supporting Information**

A Chiral Saddle-shaped Nanographene with Two Heptagon-Embedded [4]Helicenes

Felix Trautner^[a],[b]^, Boris Borrisov^[b]^, Philipp Royla^[c]^, Hongqing Zhao^[d]^, Hartmut Komber^[e]^, Li Wan^[a]^, Evgenia Dmitrieva^[f]^, Ji Ma^[g]^, Jan J. Weigand^[c]^, Qun Yang^[a]^, Xinliang Feng^[a],[b],^*, Wenhui Niu^[a],[b],^*

^[a]^ Max Planck Institute of Microstructure Physics, Weinberg 2, 06120 Halle, Germany

^[b]^ Center for Advancing Electronics Dresden (cfaed) & Faculty of Chemistry and Food Chemistry, Technische Universität Dresden, Mommsenstrasse 4, 01062 Dresden, Germany

^[c]^ Chair of Inorganic Molecular Chemistry, Faculty of Chemistry and Food Chemistry, Technische Universität Dresden, 01062 Dresden, Germany

^[d]^ Faculty of Chemistry and Pharmacy, Ludwig-Maximilian-Universität München

^[e]^ Leibniz-Institut für Polymerforschung Dresden e. V., Hohe Straße 6, 01069 Dresden, Germany

^[f]^ Leibniz Institute for Solid State and Materials Research (IFW) Dresden, Helmholtzstr. 20, 01069 Dresden, Germany

^[g]^ College of Materials Science and Optoelectronic Technology & Center of Materials Science and Optoelectronics Engineering, University of Chinese Academy of Science, 100049 Beijing, China

**Contents**

[**1. Experimental section** 2](#_Toc227670083)

[**1.1. General remarks** 2](#_Toc227670084)

[**1.2 Synthetic procedures and characterization data** 5](#_Toc227670085)

[**2. X-ray crystallographic data** 10](#_Toc227670086)

[**3. DFT calculations** 17](#_Toc227670087)

[**4. Cyclic voltammetry (CV) and square wave voltammetry (SWV)** 33](#_Toc227670088)

[**5. CPL experiments** 34](#_Toc227670089)

[**6. UV-Vis titration experiments** 37](#_Toc227670090)

[**7. Stern-Volmer titration experiments** 38](#_Toc227670091)

[**8. NMR titration experiments** 39](#_Toc227670092)

[**9. CD titration experiments** 45](#_Toc227670093)

[**10. Comparison of binding properties with reported molecules** 48](#_Toc227670094)

[**11. ^1^H and ^13^C NMR spectra and HR MS spectra** 50](#_Toc227670095)

[**12. Chiral resolution** 66](#_Toc227670096)

[**13. References** 67](#_Toc227670097)

# **1. Experimental section**

## **1.1. General remarks**

Unless otherwise noted, commercially available starting materials, reagents, catalysts, and dry solvents were used without further purification. Reactions were performed using standard vacuum-line and Schlenk techniques. All starting materials were obtained from TCI, Sigma Aldrich, BLD, Alfa Aesar, Acros Organics, or Fluorochem. Catalysts were purchased from Strem. All reactions dealing with air- or moisture-sensitive compounds were carried out in a dry reaction vessel under argon (Ar) atmosphere by using standard vacuum-line and Schlenk techniques. Anhydrous dichloromethane was obtained from MBRAUN MB-SPS-5 solvent purification system. Column chromatography was performed on silica (SiO2, particle size 0.063-0.200 mm, purchased from VWR). Silica-coated aluminum sheets with a fluorescence indicator (TLC silica gel 60 F254, purchased from Merck KGaA) were used for thin layer chromatography. Recycling gel permeation chromatography (rGPC) was carried out on a Japan Analytical Industry JAI-HPLC LC 9110 II Next equipped with a JAIGEL-2HH and a JAIGEL-1HH column. Chloroform was used as the eluent in a flow rate of 5 mL/min. NMR data were recorded on a Bruker AV-II 300 spectrometer operating at 300 MHz for ^1^H and 75 MHz for ^13^C, on a Bruker Avance III 500 spectrometer operating at 500 MHz for ^1^H and at 125 MHz for ^13^C, and on a Bruker Avance neo spectrometer operating at 600 MHz for ^1^H and at 150 MHz for ^13^C using standard Bruker pulse programs. The measurements were carried out at 23 °C if not stated otherwise. Chloroform-d (*δ*(^1^H) = 7.26 ppm, *δ*(^13^C) = 77.2 ppm), tetrachloroethane-d_2_ (*δ*(^1^H) = 5.98 ppm, *δ*(^13^C) = 73.7 ppm), dichlormethane-d_2_ (*δ*(^1^H) = 5.32 ppm, *δ*(^13^C) = 54.0 ppm), and toluene-d_8_ (methyl group: *δ*(^1^H) = 2.09 ppm, *δ*(^13^C) = 20.4 ppm) were used as solvents and internal chemical shift reference. The ^1^H NMR titration experiments were performed in toluene-d_8_ at 23 °C. The following abbreviations are used to describe peak patterns as appropriate: s = singlet, d = doublet, dd = doublet of doublets, and t = triplet.

High resolution matrix-assisted laser desorption/ionization time-of-flight mass spectra (HR-MALDI-TOF MS) were recorded on a Bruker Autoflex Speed MALDI-TOF MS (Bruker Daltonics, Bremen, Germany). All of the samples were prepared by mixing the analyte and the matrix trans-2-[3-(4-tert-butylphenyl)-2-methyl-2-propenylidene] malononitrile (DCTB, purchased from Sigma Aldrich, purity > 99%) in the solid state. UV-visible-near-infrared (UV-Vis-NIR) spectra were measured on a Shimadzu UV-3600i Plus spectrophotometer by using 10 mm optical-path quartz cell at room temperature. Fluorescence spectra were recorded at room temperature on a PerkinElmer Fluorescence Spectrometer LS 55 using a 10 mm fluorescence quartz cell. Absolute quantum yields were measured at room temperature on a C11347-01 absolute PL Quantum Yield Spectrometer from Hamamatsu Photonics company. Electronic circular dichroism (ECD) and circularly polarized luminescence (CPL) were recorded in an Olis DSM172 spectrophotometer equipped with a non-ozone producing xenon lamp of 150 W.

Cyclic voltammetry (CV) and Square-wave voltammetry (SWV) were carried out on a PARSTAT4000 potentiostat (Princeton Applied Research, Ametek, Germany) in a three-electrode cell in degassed dry dichloromethane solution containing 0.1 M of tetra-*n*-butylammonium hexafluorophosphate (*n*-Bu4NPF6) as a supporting electrolyte. Pt electrodes were used as the working and counter electrode, and a AgCl-coated silver wire as the reference electrode. All potentials are given versus Fc+/Fc redox couple as an internal standard. The electrochemical measurements were performed under inert (nitrogen) atmosphere and at ambient temperature.

For SEC measurements UV−Vis−NIR spectra were measured using the Avantes spectrometer AvaSpec-2048×14-USB2 with the CCD detector and AvaSpec-NIR256-2.2 with the InGaAs detector (Avantes, The Netherlands). A light source Avantes Avalight-DH-S-BAL was used. The UV−Vis−NIR spectrometer is linked to a HEKA potentiostat PG 390 which triggers the spectrometer. At ambient temperature, the UV−Vis−NIR spectra were collected at a continuous potential scan rate (ca. 5 mV/s). Each UV−Vis−NIR spectrum was collected relative to that of the neutral (uncharged) compound. In spectroelectrochemical experiments, an EPR flat cell with a laminated gold μ-mesh (Goodfellow, UK) as working electrode, an AgCl-coated silver wire as reference electrode, and a platinum wire as counter electrode was used. The cell assembling was done under an inert (nitrogen) atmosphere.

For SCXRD, suitable single crystals were coated with Paratone-N oil, mounted using a nylon loop and frozen in a cold nitrogen stream. Crystals were measured at 100 K on a Rigaku Oxford Diffraction SuperNova system using Cu K_α_ radiation (*λ* = 1.54184 Å) generated by a Nova micro-focus X-ray source. Reflections were collected with an Atlas S2 detector. Data reduction and absorption correction was performed with CrysaAlisPro^1^ software. Using Olex2^2^, the structures were solved with SHELXS/T^3^ by direct methods and refined with SHELXL^4^ by least-square minimization against *F*^2^ using first isotropic and later anisotropic thermal parameters for all non-hydrogen atoms. Hydrogen atoms bonded to carbon atoms were added to the structure models on calculated positions using the riding model. All other hydrogen atoms were localized in the difference Fourier map. Images of the structures were produced with Mercury (version 2022.2.0)^5^ software. All structures have been deposited with the Cambridge Crystallographic Data Centre (CCDC) and can be accessed free of charge under the numbers 2513384-2513387.In the structure refinements of **2**‧5.16 CH_2_Cl_2_ and **1**_2_‧14 CH_2_Cl_2_: SADI and SIMU restraints were used to model occurring disorder of several *^t^*Bu groups. The PLATON/SQUEEZE^5^ extension in Olex2 was used for a solvent mask in the refinement of **2**‧5.16 CH_2_Cl_2_ (solvent mask for 10.63 CH_2_Cl_2_ per unit cell) and **1**_2_‧14 CH_2_Cl_2_ (solvent mask for 19 CH_2_Cl_2_ per unit cell).

In the structure refinement of **1**‧3.0 C_60_‧2.0 *n*-hexane‧3.5 toluene one C_60_ molecule was found to be heavily disordered about a *C*_3_ axis. This was treated by splitting the molecule over three positions and applying SIMU and DFIX restraints to allow a stable refinement. The C–C bond length between two hexagons were set to 1.401 Å and the C–C bond lengths between a hexagon and a pentagon was set to 1.458 Å, according to values obtained from gas-phase electron diffraction.^76^ The SUMP command was used to determine the respective occupancies of all three parts. Residual positive and negative electron density at the other C_60_ molecule suggested another disorder about a *C*_2_ axis. Attempts to model this disorder led to unstable refinements. Furthermore, the PLATON/SQUEEZE extension in Olex2 was used for a solvent mask in the refinement of **1** (solvent mask for 3.5 toluene per unit cell). In general, rotational disorders are well-known in solid-state structure of C_60_.^7–9^

## **1.2 Synthetic procedures and characterization data**

**1,5-bis((4-(*tert*-butyl)phenyl)ethynyl)anthracene (3)**

In a 10 mL round bottom flask 1,5-dibromoanthracene (100 mg, 298 µmol), Pd(PPh_3_)_4_ (34.4 mg, 29.8 µmol), CuI (11.3 mg, 59.5 µmol) and 1-(*tert*-butyl)-4-ethynylbenzene (161 µL, 893 µmol) were dissolved in degassed NEt_3_ (5.95 mL) and stirred at 100 °C for 16 h under inert atmosphere. The reaction mixture was evaporated on SiO_2_ and purified by flash column chromatography (10–50% CH_2_Cl_2_ in *i*-hex) to afford **3** (137 mg, 94%) as a golden solid. Elemental analysis: calcd for C_38_H_34_O_2_ (Endoperoxide species): C 87.32%, H 6.56%, O: 6.12%, found C 87.42%, H 6.46%. Melting point: M.p.: 293-294 °C.

^1^H NMR (600 MHz, CDCl_3_, 296 K): δ (ppm) = 9.00 (s, 2H; 1), 8.09 (dd, *J* = 8.5, 1.1 Hz, 2H; 2), 7.80 (dd, *J* = 6.8, 1.1 Hz, 2H; 4), 7.66 (d, *J* = 8.6 Hz, 4H; 5), 7.49 (dd, 2H, *J* = 8.6 Hz, *J* = 6.9 Hz, 2H; 3), 7.47 (d, *J* = 8.6 Hz, 4H; 6), 1.38 (s, 18H; 7).

^13^C NMR (150 MHz, CDCl_3_, 296 K): δ (ppm) = 152.0, 131.8, 131.6 (5), 131.5, 130.6 (4), 129.6 (2), 126.0 (1), 125.7 (6), 125.3 (3), 121.3, 120.5, 95.2, 87.1, 35.0, 31.4 (7).

HRMS (MALDI-TOF): calc. for C_38_H_34_^+^: 490.266 m/z [M^+^], found 490.277 m/z (error: 8.16 ppm).

**1,5-bis(4,4''-di-*tert*-butyl-4',5',6'-tris(4-(*tert*-butyl)phenyl)-[1,1':2',1''-terphenyl]-3'-yl)anthracene (4)**

Compound **3** (178 mg, 373 µmol) and 2,3,4,5-tetrakis(4-(*tert*-butyl)phenyl)cyclopenta-2,4-dien-1-one (663 mg, 1.03 mmol) were dissolved in diphenyl ether (373 µL) under inert atmosphere. The dark reaction mixture was heated in a sand-bath at 250 °C for 16 hours. Upon cooling the residue was dissolved in CH_2_Cl_2_ (1 mL) and added dropwise to MeOH (100 mL). The resulting precipitate was sonicated, filtered, washed with MeOH (100 mL), and dried in a stream of air to yield **4** (459 mg, 77%) as a tan solid. Elemental analysis: calcd for C_126_H_138_O_2_ (Endoperoxide species): C 89.84%, H 8.26%, O: 1.90%, found C 89.83%, H 7.97%. Melting point: M.p.: > 400 °C.

^1^H NMR (600 MHz, CDCl_3_, 296 K): δ (ppm) = 8.09 (s, 2H), 7.59 – 7.56 (2H), 6.96 (dd, *J* = 4.8, 0.8 Hz, 4H), 6.91 – 6.72 (24H), 6.66 (8H), 6.29 (dd, *J* = 37.8, 8.3 Hz, 8H), 1.11 (s, 18H), 1.08 (s, 18H), 0.98 (s, 18H).

^13^C NMR (150 MHz, CDCl_3_, 296 K): δ (ppm) =) δ 147.5, 147.3, 147.3, 141.3, 140.7, 140.7, 138.6, 138.3, 138.2, 137.9, 137.8, 131.3, 131.2, 131.1, 131.0, 129.7, 128.6, 127.1, 125.5, 123.4, 123.2, 123.0, 122.5, 34.2, 34.1, 34.0, 31.4, 31.3, 31.3. Low signal intensity due to low solubility, and multiple coinciding signals.

HRMS (MALDI-TOF): calc. for C_126_H_138_^+^: 1651.080 m/z [M^+^], found 1651.065 m/z (error: -9.08 ppm).

***sp*^3^-defect nanographene** (**2**)

Compound **4** (170 mg, 103 µmol) and 2,3-dichloro-5,6-dicyano-1,4-benzoquinone (420 mg, 1.85 mmol) were dissolved in dry and degassed CH_2_Cl_2_ (46.8 mL) and cooled to -42 °C with an external dry ice/acetonitrile bath. TfOH (468 µL, 103 µmol, 1 v/v%) was added dropwise. The dark reaction mixture was stirred at -42 °C for 2 h. Subsequently, the reaction was terminated by the addition of NEt_3_ (2 mL). The mixture was evaporated on SiO_2_ and purified by flash column chromatography (5–15% CH_2_Cl_2_ in *i*-hex) to afford **2** (87.1 mg, 52%) as a bright yellow solid. Elemental analysis: calcd for C_126_H_116_: C 92.83%, H 7.17%, found C 91.66%, H 6.90%. Melting point: M.p.: > 400 °C.

^1^H NMR (500 MHz, C_2_D_2_Cl_4_, 303 K): δ (ppm) =  9.28 (s, 2H; 8 or 7*), 9.27 (s, 2H; 9 or 6*), 9.07 (s, 2H; 7 or 8*), 9.03 (4H; 5 and 10), 8.97 (s, 2H; 6 or 9*), 8.89 (s, 2H; 4), 8.88 (s, 2H; 3), 8.77 (s, 2H; 11); 8.75 (d, 8.5 Hz, 2H; 2), 7.37 (d, 8.5 Hz, 2H; 1), 7.22 (s, 2H; 12), 5.68 (s, 2H; 13), 1.84 (s, 18H; 16), 1.81 (s, 18H; 15 or 17*), 1.77 (s, 18H; 17 or 15*), 1.63 (s, 18H; 14), 1.06 (s, 18H; 18). Since protons 5 and 10 have exactly the same chemical shift, the two ROESY correlations observed for this signal cannot be assigned unambiguously. The two possible assignments (with or without *) for the signals of protons 6–9 and tBu groups 15 and 17 are therefore given here.

^13^C NMR (125 MHz, C_2_D_2_Cl_4_, 303 K): δ (ppm) =  149.8, 149.7, 149.2, 142.9, 136.6, 132.5, 131.4 130.3, 130.2, 130.0, 129.8, 129.7, 129.6, 129.3, 128.9, 128.5 (1), 128.4, 128.1, 125.7, 125.4, 125.2, 124.4 (12), 123.6, 123.4, 123.3, 123.0, 122.7, 122.3, 121.3 (2), 120.2, 120.0, 119.9 (4, 6 or 9*), 119.3 (7 or 8*), 119.2 (5 or 10), 118.9, 118.3 (8 and 9 or 6* and 7*), 118.2 (11), 117.8 (3, 5 or 10), 47.2 (13), 35.5, 35.4, 35.3, 34.5, 31.8, 31.7 (14, 16), 31.6 (15, 17), 30.8 (18). The assignment of CH carbons is based on the HSQC spectrum.

HRMS (MALDI-TOF): calc. for C_126_H_116_^+^: 1628.908 *m/z* [M^+^], found 1628.910 *m/z* (error: 1.23 ppm).

**cSNG** **(1)**

In a 50 mL Schlenk flask compound **2** (50.0 mg, 30.7 µmol) and 2,3-dichloro-5,6-dicyano-1,4-benzoquinone (69.6 mg, 307 µmol) were dissolved in dry and degassed toluene (30.7 mL) and stirred at 100 °C for four days under inert atmosphere. Upon cooling to room temperature the solvent was evaporated, the residue dissolved in CH_2_Cl_2_, and flushed through a plug of SiO_2_ with 20% CH_2_Cl_2_ in *i*-hex. The solvent was evaporated and the crude product purified by recycle GPC to afford **1** (35.8 mg, 72%) as a bright orange solid. Elemental analysis: calcd for C_126_H_114_: C 92.94%, H 7.06%, found C 92.85%, H 6.30%. Melting point: M.p.: > 380 °C, decomposed.

^1^H NMR (500 MHz, toluene-d_8_, 303 K): δ (ppm) = 9.20 (4H; 6, 7), 9.18 (s, 2H; 10), 9.13 (s, 2H; 8), 9.12 (s, 2H; 11), 9.11 (s, 2H; 9), 9.02 (s, 2H; 5), 9.01 (s, 2H; 4), 8.79 (s, 2H; 3), 8.37 (d, 9.7 Hz, 2H; 2), 7.90 (s, 2H; 12), 7.64 (d, 9.7 Hz, 2H; 1), 1.69 (s, 18H; 16), 1.60 (s, 18H; 15), 1.57 (s, 18H, 14), 1.55 (s, 18H; 13), 1.53 (s, 18H; 17).

^13^C NMR (125 MHz, toluene-d_8_, 303 K): δ (ppm) =  150.3, 149.7, 149.3, 149.2, 138.6, 136.3, 134.5, 131.4, 131.2, 131.0, 130.8, 130.6, 130.5, 130.2, 130.0, 129.6, 129.5, 127.5, 125.5 (2), 124.7, 124.5 (1), 124.2, 123.8, 123.5, 123.0, 121.5, 120.9, 119.9 (9), 119.5 (4), 119.2 (3, 5), 119.0 (8), 118.9 (6 or 7), 118.6 (6 or 7), 118.2 (10), 117.7 (11), 35.6, 35.5, 35.1, 32.0, 31.9, 31.4. Several signals are masked by the intense solvent signals. The assignment of CH carbons is based on the HSQC spectrum.

HRMS (MALDI-TOF): calc. for C_126_H_114_^+^: 1626.894 *m/z* [M^+^], found 1626.892 *m/z* (error: -1.23 ppm).

# **2. X-ray crystallographic data**


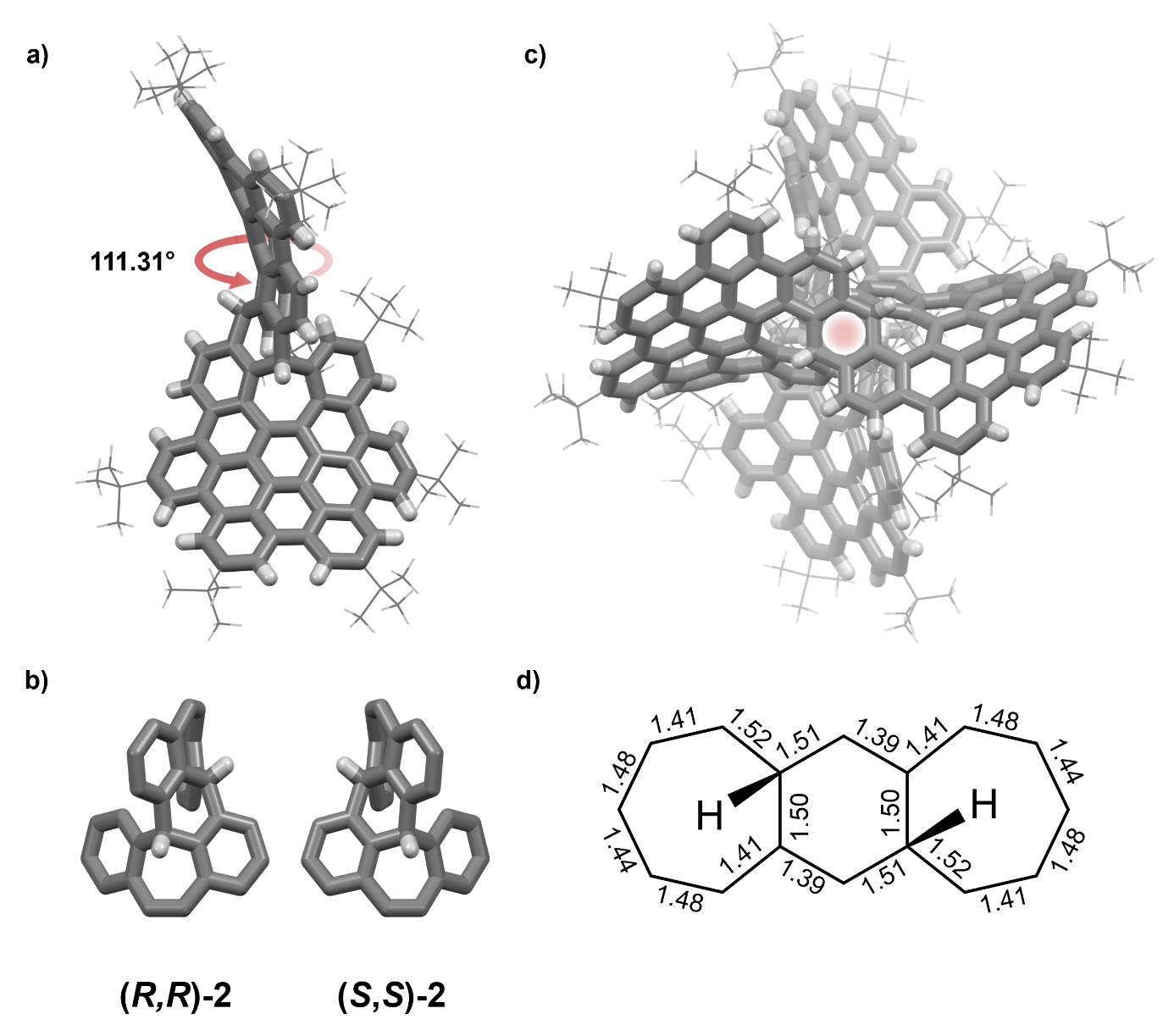


**Figure S1.** Crystal structure analysis of **2**. (a) Side view with visible twist. (b) Chiral units. (c) Solid state packing with channels through the defect six-membered ring colored in red. (d) Bond length analysis of central 7/6/7 motif.

**Table S1.** Summary of X-ray crystallographic data from single crystal of **2**.

| Identification code | FENG094 |
| --- | --- |
| Empirical formula | C_128.5_H_121_Cl_5_ |
| Formula weight | 1842.50 |
| Temperature/K | 100.00(10) |
| Crystal system | tetragonal |
| Space group | I-4 |
| a/Å | 25.99440(10) |
| b/Å | 25.99440(10) |
| c/Å | 17.8797(2) |
| α/° | 90 |
| β/° | 90 |
| γ/° | 90 |
| Volume/Å^3^ | 12081.47(16) |
| Z | 4 |
| ρ_calc_g/cm^3^ | 1.013 |
| μ/mm^‑1^ | 1.418 |
| F(000) | 3908.0 |
| Crystal size/mm^3^ | 0.347 × 0.128 × 0.1 |
| Radiation | Cu Kα (λ = 1.54184) |
| 2Θ range for data collection/° | 4.808 to 153.64 |
| Index ranges | -32 ≤ h ≤ 28, -31 ≤ k ≤ 32, -21 ≤ l ≤ 22 |
| Reflections collected | 75250 |
| Independent reflections | 12624 [R_int_ = 0.0375, R_sigma_ = 0.0222] |
| Data/restraints/parameters | 12624/168/736 |
| Goodness-of-fit on F^2^ | 1.048 |
| Final R indexes [I>=2σ (I)] | R_1_ = 0.0644, wR_2_ = 0.1843 |
| Final R indexes [all data] | R_1_ = 0.0661, wR_2_ = 0.1868 |
| Largest diff. peak/hole / e Å^-3^ | 0.56/-0.29 |
| Flack parameter | 0.07(3) |
| CCDC | 2513386 |


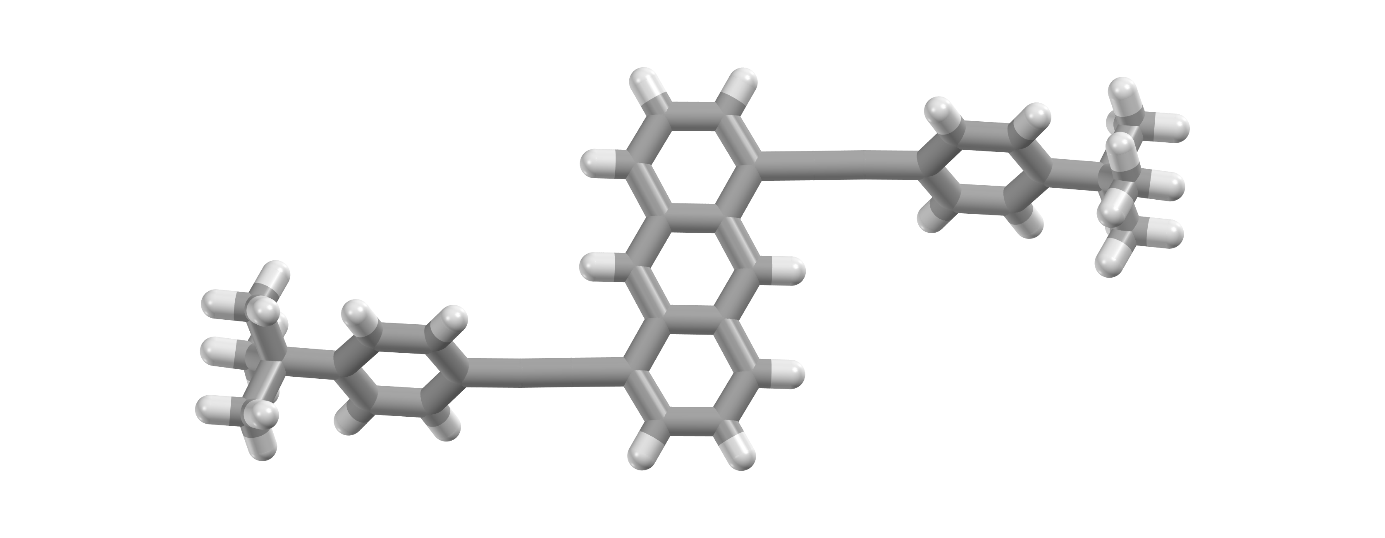


**Figure S2.** Crystal structure of **3** as capped sticks representation.

**Table S2.** Summary of X-ray crystallographic data from single crystal of **3**.

| Identification code | FENG096 |
| --- | --- |
| Empirical formula | C_38_H_34_ |
| Formula weight | 490.65 |
| Temperature/K | 100.00(10) |
| Crystal system | Triclinic |
| Space group | P-1 |
| a/Å | 6.0091(4) |
| b/Å | 7.5875(4) |
| c/Å | 15.0274(10) |
| α/° | 101.824(5) |
| β/° | 90.672(6) |
| γ/° | 97.770(5) |
| Volume/Å^3^ | 663.92(7) |
| Z | 1 |
| ρ_calc_g/cm^3^ | 1.227 |
| μ/mm^‑1^ | 0.518 |
| F(000) | 262.0 |
| Crystal size/mm^3^ | 0.357 × 0.093 × 0.037 |
| Radiation | Cu Kα (λ = 1.54184) |
| 2Θ range for data collection/° | 6.014 to 136.452 |
| Index ranges | -7 ≤ h ≤ 7, -7 ≤ k ≤ 9, -18 ≤ l ≤ 18 |
| Reflections collected | 5989 |
| Independent reflections | 2399 [R_int_ = 0.0488, R_sigma_ = 0.0511] |
| Data/restraints/parameters | 2399/0/175 |
| Goodness-of-fit on F^2^ | 1.045 |
| Final R indexes [I>=2σ (I)] | R_1_ = 0.0660, wR_2_ = 0.1763 |
| Final R indexes [all data] | R_1_ = 0.0796, wR_2_ = 0.1945 |
| Largest diff. peak/hole / e Å^-3^ | 0.39/-0.33 |
| CCDC | 2513384 |


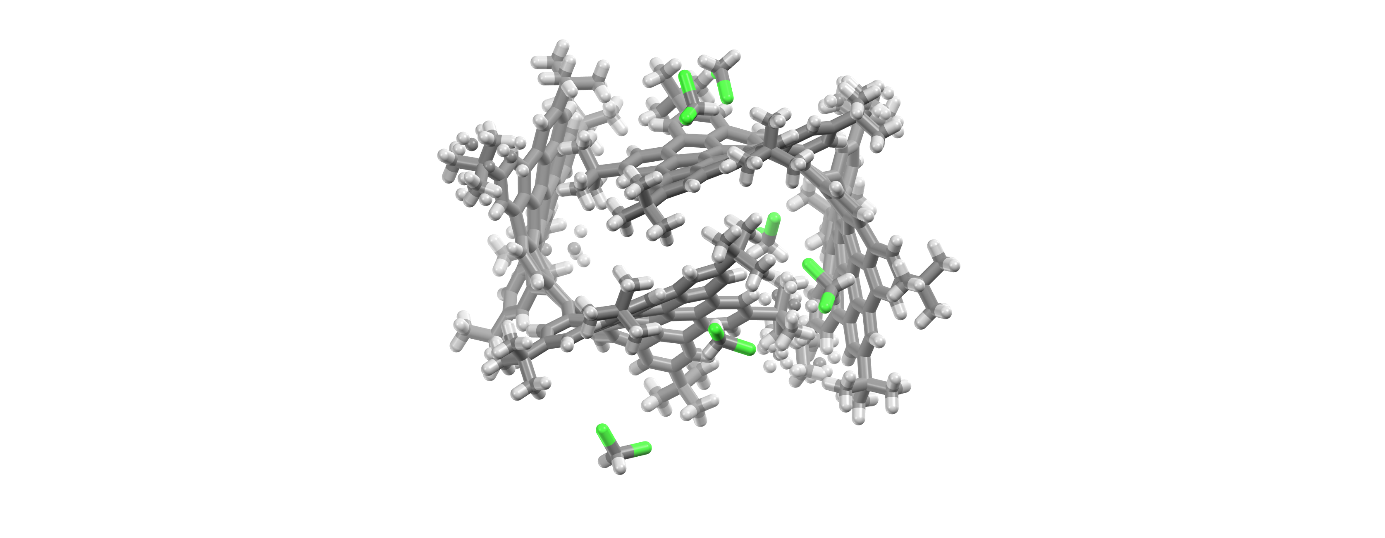


**Figure S3.** Crystal structure of **1** as capped sticks representation.

**Table S3.** Summary of X-ray crystallographic data from single crystal of **1**.

| Identification code | FENG100 |
| --- | --- |
| Empirical formula | C_128.25_H_118.5_Cl_4.5_ |
| Formula weight | 1819.25 |
| Temperature/K | 100.01(10) |
| Crystal system | Triclinic |
| Space group | P-1 |
| a/Å | 15.9254(2) |
| b/Å | 21.2192(3) |
| c/Å | 37.4457(4) |
| α/° | 105.2480(10) |
| β/° | 90.3590(10) |
| γ/° | 99.5940(10) |
| Volume/Å^3^ | 12020.9(3) |
| Z | 4 |
| ρ_calc_g/cm^3^ | 1.005 |
| μ/mm^‑1^ | 1.321 |
| F(000) | 3858.0 |
| Crystal size/mm^3^ | 0.262 × 0.178 × 0.079 |
| Radiation | Cu Kα (λ = 1.54184) |
| 2Θ range for data collection/° | 4.412 to 136.502 |
| Index ranges | -18 ≤ h ≤ 19, -25 ≤ k ≤ 16, -45 ≤ l ≤ 45 |
| Reflections collected | 123386 |
| Independent reflections | 43792 [R_int_ = 0.0582, R_sigma_ = 0.0595] |
| Data/restraints/parameters | 43792/465/2673 |
| Goodness-of-fit on F^2^ | 1.031 |
| Final R indexes [I>=2σ (I)] | R_1_ = 0.0782, wR_2_ = 0.2190 |
| Final R indexes [all data] | R_1_ = 0.1002, wR_2_ = 0.2384 |
| Largest diff. peak/hole / e Å^-3^ | 0.62/-0.60 |
| CCDC | 2513387 |


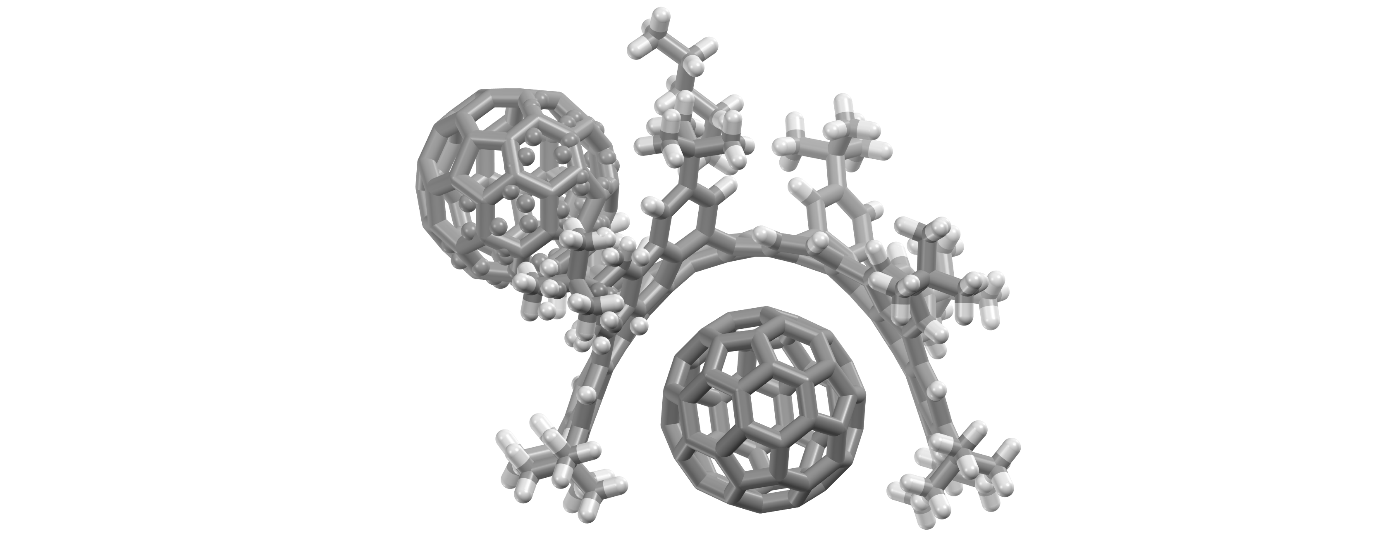


**Figure S4.** Crystal structure of **1⊃(C_60_)_3_** as capped sticks representation.

**Table S4.** Summary of X-ray crystallographic data from single crystal of **1⊃(C_60_)_3_**.

| Identification code | Feng106 |
| --- | --- |
| Empirical formula | C342.5H170 |
| Formula weight | 3962.37 |
| Temperature/K | 100.00(10) |
| Crystal system | monoclinic |
| Space group | P2/n |
| a/Å | 18.36370(10) |
| b/Å | 16.74010(10) |
| c/Å | 33.2893(2) |
| α/° | 90 |
| β/° | 94.9750(10) |
| γ/° | 90 |
| Volume/Å3 | 10194.92(10) |
| Z | 2 |
| ρcalcg/cm3 | 1.291 |
| μ/mm‑1 | 0.563 |
| F(000) | 4100.0 |
| Crystal size/mm3 | 0.222 × 0.069 × 0.065 |
| Radiation | Cu Kα (λ = 1.54184) |
| 2Θ range for data collection/° | 5.31 to 153.498 |
| Index ranges | -23 ≤ h ≤ 23, -20 ≤ k ≤ 11, -41 ≤ l ≤ 41 |
| Reflections collected | 87888 |
| Independent reflections | 21201 [Rint = 0.0331, Rsigma = 0.0259] |
| Data/restraints/parameters | 21201/2967/2017 |
| Goodness-of-fit on F2 | 1.035 |
| Final R indexes [I>=2σ (I)] | R1 = 0.0674, wR2 = 0.1863 |
| Final R indexes [all data] | R1 = 0.0795, wR2 = 0.1983 |
| Largest diff. peak/hole / e Å-3 | 0.97/-0.56 |
| CCDC | 2513385 |


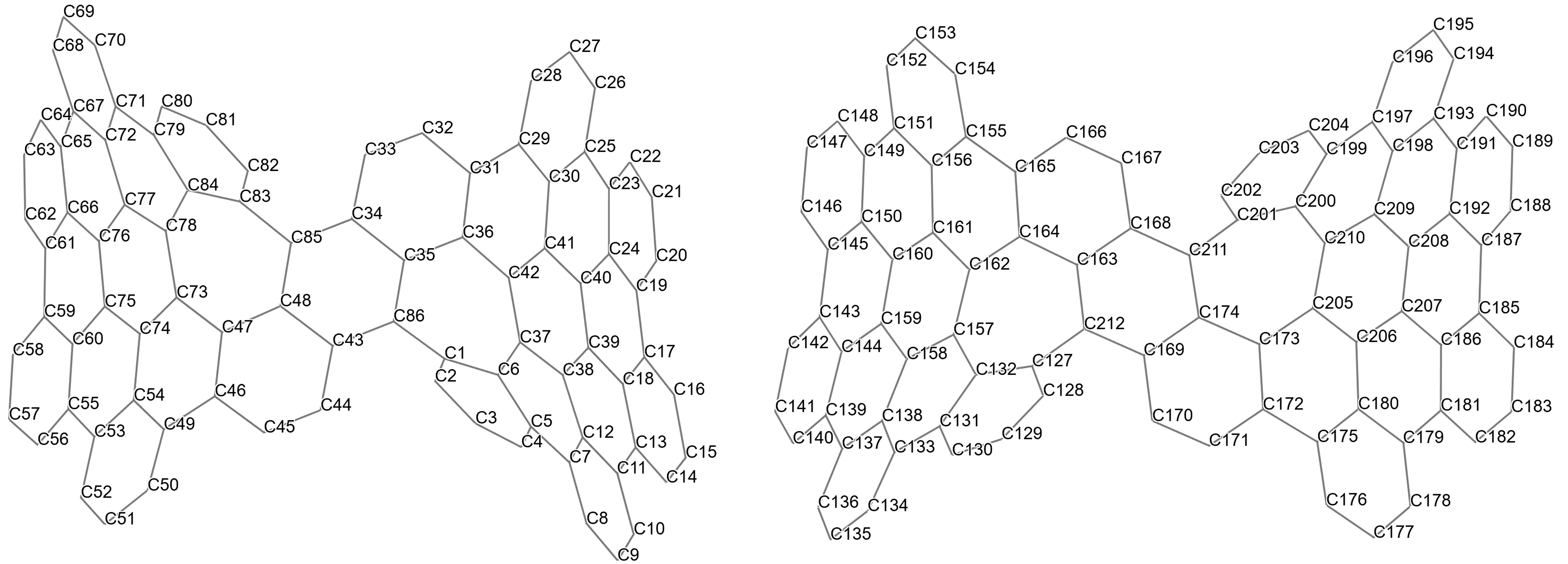


**Figure S5.** Atom labeling of form B (left) and form A (right) of compound **1**; *tert*-butyl groups and hydrogen atoms omitted.

The diameters of the saddle-shaped structures were determined as follows. For form **A** the distance between carbon atoms C_141_ and C_189_ was measured. For Form **B** the distance between C_15_ and C_63_ was determined. The angle span up by form **A** was determined by defining two planes and then measuring the angle between the two. Plane 1: C_141_, C_142_, C_143_, C_145_, C_146_, and C_147_. Plane 2: C_183_, C_184_, C_185_, C_187_, C_188_, and C_189_. The angle span up by form **B** was determined the same way. Plane 1: C_57_, C_58_, C_59_, C_61_, C_62_, and C_63_. Plane 2: C_15_, C_16_, C_17_, C_19_, C_20_, and C_21_. The depth of form **A** was established by measuring the distance of a plane to the centroid of the central benzene ring (C_163_, C_168_, C_211_, C_174_, C_169_, and C_212_) in the anthracene fragment. The plane is defined by the atoms C_141_, C_142_, C_146_, C_147_, C_183_, C_184_, C_188_, and C_189_. The same procedure was applied for form **B**. Centroid: C_34_, C_35_, C_86_, C_43_, C_48_, and C_85_. Plane: C_15_, C_16_, C_20_, C_21_, C_57_, C_58_, C_62_, and C_63_.

The nonplanarity of individual rings of form **A** and form **B** was determined as follows. For a specific ring a plane was created taking into account all ring atoms. Then, the distance of every atom to the derived plain was measured. The established values were summed up and divided by the number of ring atoms to obtain the nonplanarity value. Since both forms **A** and **B** are not perfectly *C*_2_ symmetric the average of the respective rings of both sides of the molecule is given in Table S5.


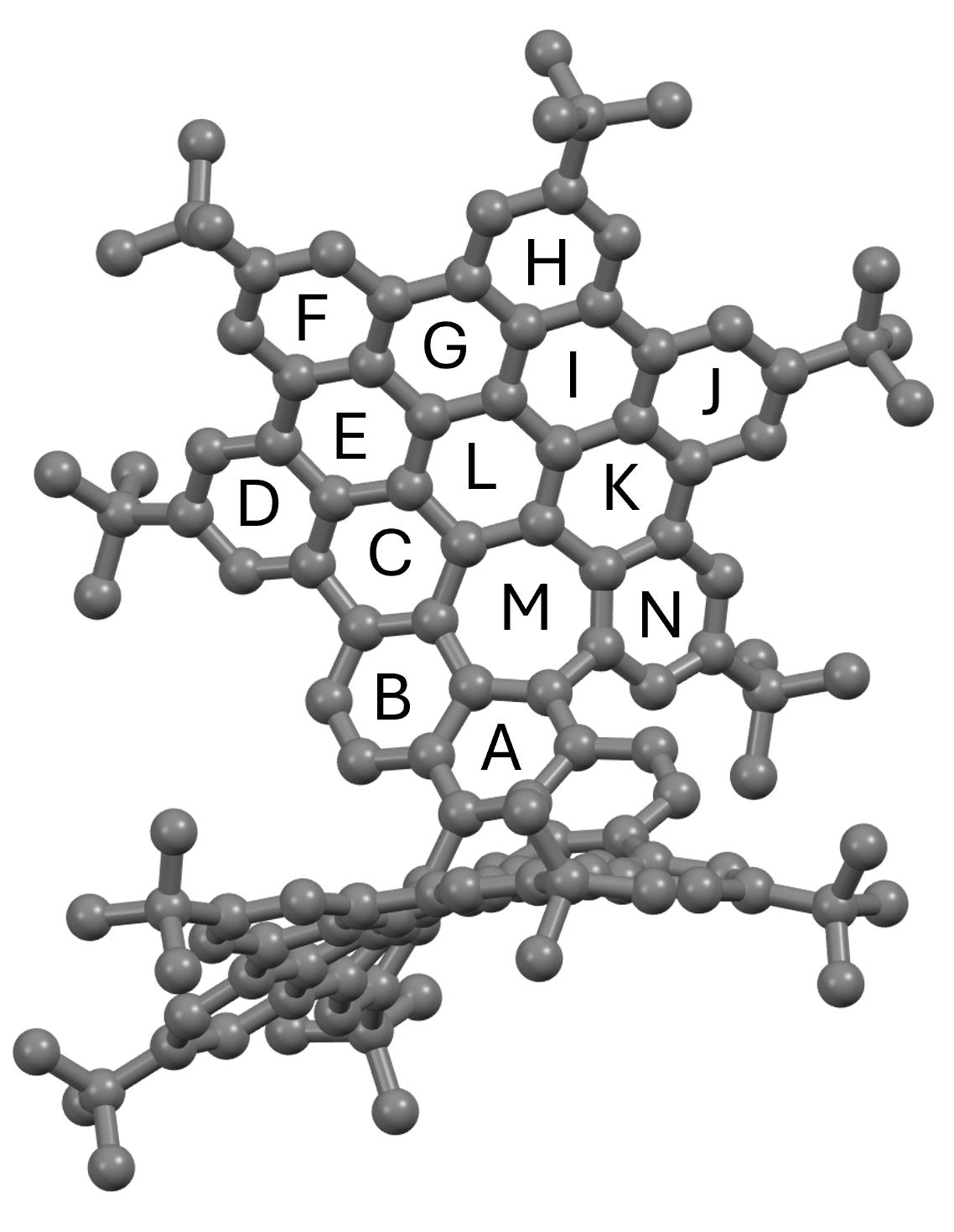


**Figure S6.** Ring numbering of **1**.

**Table S5.** Nonplanarity values for individual rings of **1**.

| **Ring** | **Form A** | **Form B** |
| --- | --- | --- |
| **A** | 0.101 | 0.114 |
| **B** | 0.045 | 0.053 |
| **C** | 0.089 | 0.091 |
| **D** | 0.028 | 0.029 |
| **E** | 0.056 | 0.056 |
| **F** | 0.015 | 0.016 |
| **G** | 0.039 | 0.042 |
| **H** | 0.026 | 0.019 |
| **I** | 0.062 | 0.056 |
| **J** | 0.007 | 0.009 |
| **K** | 0.077 | 0.078 |
| **L** | 0.051 | 0.048 |
| **M** | 0.220 | 0.227 |
| **N** | 0.060 | 0.059 |

# **3. DFT calculations**

All density functional theory (DFT) calculations were performed using the Gaussian 16 program.^10^ The B3LYP functional with Grimme’s D3 correction (Becke–Johnson damping) was used for geometry optimization in the ground state.^11^ The 6-31G(d) basis set was used. All geometry optimization was done in the gas phase and based on the single crystal structures. The fully optimized structures, **2** and **1** are obtained with no imaginary frequencies. In order to simulate the UV-Vis spectra of the molecules TD-DFT calculations were carried out by using the B3LYP functional and 6-31G(d) basis set with 30 excited states. Visualization of the geometry and isosurfaces was performed by Avogadro 1.1.1 software.^12^

For transition state (TS) calculation the structure was optimized in the gas phase with the same method and basis. A frequency analysis was conducted to verify the TS structure, confirming it as a true transition state due to the presence of a single imaginary frequency. In our case, the isomerization process represents a reaction, therefore we used Gibbs energy for estimation of the energy barrier.

A quantitative measure of aromaticity of one ring is the harmonic oscillator model of aromaticity (HOMA)^13^, which is defined with the following formula:

$$HOMA\text{=}1\text{-}\frac{c}{n}\sum_{i} \left( r_{opt}\text{-}r_{i} \right)^{2}$$

where c is an empirical constant, n is the number of bonds estimated, while r_opt_ is the optimal value of bond length. Finally, the parameter r_i_ is every individual bond length in the ring. The HOMA values in the pictures are colored as follows: blue (HOMA > 0.5) – strong aromatic character; gray (HOMA < 0.5) – weak aromatic character; red (HOMA < 0) – antiaromatic character. The HOMA calculation was performed in Multiwfn program with the standard values of the parameters.^14^

Anisotropy of the induced current density (ACID) plots were calculated by Herges’s method.^15^ Nucleus independent chemical shifts (NICS) values were calculated using the standard gauge invariant atomic orbital (GIAO) method at B3LYP functional with 6-31G(d) basis set used for the C and H atoms due to the large size of the system.^16^ The result from NICS calculations were visualized as Iso-Chemical-Shielding Surfaces (ICSS), rendered by Multiwfn 3.8 software.^17,18^

Positive areas of isosurface, positioned above and below the plane of the molecule represent a shielding effect, which indicates stronger aromaticity. Oppositely, negative values show a deshielding effect, leading to antiaromaticity – a result in contrast to that obtained via NICS calculation.^19–23^

**3.1. Optimized geometries and FMOs**


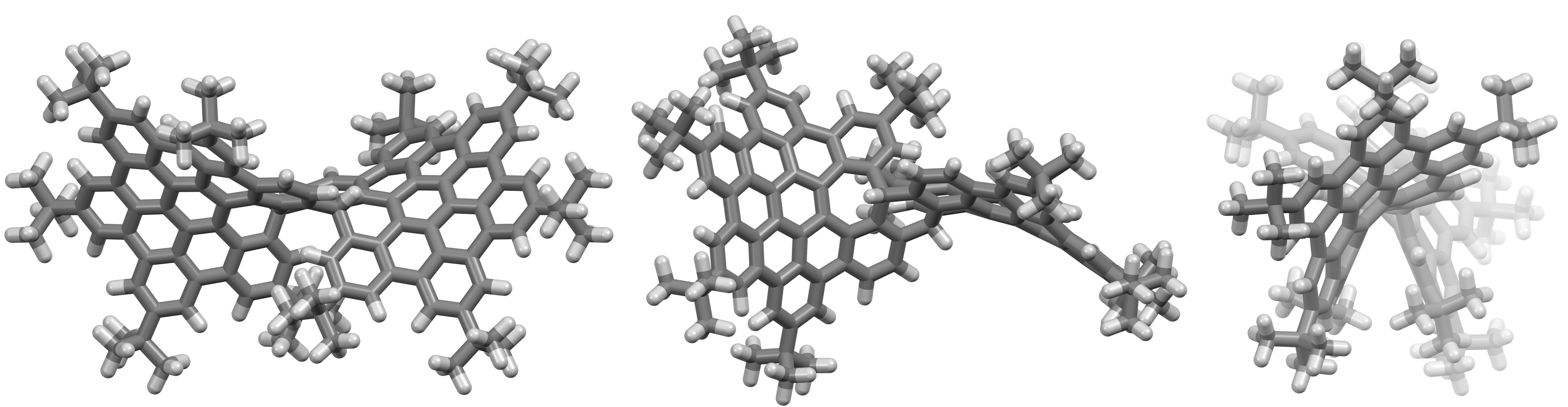


**Figure S7.** Optimized structure of **2**; left side view, middle top view, right rear view.


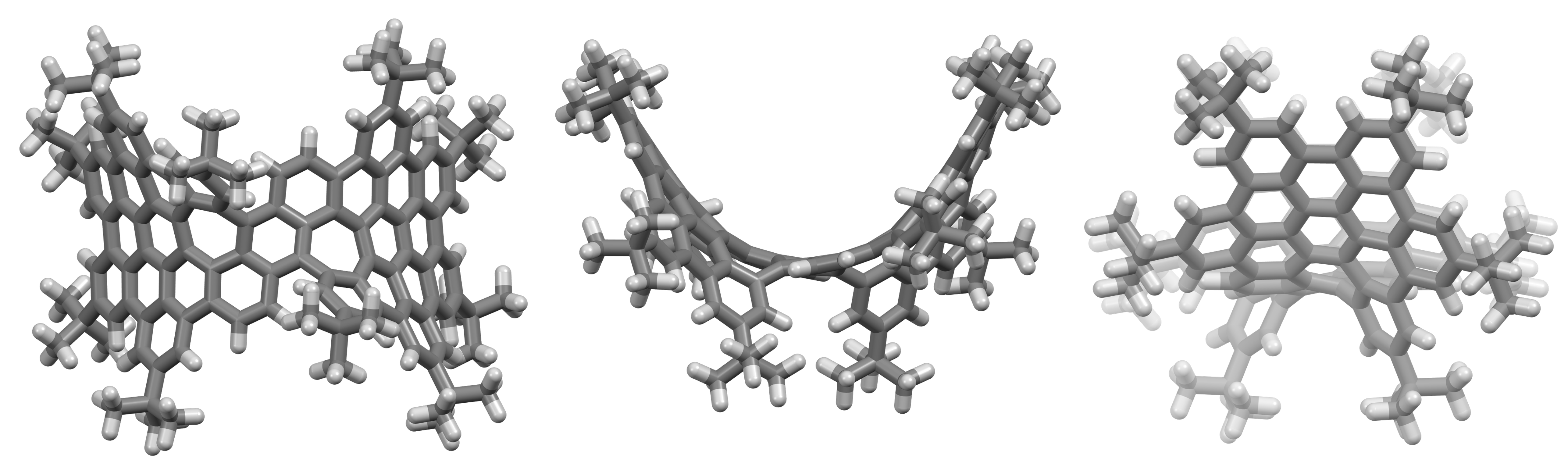


**Figure S8.** Optimized structure of **1**; top view, middle side view, right rear view.


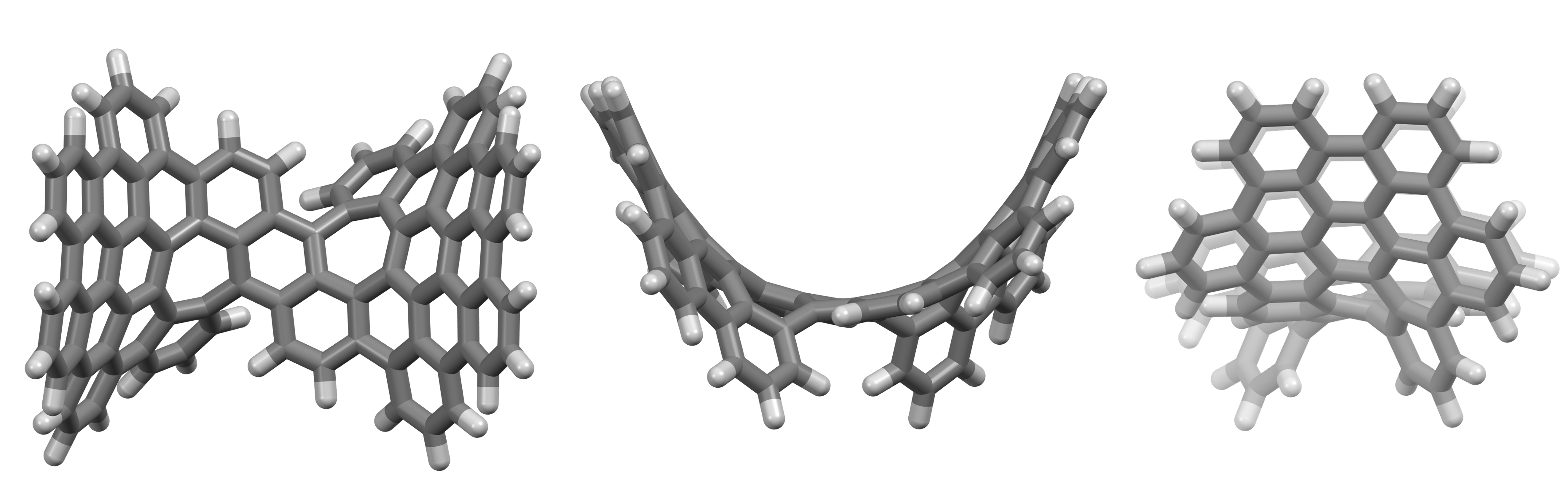


**Figure S9.** Optimized structure of **1** without tBu groups; left bottom view, middle side view, right rear view.


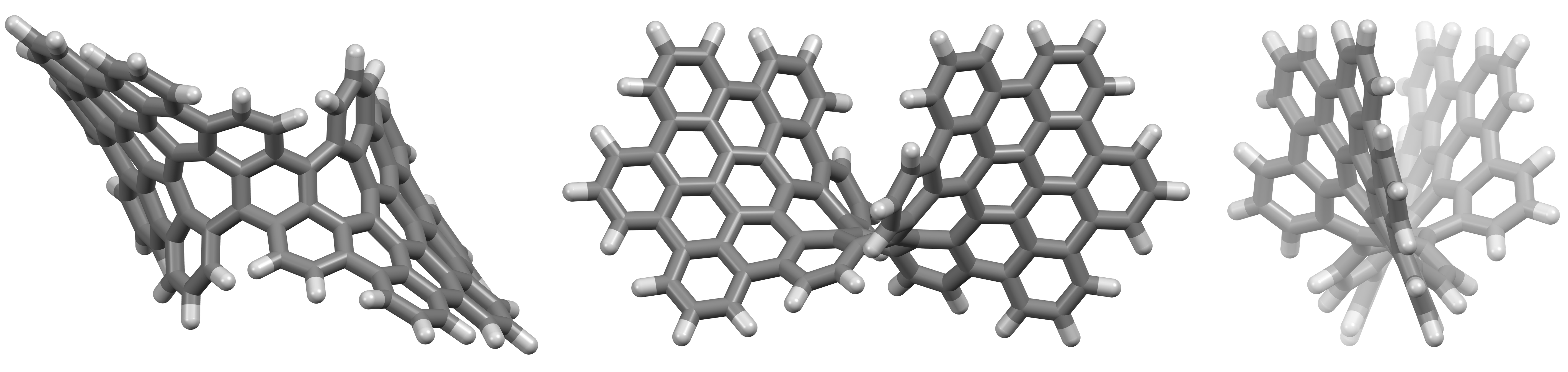


**Figure S10.** Optimized structure of TS-**1** without tBu groups; left bottom view, middle side view, right rear view.


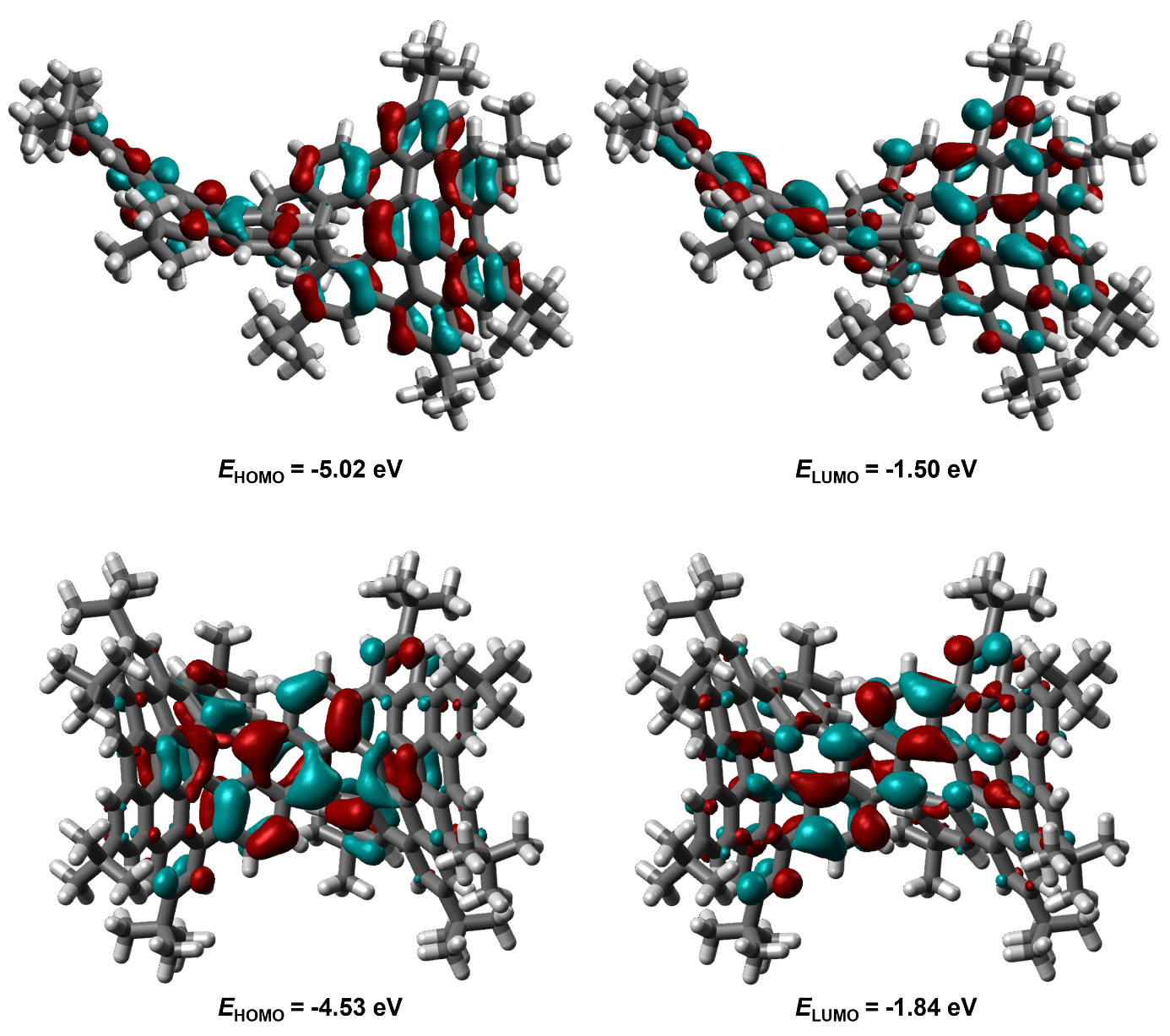


**Figure S11.** FMOs of compound **2**, isosurface values set at 0.02.

**3.2. Time-dependent DFT calculations (TD-DFT, 30 states)**

**a. Tables of transitions**

**Compound 2**

**Table** **S6.** TD-DFT calculated transitions for compound **2**.

| Transition | MOs | Energy, nm | Oscillator strength, a.u. |
| --- | --- | --- | --- |
| S0 → S1* | H-3 → L+1 (20%)  H-2 → L (23%)  H-2 → L+2 (5%)  H-1 → L+1 (6%)  H-1 → L+2 (18%)  H → L+1 (3%)  H → L+3 (21%) | 426.13 | 0.0049 |
| S0 → S2* | H-3 → L (25%)  H-2 → L+1 (15%)  H-2 → L+3 (7%)  H-1 → L+1 (10%)  H-1 → L+3 (15%)  H → L+2 (23%) | 426.03 | 0.0005 |
| S0 → S3* | H-3 → L+1 (2%)  H-3 → L+3 (15%)  H-2 → L (6%)  H-2 → L+2 (14%)  H-1 → L (28%)  H-1 → L+2 (6%)  H → L+1 (25%)  H → L+3 (2%) | 409.27 | 0.0158 |
| S0 → S4* | H-3 → L+3 (16%)  H-2 → L+2 (8%)  H-2 → L+4 (12%)  H-1 → L+2 (17%)  H-1 → L+4 (8%)  H → L (36%) | 409.24 | 0.0133 |
| S0 → S5* | H-1 → L+1 (46%)  H-1 → L+4 (4%)  H → L (44%) | 381.52 | 0.0000 |
| S0 → S6* | H-1 → L (49%)  H → L+1 (48%) | 381.22 | 0.0449 |

**Compound 1**

**Table** **S7.** TD-DFT calculated transitions for compound **1**.

| Transition | MOs | Energy, nm | Oscillator strength, a.u. |
| --- | --- | --- | --- |
| S0 → S1* | H → L (97%) | 536.10 | 0.1589 |
| S0 → S2* | H → L+1 (96%) | 481.17 | 0.0037 |
| S0 → S3* | H-3 → L+1 (2%)  H-2 → L (23%)  H-1 → L+3 (2%)  H → L+2 (63%)  H → L+5 (3%) | 469.55 | 0.0118 |
| S0 → S4* | H-3 → L (16%)  H-2 → L+1 (5%)  H-1 → L (14%)  H-1 → L+2 (3%)  H → L+3 (57%) | 461.62 | 0.0033 |
| S0 → S5* | H-3 → L (5%)  H-1 → L (82%)  H → L+3 (7%) | 455.22 | 0.0031 |
| S0 → S6* | H-2 → L (44%)  H → L+2 (18%)  H → L+4 (25%)  H → L+7 (3%) | 445.19 | 0.2612 |

**b. Simulated UV-Vis spectra (30 states)**


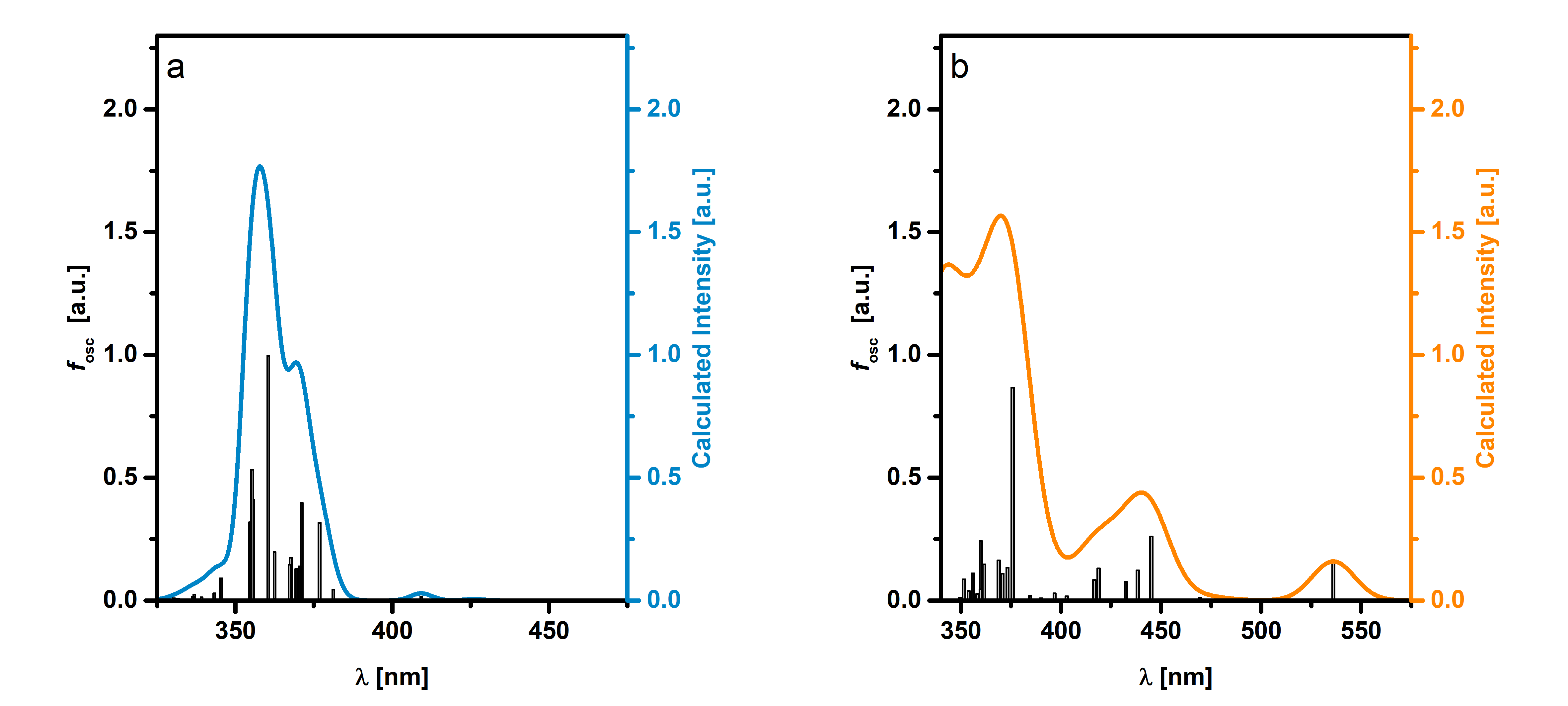


**Figure S12.** Simulated UV-Vis spectrum of **2** (a) and **1** (b).

**c. Simulated CD spectra (30 states)**


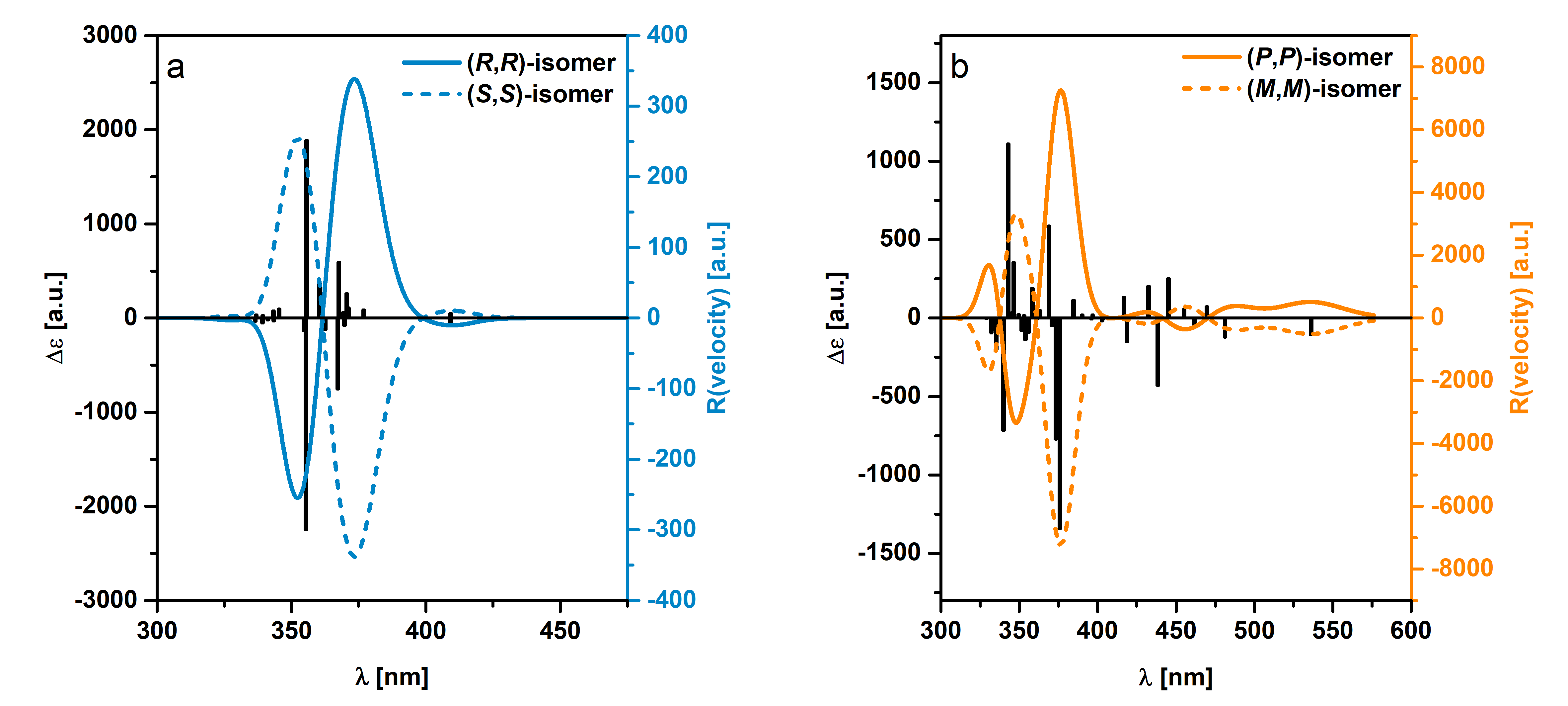


**Figure S13.** Simulated CD spectra of **2** (a) and **1** (b).

**Table S8.** Calculated states for the CD spectra a of **1** and **2**; R (velocity) in units of 10^-40^ erg esu cm G^-1^.

| **Transition** | **Wavelength, nm** | **R (velocity), 1** | **Wavelength, nm** | **R (velocity), 2** |
| --- | --- | --- | --- | --- |
| S_0_ 🡪 S_1_* | 461.62 | -1642.47213 | 426.13 | -5.2555 |
| S_0_ 🡪 S_2_* | 455.22 | -2111.86364 | 426.03 | 5.1781 |
| S_0_ 🡪 S_3_* | 445.19 | 1281.97472 | 409.27 | -61.2817 |
| S_0_ 🡪 S_4_* | 438.41 | -764.80046 | 409.24 | 50.872 |
| S_0_ 🡪 S_5_* | 432.46 | 1041.67346 | 381.52 | 0.3761 |
| S_0_ 🡪 S_6_* | 418.79 | 4605.98034 | 381.22 | 5.7269 |
| S_0_ 🡪 S_7_* | 416.67 | -8004.55184 | 376.83 | 88.5032 |
| S_0_ 🡪 S_8_* | 403.66 | 3841.45057 | 373.98 | -0.232 |
| S_0_ 🡪 S_9_* | 402.84 | -2962.78652 | 371.18 | 110.2754 |
| S_0_ 🡪 S_10_* | 396.81 | 2626.30703 | 370.57 | 260.5456 |
| S_0_ 🡪 S_11_* | 396.01 | -74.59255 | 369.77 | -81.7513 |
| S_0_ 🡪 S_12_* | 390.06 | -427.93328 | 369.37 | 60.8123 |
| S_0_ 🡪 S_13_* | 384.57 | 457.17072 | 367.61 | 597.1315 |
| S_0_ 🡪 S_14_* | 375.86 | -146.06221 | 367.28 | -756.7952 |
| S_0_ 🡪 S_15_* | 373.28 | 464.50127 | 362.51 | -125.7032 |
| S_0_ 🡪 S_16_* | 370.77 | 2414.13738 | 362.49 | 5.9943 |
| S_0_ 🡪 S_17_* | 368.90 | -29171.04508 | 360.49 | 368.8939 |
| S_0_ 🡪 S_18_* | 363.28 | -16883.17173 | 355.68 | 1888.6468 |
| S_0_ 🡪 S_19_* | 361.58 | -1103.61602 | 355.35 | -2253.8872 |
| S_0_ 🡪 S_20_* | 360.00 | 12991.02966 | 354.62 | -136.1985 |
| S_0_ 🡪 S_21_* | 359.85 | 1118.76317 | 345.40 | 101.3022 |
| S_0_ 🡪 S_22_* | 358.32 | -133.80825 | 343.43 | -35.3843 |
| S_0_ 🡪 S_23_* | 356.02 | 1275.91537 | 343.26 | 78.4295 |
| S_0_ 🡪 S_24_* | 353.90 | 1134.67184 | 341.25 | -24.9354 |
| S_0_ 🡪 S_25_* | 353.10 | 4345.22386 | 339.36 | 27.5694 |
| S_0_ 🡪 S_26_* | 351.45 | -2136.93975 | 339.18 | -53.9013 |
| S_0_ 🡪 S_27_* | 349.60 | -3194.44 | 336.84 | 36.1541 |
| S_0_ 🡪 S_28_* | 346.40 | 395.79837 | 336.47 | -38.3588 |
| S_0_ 🡪 S_29_* | 345.51 | -1917.09447 | 331.64 | -6.3808 |
| S_0_ 🡪 S_30_* | 343.01 | 560.86571 | 330.37 | 6.8479 |


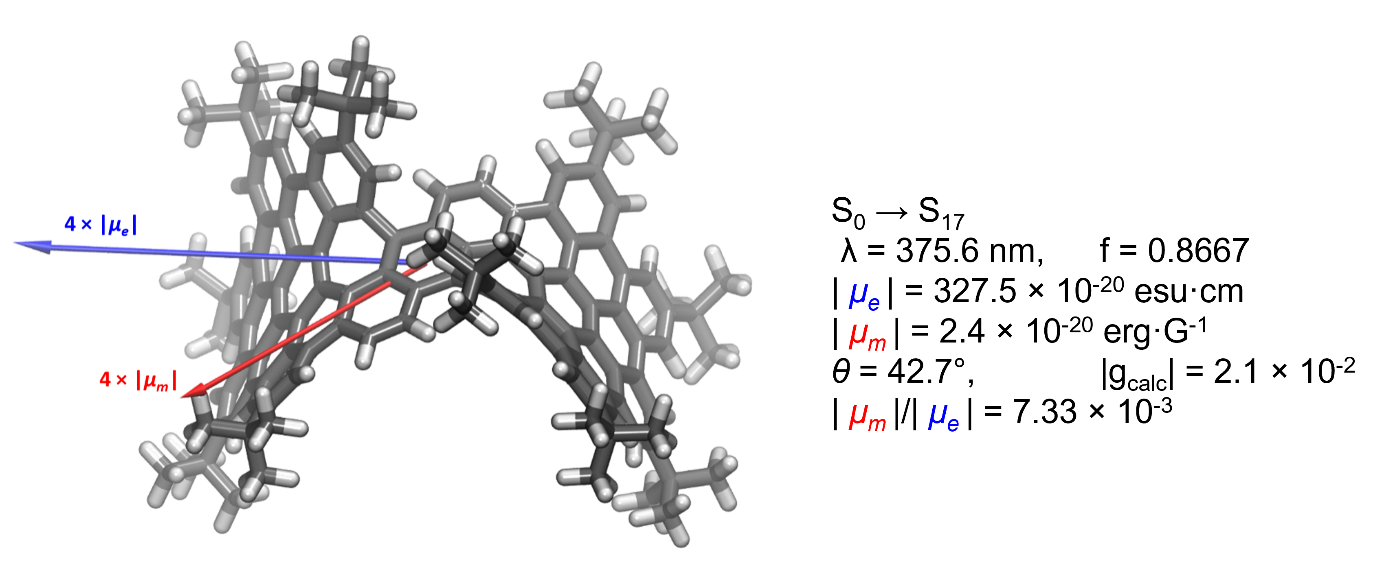


**Figure S14.** Visualization of calculated transition dipole moments of **1**.


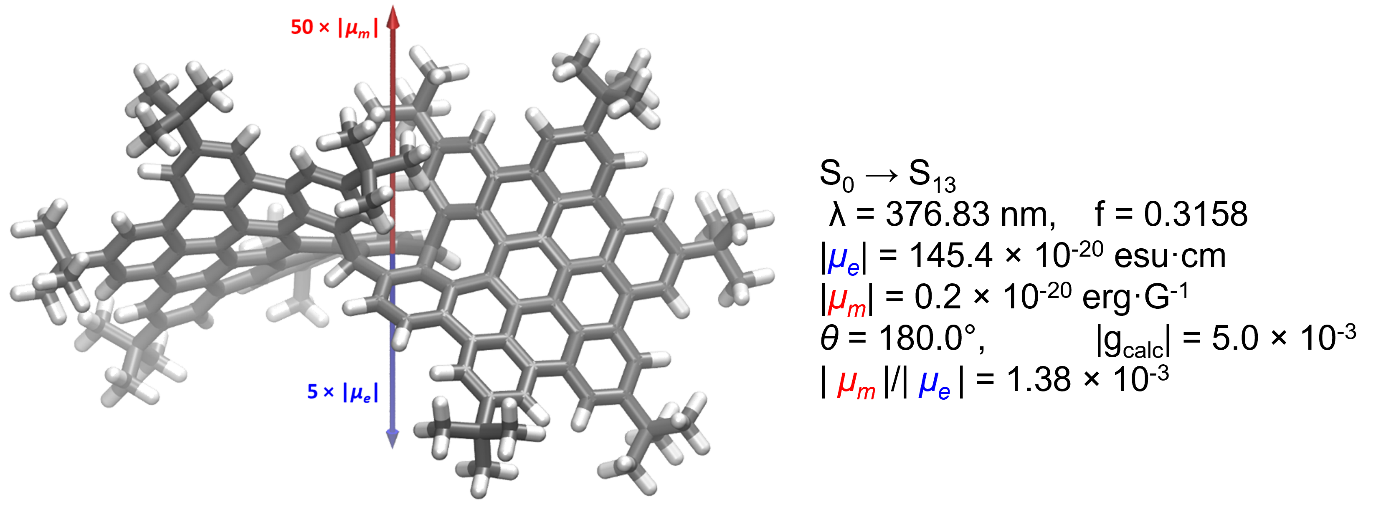


**Figure S15.** Visualization of calculated transition dipole moments of **2**.

**3.3 Aromaticity calculation**


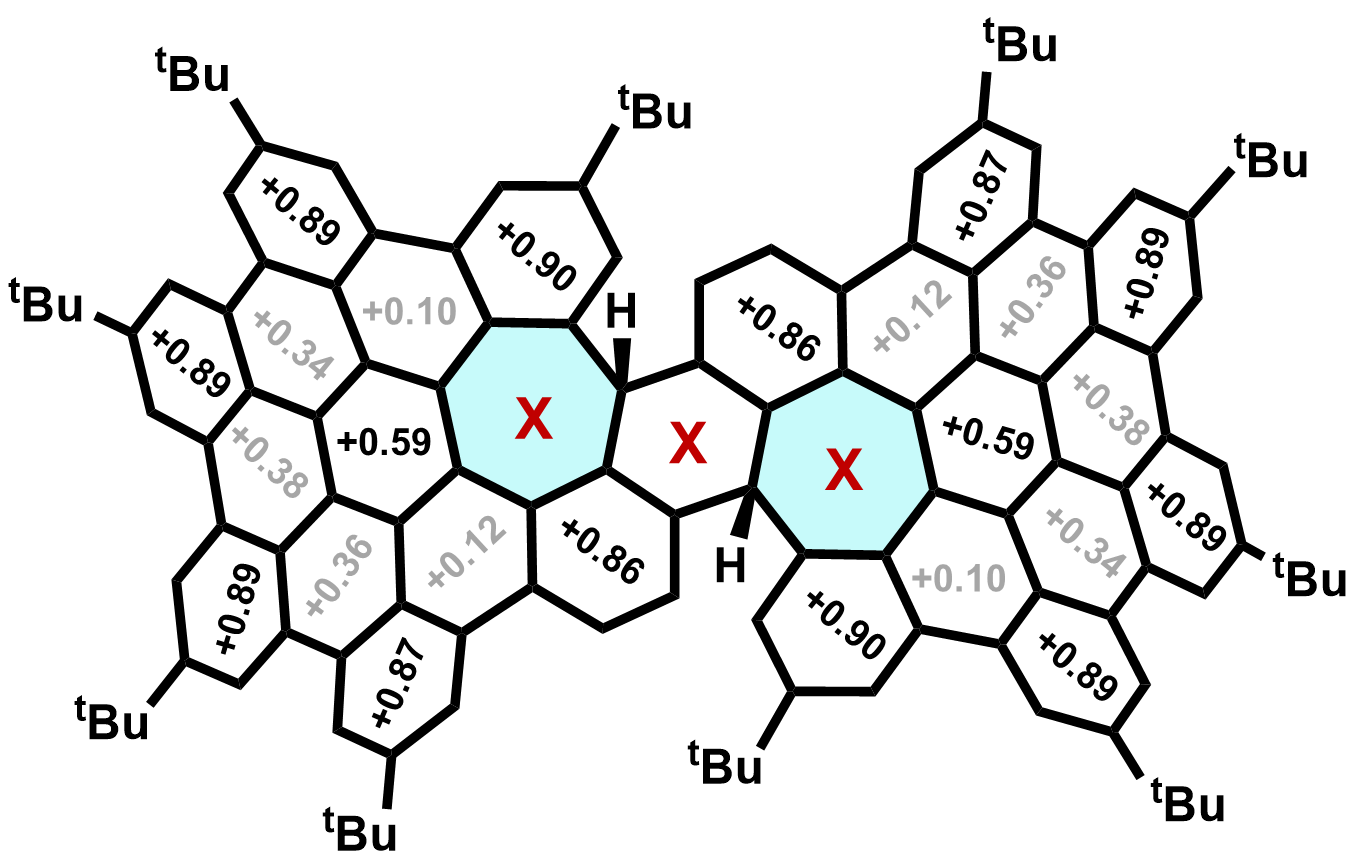


**Figure S16.** HOMA calculation of **2**. Rings marked with “X” are considered non-aromatic.

**Tables with Cartesian coordinates**

**Table S9.** Cartesian coordinates of optimized structures of **1** (left) and **2** (right).

| **Compound 1** |  |  |  | **Compound 2** |  |  |  |
| --- | --- | --- | --- | --- | --- | --- | --- |
| **atom** | **x** | **y** | **z** | **atom** | **x** | **y** | **z** |
| C | -3.4053 | 4.6189 | 6.0849 | C | 5.7067 | -3.3444 | 7.3229 |
| C | -1.9572 | 2.7239 | 6.8369 | C | 3.2117 | -3.4835 | 7.3024 |
| C | -4.4431 | 2.5159 | 7.0176 | C | 4.5506 | -5.3847 | 6.3873 |
| C | -3.2908 | 3.0773 | 6.1512 | C | 4.4854 | -3.844 | 6.5144 |
| C | 4.4556 | -2.4678 | 7.024 | C | 9.3964 | -6.477 | 2.8777 |
| C | 1.9695 | -2.6774 | 6.8477 | C | 11.2672 | -4.9785 | 2.1712 |
| C | 3.4171 | -4.5761 | 6.104 | C | 9.9748 | -6.3029 | 0.4532 |
| C | 3.3023 | -3.0343 | 6.1622 | C | 9.8678 | -5.508 | 1.7765 |
| C | 8.1168 | -6.5097 | 0.4347 | C | 12.4625 | 1.1262 | -2.4145 |
| C | 9.8568 | -4.9919 | 1.4562 | C | 12.4471 | -1.3625 | -2.6362 |
| C | 8.0732 | -6.0072 | 2.8819 | C | 11.3104 | 0.0276 | -4.3742 |
| C | 8.367 | -5.409 | 1.493 | C | 11.6124 | -0.0873 | -2.8609 |
| C | 7.9416 | -3.4847 | -5.5411 | C | 8.1766 | 5.9124 | -3.6738 |
| C | 8.9785 | -1.3331 | -6.2752 | C | 6.5014 | 7.0806 | -2.2336 |
| C | 10.1548 | -2.7418 | -4.5793 | C | 8.6531 | 6.306 | -1.2259 |
| C | 8.7658 | -2.2642 | -5.0664 | C | 7.5542 | 5.9562 | -2.2578 |
| C | 6.0781 | 5.6619 | -5.7165 | C | 1.0504 | 5.9231 | -2.8397 |
| C | 8.1125 | 4.2628 | -6.1038 | C | 0.1264 | 3.9315 | -4.0484 |
| C | 8.1872 | 6.0832 | -4.394 | C | -0.8889 | 4.6826 | -1.8713 |
| C | 7.2896 | 4.992 | -5.0248 | C | 0.4361 | 4.5224 | -2.6502 |
| C | 3.6287 | 8.4913 | -0.3039 | C | -5.7067 | -3.3446 | -7.3228 |
| C | 3.1482 | 8.0841 | 2.1147 | C | -3.2117 | -3.4832 | -7.3025 |
| C | 5.5158 | 8.0506 | 1.3147 | C | -4.5502 | -5.3847 | -6.3873 |
| C | 4.0606 | 7.6801 | 0.941 | C | -4.4852 | -3.844 | -6.5144 |
| C | -5.5136 | -8.0339 | 1.3748 | C | -9.3954 | -6.478 | -2.8768 |
| C | -3.144 | -8.0629 | 2.169 | C | -11.2666 | -4.9801 | -2.1703 |
| C | -3.6307 | -8.485 | -0.2458 | C | -9.9734 | -6.3037 | -0.4523 |
| C | -4.0592 | -7.666 | 0.9951 | C | -9.8669 | -5.509 | -1.7757 |
| C | -8.1998 | -6.1022 | -4.3384 | C | -1.0505 | 5.9228 | 2.8404 |
| C | -8.1287 | -4.2927 | -6.0598 | C | -0.1269 | 3.931 | 4.0493 |
| C | -6.094 | -5.6904 | -5.6693 | C | 0.8889 | 4.6821 | 1.8723 |
| C | -7.3032 | -5.0156 | -4.9785 | C | -0.4363 | 4.522 | 2.651 |
| C | -9.1519 | 3.6497 | -4.867 | C | -8.1763 | 5.9123 | 3.6737 |
| C | -10.087 | 1.3852 | -5.3587 | C | -6.5015 | 7.0806 | 2.2331 |
| C | -7.8777 | 2.0968 | -6.353 | C | -8.6534 | 6.306 | 1.226 |
| C | -8.7804 | 2.1725 | -5.0984 | C | -7.5543 | 5.9562 | 2.2576 |
| C | -9.8465 | 5.0188 | 1.4405 | C | -12.463 | 1.1267 | 2.4126 |
| C | -8.0582 | 6.0395 | 2.8564 | C | -12.448 | -1.362 | 2.6345 |
| C | -8.1043 | 6.5252 | 0.4057 | C | -11.3117 | 0.0281 | 4.3728 |
| C | -8.3556 | 5.4325 | 1.4721 | C | -11.6132 | -0.0869 | 2.8594 |
| C | 0.1955 | -2.7957 | 2.2608 | C | -3.6387 | 2.8829 | 1.1937 |
| C | -0.9075 | -3.5611 | 2.0509 | C | -2.7416 | 3.8205 | 1.7465 |
| C | -2.1577 | -2.9954 | 1.6489 | C | -1.3907 | 3.5597 | 1.9207 |
| C | -2.3343 | -1.5993 | 1.6088 | C | -0.9478 | 2.287 | 1.5293 |
| C | 5.7533 | -2.5583 | 1.7845 | C | -1.7951 | 1.3389 | 0.9708 |
| C | 6.5445 | -3.6825 | 2.0764 | C | -3.1636 | 1.6241 | 0.7233 |
| C | 7.4863 | -4.1847 | 1.1804 | C | 3.6386 | 2.8832 | -1.1934 |
| C | 7.6173 | -3.5293 | -0.0498 | C | 2.7415 | 3.8208 | -1.7459 |
| C | 6.8523 | -2.4106 | -0.3919 | C | 1.3906 | 3.56 | -1.92 |
| C | 7.0462 | -1.7164 | -1.6666 | C | 0.9477 | 2.2873 | -1.5289 |
| C | 7.7509 | -2.28 | -2.7327 | C | 1.795 | 1.3391 | -0.9706 |
| C | 8.0252 | -1.5683 | -3.9088 | C | 3.1636 | 1.6242 | -0.7231 |
| C | 7.6187 | -0.2355 | -3.9654 | C | 6.8649 | -3.0875 | 2.1414 |
| C | 6.9043 | 0.3797 | -2.9226 | C | 7.7487 | -4.1549 | 2.3517 |
| C | 6.5643 | 1.8027 | -2.9362 | C | 8.9035 | -4.322 | 1.5843 |
| C | 7.0482 | 2.6709 | -3.9301 | C | 9.1837 | -3.3496 | 0.6178 |
| C | 6.7848 | 4.04 | -3.9241 | C | 8.3332 | -2.2657 | 0.3641 |
| C | 6.0324 | 4.5481 | -2.8578 | C | 7.1147 | -2.1706 | 1.0923 |
| C | 5.5385 | 3.7335 | -1.8356 | C | 8.681 | -1.1933 | -0.5715 |
| C | 4.7775 | 4.2728 | -0.7096 | C | 9.9074 | -1.167 | -1.2569 |
| C | 4.7495 | 5.6392 | -0.4172 | C | 10.2863 | -0.1043 | -2.0773 |
| C | 3.9826 | 6.1746 | 0.6279 | C | 9.4134 | 0.9877 | -2.1653 |
| C | 3.1601 | 5.2963 | 1.3304 | C | 8.1852 | 1.0157 | -1.4998 |
| C | 3.1474 | 3.9161 | 1.0621 | C | 7.7773 | -0.1118 | -0.7423 |
| C | 5.7686 | 2.3338 | -1.8857 | C | 7.3048 | 2.1833 | -1.5296 |
| C | 6.5423 | -0.3954 | -1.7906 | C | 7.7757 | 3.4458 | -1.9012 |
| C | 5.9077 | -1.9009 | 0.5422 | C | 6.9537 | 4.5797 | -1.9172 |
| C | 4.0372 | 3.3759 | 0.1103 | C | 5.6032 | 4.3945 | -1.6239 |
| C | 5.1796 | -0.6785 | 0.2733 | C | 5.074 | 3.1374 | -1.2858 |
| C | 5.6341 | 0.1305 | -0.803 | C | 5.9447 | 2.0332 | -1.1411 |
| C | 5.2035 | 1.465 | -0.8801 | C | 3.5216 | -1.8151 | 3.3522 |
| C | 4.2316 | 1.9486 | 0.0421 | C | 3.4798 | -2.4047 | 4.6271 |
| C | -3.1453 | -3.9015 | 1.0907 | C | 4.509 | -3.2111 | 5.1105 |
| C | -3.1577 | -5.2799 | 1.3677 | C | 5.5965 | -3.4446 | 4.2603 |
| C | -3.9818 | -6.1626 | 0.6725 | C | 5.7011 | -2.857 | 2.9962 |
| C | -4.7509 | -5.6335 | -0.3741 | C | 4.6734 | -1.9809 | 2.5477 |
| C | -4.7794 | -4.2689 | -0.6749 | C | 4.8424 | -1.1861 | 1.351 |
| C | -5.5431 | -3.7365 | -1.8022 | C | 6.1308 | -1.1725 | 0.7508 |
| C | -6.0399 | -4.5578 | -2.818 | C | 6.4533 | -0.1594 | -0.1686 |
| C | -6.7951 | -4.0568 | -3.8852 | C | 5.4791 | 0.8202 | -0.5038 |
| C | -7.0584 | -2.6874 | -3.8988 | C | 4.1089 | 0.652 | -0.1314 |
| C | -6.5714 | -1.8136 | -2.9124 | C | -8.6811 | -1.1934 | 0.5712 |
| C | -6.9118 | -0.3894 | -2.9076 | C | -9.9078 | -1.1668 | 1.2561 |
| C | -7.6283 | 0.2199 | -3.9456 | C | -10.2869 | -0.1041 | 2.0763 |
| C | -8.0383 | 1.5568 | -3.8969 | C | -9.4138 | 0.9878 | 2.1646 |
| C | -7.7616 | 2.2747 | -2.7309 | C | -8.1855 | 1.0157 | 1.4993 |
| C | -7.0501 | 1.713 | -1.662 | C | -7.7774 | -0.1119 | 0.742 |
| C | -6.8516 | 2.4166 | -0.3942 | C | -7.305 | 2.1833 | 1.5293 |
| C | -7.6132 | 3.5403 | -0.0585 | C | -7.7759 | 3.4458 | 1.9008 |
| C | -7.4785 | 4.2038 | 1.1666 | C | -6.9538 | 4.5797 | 1.917 |
| C | -6.536 | 3.7059 | 2.0647 | C | -5.6033 | 4.3944 | 1.6239 |
| C | -5.7477 | 2.5784 | 1.7793 | C | -5.0741 | 3.1373 | 1.2859 |
| C | -6.5466 | 0.3944 | -1.779 | C | -5.9448 | 2.0331 | 1.141 |
| C | -5.773 | -2.3373 | -1.8608 | C | -6.8646 | -3.0879 | -2.1413 |
| C | -4.0372 | -3.367 | 0.1376 | C | -7.7483 | -4.1554 | -2.3514 |
| C | -5.9057 | 1.9124 | 0.5419 | C | -8.903 | -4.3226 | -1.5839 |
| C | -4.2315 | -1.9399 | 0.0609 | C | -9.1833 | -3.3501 | -0.6175 |
| C | -5.205 | -1.4617 | -0.8617 | C | -8.333 | -2.266 | -0.3641 |
| C | -5.6356 | -0.1266 | -0.7911 | C | -7.1145 | -2.1709 | -1.0923 |
| C | -5.1791 | 0.6881 | 0.2796 | C | -3.5215 | -1.8151 | -3.3522 |
| C | 2.5024 | -1.1322 | 2.8786 | C | -3.4797 | -2.4046 | -4.6271 |
| C | 2.364 | -1.7319 | 4.1352 | C | -4.5088 | -3.2112 | -5.1105 |
| C | 3.401 | -2.4501 | 4.7422 | C | -5.5963 | -3.4448 | -4.2602 |
| C | 4.5307 | -2.672 | 3.9597 | C | -5.7009 | -2.8572 | -2.9961 |
| C | 4.6949 | -2.105 | 2.6837 | C | -4.6733 | -1.981 | -2.5477 |
| C | 3.743 | -1.1569 | 2.2067 | C | -4.8423 | -1.1862 | -1.3509 |
| C | 4.0906 | -0.267 | 1.0922 | C | -6.1307 | -1.1727 | -0.7509 |
| C | 3.5417 | 1.0606 | 0.9319 | C | -6.4533 | -0.1595 | 0.1686 |
| C | 1.2742 | -0.5651 | 2.2832 | C | -5.4792 | 0.82 | 0.5038 |
| C | 0.1134 | -1.3743 | 2.1869 | C | -4.1089 | 0.6519 | 0.1315 |
| C | -1.1916 | -0.7854 | 2.0021 | C | 3.7854 | -0.3748 | 0.8361 |
| C | -1.2692 | 0.5863 | 2.2827 | C | -3.7854 | -0.3749 | -0.8361 |
| C | 0.9121 | 3.581 | 2.0299 | C | -0.033 | -0.7644 | 2.767 |
| C | 2.1613 | 3.0132 | 1.628 | C | 1.119 | -1.1617 | 3.4114 |
| C | 2.3379 | 1.6169 | 1.5953 | C | 2.3708 | -1.1355 | 2.7647 |
| C | 1.196 | 0.8051 | 1.9954 | C | 2.4592 | -0.5841 | 1.4533 |
| C | -0.1086 | 1.395 | 2.1798 | C | -1.2928 | -0.0732 | 0.7128 |
| C | -0.1905 | 2.8168 | 2.2463 | C | 0.0095 | -0.3925 | 1.4171 |
| C | -3.5397 | -1.0467 | 0.9449 | C | 1.2422 | -0.3246 | 0.7715 |
| C | -4.088 | 0.2811 | 1.0989 | C | 1.2928 | -0.0731 | -0.7127 |
| C | -3.738 | 1.1778 | 2.2072 | C | -2.4592 | -0.5841 | -1.4532 |
| C | -4.6885 | 2.1293 | 2.6798 | C | -1.2422 | -0.3246 | -0.7715 |
| C | -4.5223 | 2.7036 | 3.9524 | C | -0.0095 | -0.3924 | -1.4171 |
| C | -3.3917 | 2.4855 | 4.7346 | C | 0.033 | -0.7642 | -2.767 |
| C | -2.3557 | 1.7635 | 4.1302 | C | -1.1189 | -1.1615 | -3.4114 |
| C | -2.4962 | 1.1567 | 2.8772 | C | -2.3707 | -1.1354 | -2.7646 |
| H | -2.5993 | 5.0462 | 5.4773 | H | 5.7092 | -3.7913 | 8.3247 |
| H | -4.3578 | 4.9398 | 5.6501 | H | 6.6529 | -3.6075 | 6.8385 |
| H | -3.3372 | 5.0501 | 7.0913 | H | 5.682 | -2.2547 | 7.4364 |
| H | -1.8183 | 1.6401 | 6.9205 | H | 2.3053 | -3.8405 | 6.8002 |
| H | -1.0973 | 3.1379 | 6.2983 | H | 3.1175 | -2.4021 | 7.454 |
| H | -1.9419 | 3.1399 | 7.8506 | H | 3.246 | -3.953 | 8.292 |
| H | -5.4243 | 2.764 | 6.5993 | H | 3.6923 | -5.7682 | 5.824 |
| H | -4.3798 | 1.4245 | 7.094 | H | 4.5414 | -5.85 | 7.3806 |
| H | -4.3963 | 2.9318 | 8.0317 | H | 5.4603 | -5.7144 | 5.8745 |
| H | 5.4363 | -2.718 | 6.6057 | H | 10.1093 | -7.3041 | 2.97 |
| H | 4.3921 | -1.3761 | 7.0946 | H | 9.3345 | -5.9857 | 3.8556 |
| H | 4.4103 | -2.8782 | 8.0404 | H | 8.4162 | -6.9101 | 2.6483 |
| H | 1.8304 | -1.5933 | 6.9257 | H | 11.9661 | -5.8131 | 2.3068 |
| H | 1.109 | -3.0946 | 6.3125 | H | 11.6852 | -4.3164 | 1.4057 |
| H | 1.9557 | -3.088 | 7.8638 | H | 11.2228 | -4.4158 | 3.1106 |
| H | 2.6104 | -5.0069 | 5.4999 | H | 10.6635 | -7.1486 | 0.5708 |
| H | 4.3691 | -4.8992 | 5.6697 | H | 10.3476 | -5.6815 | -0.3676 |
| H | 3.3505 | -5.0019 | 7.1128 | H | 8.9976 | -6.699 | 0.1542 |
| H | 7.0687 | -6.83 | 0.4425 | H | 13.4088 | 1.1535 | -2.9687 |
| H | 8.3521 | -6.1643 | -0.5774 | H | 12.696 | 1.0691 | -1.3452 |
| H | 8.7425 | -7.3868 | 0.641 | H | 11.9459 | 2.075 | -2.5929 |
| H | 10.1463 | -4.5952 | 0.4775 | H | 11.9115 | -2.263 | -2.9579 |
| H | 10.0654 | -4.2185 | 2.2041 | H | 12.7286 | -1.487 | -1.5842 |
| H | 10.4997 | -5.8547 | 1.6696 | H | 13.373 | -1.3038 | -3.2193 |
| H | 8.2608 | -5.2856 | 3.6852 | H | 12.2445 | 0.0498 | -4.9489 |
| H | 7.0369 | -6.3541 | 2.9666 | H | 10.7175 | -0.8264 | -4.7211 |
| H | 8.7247 | -6.8709 | 3.0562 | H | 10.7517 | 0.939 | -4.6111 |
| H | 7.7882 | -4.2121 | -4.7372 | H | 7.4183 | 5.6733 | -4.4281 |
| H | 6.9546 | -3.1742 | -5.9022 | H | 8.6146 | 6.8854 | -3.9279 |
| H | 8.4584 | -3.9981 | -6.3613 | H | 8.97 | 5.1614 | -3.7487 |
| H | 8.0279 | -0.9762 | -6.6876 | H | 5.706 | 6.9118 | -2.9685 |
| H | 9.5903 | -0.461 | -6.0173 | H | 6.978 | 8.0366 | -2.478 |
| H | 9.5 | -1.8766 | -7.0711 | H | 6.039 | 7.1858 | -1.2456 |
| H | 10.764 | -1.8956 | -4.2415 | H | 8.2378 | 6.3532 | -0.2128 |
| H | 10.0753 | -3.4488 | -3.747 | H | 9.0974 | 7.2818 | -1.4578 |
| H | 10.6919 | -3.2449 | -5.3928 | H | 9.4595 | 5.5653 | -1.2209 |
| H | 5.4291 | 4.9117 | -6.1824 | H | 1.3404 | 6.3713 | -1.8821 |
| H | 5.4696 | 6.2355 | -5.0096 | H | 0.3167 | 6.5871 | -3.3106 |
| H | 6.4188 | 6.3509 | -6.4992 | H | 1.9328 | 5.9029 | -3.4892 |
| H | 9.0084 | 3.7904 | -5.6848 | H | -0.5526 | 4.5926 | -4.6014 |
| H | 7.5244 | 3.492 | -6.6146 | H | -0.3492 | 2.9479 | -3.9717 |
| H | 8.4435 | 4.9813 | -6.8622 | H | 1.044 | 3.8153 | -4.6366 |
| H | 7.6477 | 6.6753 | -3.6475 | H | -0.7213 | 5.129 | -0.8859 |
| H | 9.0581 | 5.6372 | -3.9003 | H | -1.5709 | 5.3376 | -2.427 |
| H | 8.5496 | 6.7725 | -5.1665 | H | -1.4015 | 3.7286 | -1.7174 |
| H | 4.2696 | 8.287 | -1.168 | H | -5.7092 | -3.7914 | -8.3246 |
| H | 2.5978 | 8.2527 | -0.5891 | H | -6.6528 | -3.6077 | -6.8384 |
| H | 3.6844 | 9.5669 | -0.0958 | H | -5.6821 | -2.2548 | -7.4363 |
| H | 2.0925 | 7.8823 | 1.9002 | H | -2.3052 | -3.8401 | -6.8003 |
| H | 3.4168 | 7.5603 | 3.0391 | H | -3.1176 | -2.4019 | -7.4541 |
| H | 3.2458 | 9.159 | 2.304 | H | -3.246 | -3.9527 | -8.2921 |
| H | 5.8451 | 7.4978 | 2.2018 | H | -3.6918 | -5.7681 | -5.8241 |
| H | 6.2157 | 7.8253 | 0.5033 | H | -4.541 | -5.8499 | -7.3807 |
| H | 5.5922 | 9.1228 | 1.5337 | H | -5.4598 | -5.7145 | -5.8745 |
| H | -5.8405 | -7.4756 | 2.2593 | H | -10.1081 | -7.3053 | -2.9689 |
| H | -6.2155 | -7.8136 | 0.5638 | H | -9.3336 | -5.9869 | -3.8547 |
| H | -5.5896 | -9.1047 | 1.6006 | H | -8.4151 | -6.9109 | -2.6474 |
| H | -2.0888 | -7.8625 | 1.9505 | H | -11.9652 | -5.8149 | -2.3057 |
| H | -3.4101 | -7.5335 | 3.0908 | H | -11.6847 | -4.318 | -1.4049 |
| H | -3.241 | -9.1367 | 2.365 | H | -11.2225 | -4.4175 | -3.1098 |
| H | -4.2737 | -8.286 | -1.1095 | H | -10.6619 | -7.1496 | -0.5695 |
| H | -2.6005 | -8.2484 | -0.5351 | H | -10.3463 | -5.6822 | 0.3685 |
| H | -3.686 | -9.5593 | -0.0309 | H | -8.9961 | -6.6993 | -0.1533 |
| H | -7.6584 | -6.6898 | -3.5895 | H | -0.3168 | 6.5868 | 3.3113 |
| H | -9.0691 | -5.6526 | -3.8451 | H | -1.9329 | 5.9027 | 3.4899 |
| H | -8.5646 | -6.7963 | -5.1054 | H | -1.3405 | 6.3709 | 1.8827 |
| H | -9.0233 | -3.8172 | -5.6414 | H | 0.552 | 4.5921 | 4.6024 |
| H | -7.5414 | -3.5255 | -6.5771 | H | 0.3487 | 2.9475 | 3.9727 |
| H | -8.4622 | -5.0157 | -6.8128 | H | -1.0446 | 3.815 | 4.6374 |
| H | -5.4458 | -4.9436 | -6.1415 | H | 1.5707 | 5.3371 | 2.4282 |
| H | -5.484 | -6.26 | -4.9604 | H | 1.4014 | 3.7281 | 1.7187 |
| H | -6.4372 | -6.3842 | -6.4467 | H | 0.7215 | 5.1284 | 0.8869 |
| H | -9.8251 | 3.7728 | -4.0109 | H | -8.6144 | 6.8852 | 3.9281 |
| H | -8.2656 | 4.2721 | -4.6994 | H | -8.9697 | 5.1613 | 3.7487 |
| H | -9.6684 | 4.042 | -5.7503 | H | -7.4178 | 5.6731 | 4.4279 |
| H | -9.892 | 0.3306 | -5.5797 | H | -5.706 | 6.912 | 2.968 |
| H | -10.7497 | 1.4263 | -4.4867 | H | -6.9781 | 8.0366 | 2.4774 |
| H | -10.6243 | 1.8108 | -6.2151 | H | -6.0392 | 7.1856 | 1.2451 |
| H | -6.9475 | 2.6555 | -6.2003 | H | -8.2384 | 6.3533 | 0.2128 |
| H | -7.6092 | 1.0647 | -6.6014 | H | -9.0977 | 7.2818 | 1.458 |
| H | -8.3941 | 2.5253 | -7.2208 | H | -9.4599 | 5.5653 | 1.2211 |
| H | -10.1385 | 4.6164 | 0.4649 | H | -13.4095 | 1.1542 | 2.9664 |
| H | -10.0559 | 4.251 | 2.1939 | H | -12.6961 | 1.0695 | 1.3432 |
| H | -10.4869 | 5.8847 | 1.6492 | H | -11.9463 | 2.0754 | 2.5911 |
| H | -8.2462 | 5.3239 | 3.665 | H | -12.7291 | -1.4865 | 1.5825 |
| H | -7.021 | 6.3845 | 2.937 | H | -11.9126 | -2.2625 | 2.9564 |
| H | -8.7074 | 6.9059 | 3.0258 | H | -13.374 | -1.3032 | 3.2174 |
| H | -7.0554 | 6.8428 | 0.4099 | H | -10.7191 | -0.8259 | 4.72 |
| H | -8.3419 | 6.1732 | -0.6036 | H | -12.246 | 0.0505 | 4.9472 |
| H | -8.7276 | 7.4052 | 0.6069 | H | -10.753 | 0.9395 | 4.6099 |
| H | 1.1569 | -3.2665 | 2.4244 | H | -3.1453 | 4.7488 | 2.1244 |
| H | -0.7972 | -4.6385 | 2.0438 | H | 0.0793 | 2.0024 | 1.7178 |
| H | 6.3958 | -4.1934 | 3.0168 | H | 3.1451 | 4.7492 | -2.1236 |
| H | 8.3646 | -3.8815 | -0.7513 | H | -0.0794 | 2.0026 | -1.7174 |
| H | 8.0773 | -3.3109 | -2.6568 | H | 7.5092 | -4.8815 | 3.1166 |
| H | 7.8457 | 0.3415 | -4.8509 | H | 10.0974 | -3.4452 | 0.0439 |
| H | 7.6622 | 2.2629 | -4.7205 | H | 10.5864 | -1.9997 | -1.1397 |
| H | 5.8057 | 5.6078 | -2.836 | H | 9.6876 | 1.831 | -2.7891 |
| H | 5.3746 | 6.3064 | -0.9994 | H | 8.8285 | 3.5578 | -2.1341 |
| H | 2.5289 | 5.6748 | 2.1234 | H | 4.9402 | 5.2482 | -1.6434 |
| H | -2.5247 | -5.6535 | 2.1616 | H | 2.6186 | -2.229 | 5.2556 |
| H | -5.3775 | -6.3042 | -0.9507 | H | 6.4125 | -4.0654 | 4.6119 |
| H | -5.8131 | -5.6174 | -2.7898 | H | -10.5869 | -1.9994 | 1.1386 |
| H | -7.6749 | -2.2842 | -4.6898 | H | -9.6881 | 1.8312 | 2.7882 |
| H | -7.8633 | -0.3587 | -4.8308 | H | -8.8287 | 3.5579 | 2.1335 |
| H | -8.0832 | 3.3047 | -2.6548 | H | -4.9403 | 5.248 | 1.6434 |
| H | -8.3607 | 3.8897 | -0.7609 | H | -7.5087 | -4.8821 | -3.1162 |
| H | -6.3845 | 4.2232 | 3.0012 | H | -10.0969 | -3.446 | -0.0435 |
| H | 1.4038 | -1.638 | 4.6277 | H | -2.6185 | -2.2288 | -5.2557 |
| H | 5.3187 | -3.3057 | 4.3487 | H | -6.4122 | -4.0657 | -4.6118 |
| H | 0.8017 | 4.6584 | 2.0173 | H | -0.9883 | -0.8 | 3.2849 |
| H | -1.1515 | 3.2885 | 2.4096 | H | 1.0377 | -1.5497 | 4.4193 |
| H | -5.3095 | 3.3398 | 4.3387 | H | -2.055 | -0.7487 | 1.1262 |
| H | -1.3948 | 1.6721 | 4.6217 | H | 2.0551 | -0.7485 | -1.1262 |

**Table S10.** Cartesian coordinates of optimized structures of **1** without *tert*-butyl groups (left) and transition state for (*P,P*)-(*M,M*) isomerization process of **1**.

| **1** no *t*Bu |  |  |  | TS **1** |  |  |  |
| --- | --- | --- | --- | --- | --- | --- | --- |
| **atom** | **x** | **y** | **z** | **atom** | **x** | **y** | **z** |
| C | 0.0313 | 2.8133 | -2.1206 | C | -1.1106 | -3.2088 | 2.1688 |
| C | -1.115 | 3.5131 | -1.9126 | C | -2.4079 | -3.6259 | 2.0788 |
| C | -2.326 | 2.8754 | -1.4986 | C | -3.3196 | -2.9866 | 1.1948 |
| C | -2.4197 | 1.4732 | -1.4512 | C | -2.8558 | -2.0301 | 0.2729 |
| C | 5.6159 | 2.8617 | -1.6179 | C | 2.9559 | 2.0782 | 3.1365 |
| C | 6.3617 | 4.0145 | -1.9108 | C | 2.5461 | 3.188 | 3.8912 |
| C | 7.2414 | 4.5507 | -0.9798 | C | 3.1483 | 4.4252 | 3.7036 |
| C | 7.39 | 3.9517 | 0.2657 | C | 4.1956 | 4.5606 | 2.7984 |
| C | 6.6713 | 2.7956 | 0.598 | C | 4.6332 | 3.474 | 2.0278 |
| C | 6.8665 | 2.1251 | 1.8812 | C | 5.8105 | 3.5614 | 1.1678 |
| C | 7.512 | 2.7396 | 2.9614 | C | 6.4931 | 4.7652 | 0.9467 |
| C | 7.7781 | 2.0271 | 4.1266 | C | 7.688 | 4.7882 | 0.236 |
| C | 7.457 | 0.6779 | 4.2114 | C | 8.2472 | 3.6043 | -0.2284 |
| C | 6.8085 | 0.0216 | 3.1533 | C | 7.5941 | 2.3771 | -0.0398 |
| C | 6.5568 | -1.4181 | 3.1592 | C | 8.1845 | 1.1147 | -0.475 |
| C | 7.0669 | -2.2545 | 4.1642 | C | 9.5334 | 1.0226 | -0.8477 |
| C | 6.8832 | -3.6306 | 4.1148 | C | 10.0742 | -0.1838 | -1.274 |
| C | 6.2083 | -4.2083 | 3.0454 | C | 9.2723 | -1.315 | -1.3684 |
| C | 5.6863 | -3.4143 | 2.0158 | C | 7.9178 | -1.2725 | -1.0085 |
| C | 4.9865 | -3.9921 | 0.8735 | C | 7.026 | -2.4144 | -1.198 |
| C | 5.0515 | -5.3601 | 0.5765 | C | 7.4729 | -3.6296 | -1.7348 |
| C | 4.3274 | -5.8992 | -0.4832 | C | 6.5825 | -4.6565 | -2.0366 |
| C | 3.4596 | -5.0955 | -1.2108 | C | 5.2187 | -4.4688 | -1.8633 |
| C | 3.3592 | -3.7229 | -0.9326 | C | 4.7338 | -3.2726 | -1.3108 |
| C | 5.8263 | -2.0025 | 2.0851 | C | 7.3745 | -0.055 | -0.5118 |
| C | 6.4363 | 0.7775 | 2.0074 | C | 6.3255 | 2.3613 | 0.604 |
| C | 5.7805 | 2.2315 | -0.3581 | C | 3.9605 | 2.2263 | 2.158 |
| C | 4.2047 | -3.1418 | 0.0419 | C | 5.6401 | -2.2676 | -0.8985 |
| C | 5.1114 | 0.9742 | -0.0923 | C | 4.3082 | 1.0888 | 1.3385 |
| C | 5.5858 | 0.1948 | 0.9981 | C | 5.5465 | 1.1454 | 0.6377 |
| C | 5.2331 | -1.165 | 1.0669 | C | 6.0185 | -0.0096 | -0.0163 |
| C | 4.3115 | -1.7042 | 0.1236 | C | 5.1412 | -1.1147 | -0.1907 |
| C | -3.3592 | 3.7229 | -0.9326 | C | -4.7338 | -3.2726 | 1.3108 |
| C | -3.4596 | 5.0955 | -1.2108 | C | -5.2187 | -4.4688 | 1.8633 |
| C | -4.3274 | 5.8992 | -0.4832 | C | -6.5825 | -4.6565 | 2.0367 |
| C | -5.0515 | 5.3601 | 0.5765 | C | -7.4729 | -3.6296 | 1.7349 |
| C | -4.9865 | 3.9921 | 0.8735 | C | -7.026 | -2.4144 | 1.1981 |
| C | -5.6863 | 3.4143 | 2.0158 | C | -7.9178 | -1.2725 | 1.0086 |
| C | -6.2083 | 4.2083 | 3.0454 | C | -9.2723 | -1.315 | 1.3684 |
| C | -6.8832 | 3.6307 | 4.1147 | C | -10.0741 | -0.1838 | 1.2741 |
| C | -7.0669 | 2.2546 | 4.1642 | C | -9.5334 | 1.0226 | 0.8477 |
| C | -6.5568 | 1.4181 | 3.1592 | C | -8.1845 | 1.1147 | 0.475 |
| C | -6.8086 | -0.0215 | 3.1533 | C | -7.5941 | 2.3771 | 0.0398 |
| C | -7.457 | -0.6778 | 4.2114 | C | -8.2472 | 3.6043 | 0.2284 |
| C | -7.7781 | -2.0271 | 4.1266 | C | -7.688 | 4.7882 | -0.236 |
| C | -7.512 | -2.7396 | 2.9614 | C | -6.4931 | 4.7652 | -0.9467 |
| C | -6.8665 | -2.1251 | 1.8812 | C | -5.8105 | 3.5614 | -1.1679 |
| C | -6.6713 | -2.7956 | 0.598 | C | -4.6332 | 3.474 | -2.0278 |
| C | -7.39 | -3.9517 | 0.2657 | C | -4.1956 | 4.5605 | -2.7984 |
| C | -7.2414 | -4.5507 | -0.9798 | C | -3.1483 | 4.4252 | -3.7036 |
| C | -6.3617 | -4.0145 | -1.9108 | C | -2.5461 | 3.188 | -3.8913 |
| C | -5.6159 | -2.8617 | -1.6179 | C | -2.9559 | 2.0782 | -3.1366 |
| C | -6.4363 | -0.7775 | 2.0074 | C | -6.3255 | 2.3613 | -0.604 |
| C | -5.8263 | 2.0025 | 2.0851 | C | -7.3745 | -0.055 | 0.5118 |
| C | -4.2047 | 3.1418 | 0.0419 | C | -5.6401 | -2.2676 | 0.8985 |
| C | -5.7805 | -2.2315 | -0.3581 | C | -3.9605 | 2.2263 | -2.158 |
| C | -4.3115 | 1.7042 | 0.1236 | C | -5.1412 | -1.1147 | 0.1907 |
| C | -5.2331 | 1.165 | 1.0669 | C | -6.0185 | -0.0096 | 0.0163 |
| C | -5.5858 | -0.1948 | 0.9981 | C | -5.5465 | 1.1454 | -0.6377 |
| C | -5.1114 | -0.9742 | -0.0923 | C | -4.3082 | 1.0888 | -1.3386 |
| C | 2.4345 | 1.275 | -2.7246 | C | 1.445 | -1.2834 | 2.3833 |
| C | 2.2718 | 1.8622 | -3.9829 | C | 0.9256 | -1.5583 | 3.6577 |
| C | 3.289 | 2.6226 | -4.5591 | C | 1.146 | -0.7424 | 4.7636 |
| C | 4.4108 | 2.9129 | -3.8042 | C | 1.7844 | 0.464 | 4.5762 |
| C | 4.5904 | 2.3542 | -2.5234 | C | 2.3658 | 0.7637 | 3.3316 |
| C | 3.6754 | 1.3681 | -2.0515 | C | 2.3877 | -0.1924 | 2.271 |
| C | 4.0595 | 0.5012 | -0.9282 | C | 3.447 | -0.0498 | 1.2553 |
| C | 3.5843 | -0.8533 | -0.7742 | C | 3.8008 | -1.0899 | 0.3208 |
| C | 1.2371 | 0.6464 | -2.1289 | C | 0.8042 | -1.8669 | 1.1836 |
| C | 0.0338 | 1.3901 | -2.0361 | C | -0.5687 | -2.3108 | 1.2086 |
| C | -1.2341 | 0.7267 | -1.8467 | C | -1.4063 | -1.9704 | 0.0865 |
| C | -1.2371 | -0.6464 | -2.1289 | C | -0.8042 | -1.8669 | -1.1836 |
| C | 1.115 | -3.5131 | -1.9126 | C | 2.4079 | -3.6259 | -2.0788 |
| C | 2.326 | -2.8754 | -1.4986 | C | 3.3196 | -2.9866 | -1.1948 |
| C | 2.4197 | -1.4732 | -1.4512 | C | 2.8559 | -2.0301 | -0.2729 |
| C | 1.2341 | -0.7267 | -1.8467 | C | 1.4063 | -1.9704 | -0.0865 |
| C | -0.0338 | -1.3901 | -2.0361 | C | 0.5687 | -2.3108 | -1.2086 |
| C | -0.0313 | -2.8133 | -2.1205 | C | 1.1106 | -3.2088 | -2.1688 |
| C | -3.5843 | 0.8533 | -0.7742 | C | -3.8008 | -1.0899 | -0.3208 |
| C | -4.0595 | -0.5013 | -0.9282 | C | -3.447 | -0.0498 | -1.2553 |
| C | -3.6754 | -1.3681 | -2.0515 | C | -2.3877 | -0.1925 | -2.271 |
| C | -4.5904 | -2.3542 | -2.5234 | C | -2.3659 | 0.7637 | -3.3316 |
| C | -4.4108 | -2.9129 | -3.8042 | C | -1.7844 | 0.464 | -4.5763 |
| C | -3.289 | -2.6226 | -4.5591 | C | -1.1461 | -0.7425 | -4.7636 |
| C | -2.2718 | -1.8622 | -3.9829 | C | -0.9256 | -1.5583 | -3.6577 |
| C | -2.4345 | -1.275 | -2.7246 | C | -1.445 | -1.2834 | -2.3833 |
| H | 0.9618 | 3.3399 | -2.2931 | H | -0.4598 | -3.7009 | 2.8786 |
| H | -1.0708 | 4.5954 | -1.9163 | H | -2.7691 | -4.3837 | 2.7638 |
| H | 6.2361 | 4.5215 | -2.8599 | H | 1.7299 | 3.0912 | 4.5985 |
| H | 7.8119 | 5.4425 | -1.2244 | H | 2.8133 | 5.2854 | 4.2765 |
| H | 8.1007 | 4.3685 | 0.9704 | H | 4.6884 | 5.5221 | 2.7134 |
| H | 7.7919 | 3.7858 | 2.9051 | H | 6.0921 | 5.6984 | 1.3251 |
| H | 8.2574 | 2.5224 | 4.9665 | H | 8.1936 | 5.7331 | 0.0572 |
| H | 7.7062 | 0.1388 | 5.1182 | H | 9.1861 | 3.6428 | -0.7691 |
| H | 7.6316 | -1.8345 | 4.9884 | H | 10.1772 | 1.8919 | -0.778 |
| H | 7.2775 | -4.2579 | 4.9095 | H | 11.1248 | -0.2412 | -1.5447 |
| H | 6.0674 | -5.2832 | 3.0278 | H | 9.7126 | -2.2343 | -1.7361 |
| H | 5.6995 | -6.0084 | 1.1558 | H | 8.5272 | -3.7844 | -1.9319 |
| H | 4.424 | -6.954 | -0.7245 | H | 6.9565 | -5.5962 | -2.4335 |
| H | 2.8727 | -5.5321 | -2.0115 | H | 4.5302 | -5.2646 | -2.1264 |
| H | -2.8726 | 5.5321 | -2.0115 | H | -4.5302 | -5.2646 | 2.1265 |
| H | -4.424 | 6.954 | -0.7246 | H | -6.9565 | -5.5962 | 2.4335 |
| H | -5.6995 | 6.0084 | 1.1558 | H | -8.5272 | -3.7844 | 1.9319 |
| H | -6.0674 | 5.2832 | 3.0278 | H | -9.7126 | -2.2343 | 1.7362 |
| H | -7.2775 | 4.2579 | 4.9094 | H | -11.1248 | -0.2412 | 1.5448 |
| H | -7.6316 | 1.8346 | 4.9884 | H | -10.1772 | 1.892 | 0.7781 |
| H | -7.7062 | -0.1388 | 5.1182 | H | -9.186 | 3.6428 | 0.7691 |
| H | -8.2575 | -2.5224 | 4.9665 | H | -8.1936 | 5.7331 | -0.0572 |
| H | -7.792 | -3.7858 | 2.9051 | H | -6.0921 | 5.6984 | -1.3252 |
| H | -8.1007 | -4.3685 | 0.9704 | H | -4.6884 | 5.5221 | -2.7135 |
| H | -7.8119 | -5.4425 | -1.2244 | H | -2.8134 | 5.2854 | -4.2766 |
| H | -6.2361 | -4.5215 | -2.8599 | H | -1.7299 | 3.0911 | -4.5986 |
| H | 1.3259 | 1.7346 | -4.4997 | H | 0.2756 | -2.4012 | 3.8031 |
| H | 3.1711 | 3.036 | -5.5567 | H | 0.7487 | -1.0231 | 5.7347 |
| H | 5.1589 | 3.5854 | -4.2072 | H | 1.8858 | 1.1663 | 5.3956 |
| H | 1.0708 | -4.5954 | -1.9163 | H | 2.7691 | -4.3837 | -2.7638 |
| H | -0.9618 | -3.3399 | -2.293 | H | 0.4598 | -3.7009 | -2.8786 |
| H | -5.159 | -3.5854 | -4.2072 | H | -1.8858 | 1.1663 | -5.3956 |
| H | -3.1711 | -3.036 | -5.5567 | H | -0.7488 | -1.0232 | -5.7348 |
| H | -1.3259 | -1.7346 | -4.4997 | H | -0.2756 | -2.4012 | -3.8031 |

**3.4 Mechanistic calculations**

The Mechanistic calculations were performed by density functional theory (DFT) calculations using the ωB97X-D functional. Frequency calculations and single-point energy calculations were also performed on the optimized geometries at the def2-SVP and def2-TZVP levels, respectively.

During the formation of compound **2** from a 1,5-functionalized anthracene precursor, the initial step is proposed to proceed via a plausible intramolecular cyclization, leading to the formation of seven-membered rings firstly, followed by a subsequent Scholl-type oxidative cyclization to afford NG **2**. As the initial intramolecular cyclization is considered to be the key step governing the observed diastereoselectivity, our mechanistic studies were therefore focused on this step.

Accordingly, the mechanism of the intramolecular cyclization reaction toward the diastereoselective formation of compound **K** (conceptual precursor to observed compound **2** with *syn*-positioned H-atoms) over **J** (conceptual precursor with *anti*-positioned H-atoms) is presented below in Figure S17. The calculation results show that the transition states **F** and **G** display a Gibbs free-energy difference ΔΔG^‡^ of approximately 40 kJ mol^-1^. The lower energy barrier associated with transition state **G** indicates that this pathway is kinetically favored, predominantly leading to **K** (*syn*-), in which the two hydrogens are located on the same side of the former anthracene moiety. In addition, according to the equation $\frac{K(Syn)}{J (Anti)}=e^{G\ddagger/RT}$ (derived from the Eyring equation), the calculated Gibbs free-energy difference of 40 kJ mol⁻¹ corresponds to a predicted *syn*/*anti* ratio of approximately 1 × 10^9^:1, indicating essentially exclusive *syn* pathway under kinetic control.^[24–27]^

These calculations support the experimentally observed diastereoselective formation of compound **2** under the reaction conditions.


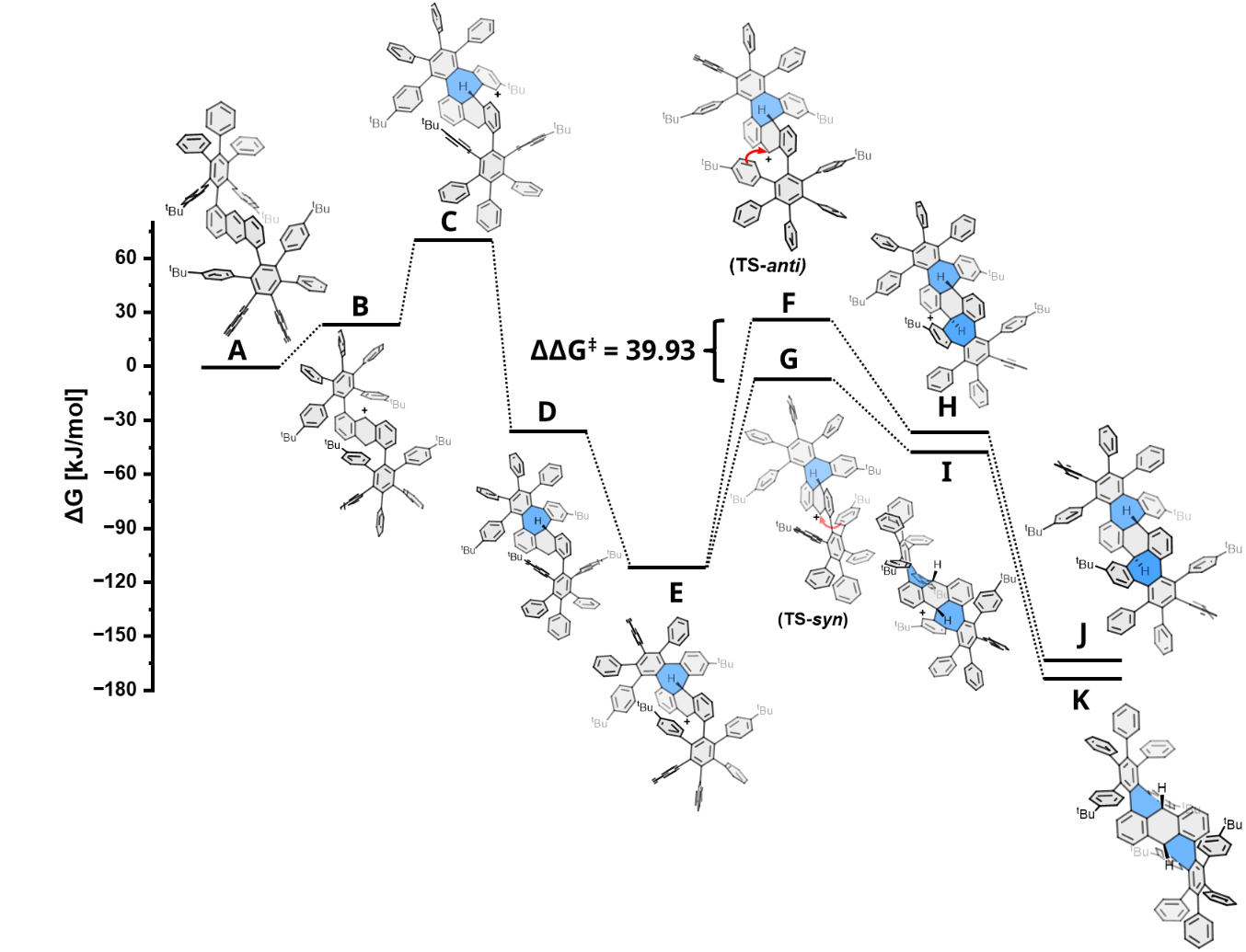


**Figure S17.** Energy diagram of the suggested mechanism for the selective formation of **K** (*syn*-product) over **J** (*anti*-product) *via* transition states **F** and **G**.

Further calculations regarding the oxidation of compound **2** to compound **1** were conducted. The proposed mechanism is depicted in Figure S18. To reduce computational cost, the *tert*-butyl groups on the outward facing benzene rings were substituted by H-atoms. As shown in Figure S18, educts **A** (simplified compound **2**) and **B** (DDQ) react *via* transition state **C**. This transition state is characterized by a hydride transfer from the *sp*^3^-defect carbon center to DDQ. The resulting charge-separated intermediate **D** subsequently undergoes elimination of a proton (transfer of H^+^ from **2**^+^ onto DDQ-H^-^) to afford DDQ-H_2_ (**F**) and simplified chiral saddle **1** (**E**). The high activation barrier (ΔG^‡^ ≈ 147 kJ mol⁻¹) is consistent with the experimentally observed slow reaction rate (4 days at 100 °C for full conversion).


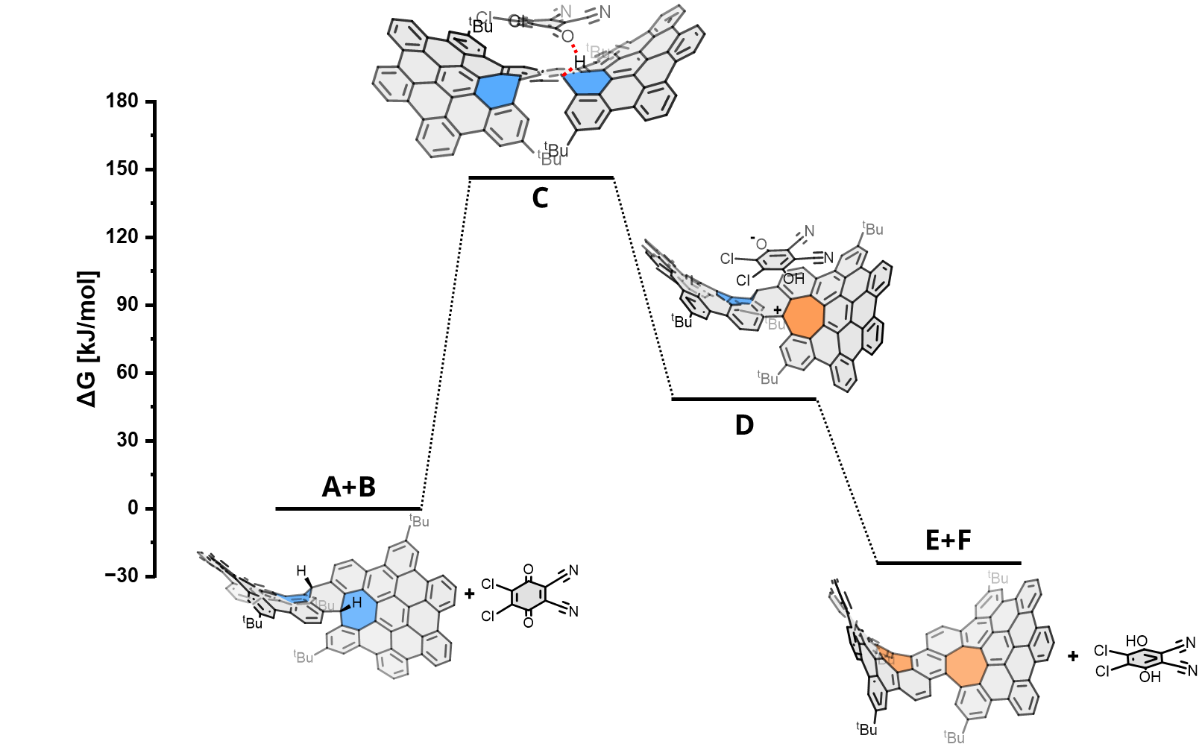


**Figure S18.** Proposed reaction mechanism for the oxidation of simplified compound **2** (**A**) to simplified compound **1** (**E**); red dotted lines in **C** indicate transition state interaction between O atom of DDQ, and H atom and C atom of simplified compound **2**.

# **4. Cyclic voltammetry (CV) and square wave voltammetry (SWV)**

The cyclic voltammetry (CV) experiments were performed in a solution with concentration *c* ~ 10^-3^ M in degassed dry CH_2_Cl_2_ with 0.1 M Bu_4_NPF_6_ as the supporting electrolyte. Silver/silver chloride (Ag/AgCl) was used as a reference electrode. The spectra were recorded with a scan rate of 50 mV s^-1^. Potentials were referenced to ferrocenium/ferrocene (Fc^+^/Fc^0^). The square wave voltammetry (SWV) experiments were obtained by using the same solutions. The HOMO and LUMO energies are calculated with the following formulas:

$$E_{HOMO}\left( eV \right)\text{=-}e\left[ E_{ox}\text{+}4.8 \right],\text{ }Equation\text{ }1$$

$$E_{LUMO}\left( eV \right)\text{=-}e\left[ E_{red}\text{+}4.8 \right],\text{ }Equation\text{ }2$$

where *E*_ox_, *E*_red_ are the corresponding oxidation and reduction offsets of the compound, referenced to Fc^+^/Fc^0^ in the corresponding solvent.

# **5. CPL experiments**

*Procedure*: A CH_2_Cl_2_ solution containing the respective enantiomers was scanned 20 times (wavelength region of 425–625 nm for **2** and 500–700 nm for **1**) at 20 °C. Due to the weak luminescence of compound **2** and the detection limitations of the CPL instrument, the CPL spectra of NG **2** exhibit a poor signal-to-noise ratio.

The CPL spectra of the enantiomers of **1** were recorded as shown in Figure S19. The dissymmetry factors of approximately *g*_lum_ = 0.003 for (*M*,*M*)-**1** and *g*_lum_ = -0.003 for (*P*,*P*)-**1** can be observed at 550 nm.


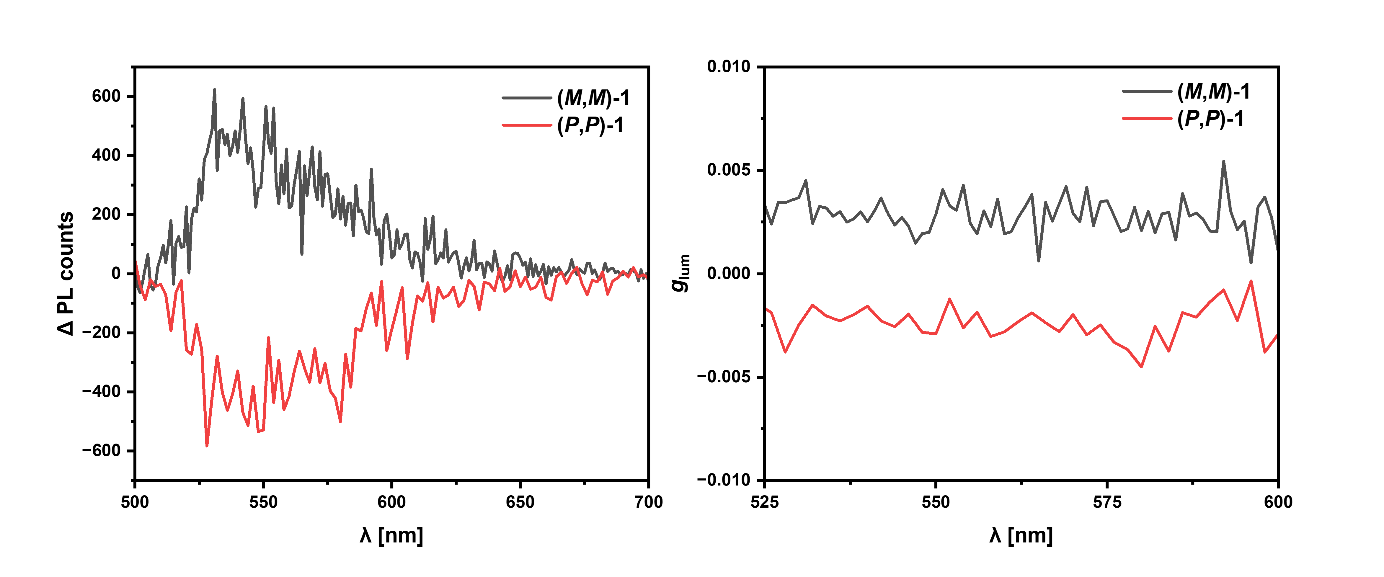


**Figure S19.** CPL spectra (left) for enantiomers of NGs **1** and their *g*_lum_ (right).

Below, the *g*_abs_ and *g*_lum_ values of chiral saddle **1** and related chiral nanographenes are summarized.

**Table S11.** Absorption and luminescence dissymmetry factors of selected nanographenes previously reported.

| **Structure** | ***g*_abs_** | ***g*_lum_** | **DOI** |
| --- | --- | --- | --- |
|  | 1.1 × 10^-3^ | 2.5 × 10^-3^ | 10.1002/chir.23535 |
|  | 4.2 × 10^-2^ | Not reported | 10.1021/jacs.5c22771 |
|  | 2.0 × 10^-3^ | 2.0 × 10^-3^ | 10.1039/D1CC02293H |
|  | 3.0 × 10^-3^ | 3.0 × 10^-3^ | 10.1039/D2SC03452B |
|  | 3.9 × 10^-3^ | Not reported | 10.1002/anie.201912213 |
|  | 8.2 × 10^-3^ | 0.3 × 10^-3^ | 10.1002/anie.201902529 |
|  | 4.5 × 10^-3^ | 2.4 × 10^-3^ | 10.1002/anie.202400172 |
|  | 4.6 × 10^-3^ | 6.1 × 10^-4^ | 10.1021/acs.orglett.6c00388 |
|  | Not reported | Not reported | 10.1021/jacs.0c05504 |
|  | Not reported | Not reported | 10.1021/jacs.0c06156 |
|  | 6.6 × 10^-3^ | Not reported | 10.1002/anie.8159257 |
|  | 6.2 × 10^-4^ | Not reported |  |
|  | 4.9 × 10^-3^ | Not reported |  |
|  | 6.5 × 10^-4^ | Not determined | This work |
|  | 4.2 × 10^-3^ | 3.0 × 10^-3^ | This work |

# **6. UV-Vis titration experiments**

*Procedure*: A toluene solution containing **1** was mixed with separate solutions of C_60_ or C_70_ (also in toluene) in different ratios at 20 °C. The corresponding concentrations of the solutions for the measurements are:

- For the experiment with C_60_: **1** (*c* = 10.94 × 10^-6^ M), C_60_ (*c* = 10.94 × 10^-6^ M)
- For the experiment with C_70_: **1** (*c* = 10.94 × 10^-6^ M), C_70_ (*c* = 10.94 × 10^-6^ M)

The UV-Vis absorption spectra were recorded and the absorbance was plotted. The corresponding Job’s plots were constructed by tracing the absorbance decrease of **1** at 408 nm.

**Table S12.** Absorbance values of **1** at 408 nm, respective molar fractions of fullerene, and its product used in the determination of binding modes *via* the Job’s plot analysis.

| **A_408_ [a.u.]** | **χ(C_60_)** | **χ(C_60_) × A_408_** |  | **A_408_ [a.u.]** | **χ(C_70_)** | **χ(C_70_) × A_408_** |
| --- | --- | --- | --- | --- | --- | --- |
| 0.44313 | 0 | 0 |  | 0.44313 | 0 | 0 |
| 0.40425 | 0.09091 | 0.03675 |  | 0.40355 | 0.09091 | 0.03669 |
| 0.36002 | 0.18182 | 0.06546 |  | 0.359 | 0.18182 | 0.06527 |
| 0.31984 | 0.27273 | 0.08723 |  | 0.31388 | 0.27273 | 0.0856 |
| 0.28253 | 0.36364 | 0.10274 |  | 0.27728 | 0.36364 | 0.10083 |
| 0.24084 | 0.45455 | 0.10947 |  | 0.23497 | 0.45455 | 0.1068 |
| 0.20316 | 0.54545 | 0.11082 |  | 0.19518 | 0.54545 | 0.10646 |
| 0.15647 | 0.63636 | 0.09957 |  | 0.1553 | 0.63636 | 0.09883 |
| 0.12075 | 0.72727 | 0.08782 |  | 0.11609 | 0.72727 | 0.08443 |
| 0.0839 | 0.81818 | 0.06864 |  | 0.07737 | 0.81818 | 0.0633 |
| 0.04244 | 0.90909 | 0.03858 |  | 0.0392 | 0.90909 | 0.03564 |
| 0 | 1 | 0 |  | 0 | 1 | 0 |

# **7. Stern-Volmer titration experiments**

*Procedure*: To a solution of **1** in toluene different amounts of C_60_ or C_70_ (also toluene solutions) were added at 20 °C. The corresponding concentrations of the solutions for the measurements are:

- For the experiment with C_60_: **1** (*c* = 1.54 × 10^-6^ M), C_60_ (*c* = 4.63 × 10^-4^ M)
- For the experiment with C_70_: **1** (*c* = 1.54 × 10^-6^ M), C_70_ (*c* = 4.63 × 10^-4^ M)

The photoluminescence (PL) spectra of **1** at different host-guest ratios was recorded and plotted. The binding constant (*K*_a_) was determined using the fluorescence intensity at 535 nm by **Equation 3**:

$$\frac{F}{F_{0}}\text{=}\frac{1\text{+}AK_{a}\left[ G \right]}{1\text{+}K_{a}\left[ G \right]},\text{ }Equation\text{ }3$$

Here, *F* is the PL intensity at each concentration of guest solution, *F*_0_ is the fluorescence of the host without any guest addition, [G] is the concentration of the guest and *A* is a ratio of a proportionality constants.

**Table S13.** Obtained values from titration of **1** with C_60_ and C_70_ used in the determination of binding constants *via* **Equation 3**.

| **c(C_60_) [µM]** | **F/F_0_** | **equiv C_60_** |  | **c(C_70_)[µM]** | **F/F_0_** | **equiv C_70_** |
| --- | --- | --- | --- | --- | --- | --- |
| 0.00 | 1.00 | 0.00 |  | 0.00 | 1.00 | 0.00 |
| 1.54 | 0.95 | 1.00 |  | 0.77 | 0.94 | 0.50 |
| 2.30 | 0.93 | 1.50 |  | 1.54 | 0.89 | 1.00 |
| 3.07 | 0.91 | 2.00 |  | 2.30 | 0.83 | 1.50 |
| 3.83 | 0.90 | 2.50 |  | 3.07 | 0.79 | 2.00 |
| 5.34 | 0.87 | 3.50 |  | 3.83 | 0.74 | 2.50 |
| 6.84 | 0.85 | 4.50 |  | 4.58 | 0.70 | 3.00 |
| 8.33 | 0.82 | 5.50 |  | 5.34 | 0.67 | 3.50 |
| 9.82 | 0.80 | 6.50 |  | 6.09 | 0.63 | 4.00 |
| 11.29 | 0.78 | 7.50 |  | 7.59 | 0.57 | 5.00 |
| 12.88 | 0.76 | 8.50 |  | 9.08 | 0.52 | 6.00 |
| 14.21 | 0.74 | 9.50 |  | 12.02 | 0.43 | 8.00 |
| 15.65 | 0.72 | 10.50 |  | 14.93 | 0.36 | 10.00 |
| 22.74 | 0.65 | 15.50 |  | 22.04 | 0.24 | 15.00 |
| 29.61 | 0.59 | 20.50 |  | 28.93 | 0.17 | 20.00 |
| 42.72 | 0.49 | 30.50 |  | 42.08 | 0.09 | 30.00 |
| 55.06 | 0.42 | 40.50 |  | 66.13 | 0.04 | 50.00 |
| 66.69 | 0.37 | 50.50 |  |  |  |  |
| 77.69 | 0.33 | 60.50 |  |  |  |  |
| 88.08 | 0.30 | 70.50 |  |  |  |  |
| 97.93 | 0.27 | 80.50 |  |  |  |  |
| 107.28 | 0.25 | 90.50 |  |  |  |  |

# **8. NMR titration experiments**

*Procedure for C_60_ titration*: 4.07 mg of **1** were dissolved in 2.50 mL toluene-d_8_ and the resulting stock solution was used for the preparation of host and guest solutions, as follows:

- Host solution: 0.5 mL of the stock solution was filled into an NMR tube
  (*c*_Host_ = 1.00 × 10^-3^ M).
- Guest solution: 2.00 mL of the stock solution was filled into a 3 mL GC-vial containing 5.77 mg of C_60_. The solution was sonicated for 10 min
  [(*c*_Host_ = 1.00 × 10^-3^ M), *c*_Guest_ = 4.00 × 10^-3^ M, *c*_Host_/*c*_Guest_ = 1/4)].

Distinct aliquots of the guest solution were added to the host solution. Subsequently, the ^1^H NMR spectrum of the mixture was acquired at a temperature of 23 °C.

**Table S14.** ^1^H chemical shifts of the detected signals at different c(**1**) to c(C_60_) ratios and total shifts Δδ after addition of 4 aliquotes of C_60_ (1:C_60_ = 1:4).

| **c(1) [M]** | **c(C_60_) [M]** | **Proton 17** | **Proton 13** | **Proton 14** | **Proton 15** | **Proton 16** | **Proton 1** |
| --- | --- | --- | --- | --- | --- | --- | --- |
| 0.0010 | 0.0000 | 1.5212 | 1.5433 | 1.5610 | 1.5853 | 1.6829 | 7.6340 |
| 0.0010 | 0.0003 | 1.5214 | 1.5415 | 1.5812 | 1.6022 | 1.6810 | 7.6563 |
| 0.0010 | 0.0006 | 1.5221 | 1.5393 | 1.5964 | 1.6167 | 1.6784 | 7.6803 |
| 0.0010 | 0.0010 | 1.5244 | 1.5380 | 1.6127 | 1.6329 | 1.6759 | 7.7128 |
| 0.0010 | 0.0013 | 1.5251 | 1.5396 | 1.6196 | 1.6387 | 1.6762 | 7.7278 |
| 0.0010 | 0.0018 | 1.5251 | 1.5439 | 1.6227 | 1.6422 | 1.6788 | 7.7406 |
| 0.0010 | 0.0021 | 1.5249 | 1.5467 | 1.6238 | 1.6429 | 1.6807 | 7.7453 |
| 0.0010 | 0.0025 | 1.5246 | 1.5473 | 1.6241 | 1.6430 | 1.6811 | 7.7464 |
| 0.0010 | 0.0027 | 1.5240 | 1.5545 | 1.6260 | 1.6436 | 1.6860 | 7.7556 |
| 0.0010 | 0.0031 | 1.5237 | 1.5584 | 1.6267 | 1.6438 | 1.6886 | 7.7593 |
| 0.0010 | 0.0033 | 1.5235 | 1.5604 | 1.6273 | 1.6438 | 1.6900 | 7.7613 |
| 0.0010 | 0.0034 | 1.5236 | 1.5607 | 1.6271 | 1.6438 | 1.6902 | 7.7614 |
| 0.0010 | 0.0040 | 1.5231 | 1.5662 | 1.6280 | 1.6438 | 1.6940 | 7.7666 |
|  |  |  |  |  |  |  |  |
|  | **Δδ [ppm]** | 0.0019 | 0.0229 | 0.0670 | 0.0585 | 0.0111 | 0.1326 |

| **c(1) [M]** | **c(C_60_) [M]** | **Proton 12** | **Proton 2** | **Proton 3** | **Proton 4** | **Proton 5** |
| --- | --- | --- | --- | --- | --- | --- |
| 0.0010 | 0.0000 | 7.8938 | 8.3623 | 8.7808 | 9.0037 | 9.0183 |
| 0.0010 | 0.0003 | 7.9250 | 8.3860 | 8.7873 | 9.0022 | 9.0172 |
| 0.0010 | 0.0006 | 7.9085 | 8.4101 | 8.7944 | 9.0027 | 9.0188 |
| 0.0010 | 0.0010 | 7.9168 | 8.4421 | 8.8052 | 9.0048 | 9.0221 |
| 0.0010 | 0.0013 | 7.9211 | 8.4572 | 8.8114 | 9.0068 | 9.0240 |
| 0.0010 | 0.0018 | 7.9252 | 8.4710 | 8.8186 | 9.0097 | 9.0258 |
| 0.0010 | 0.0021 | 7.9267 | 8.4765 | 8.8218 | 9.0110 | 9.0264 |
| 0.0010 | 0.0025 | 7.9269 | 8.4777 | 8.8224 | 9.0111 | 9.0265 |
| 0.0010 | 0.0027 | 7.9310 | 8.4887 | 8.8300 | 9.0148 | 9.0283 |
| 0.0010 | 0.0031 | 7.9322 | 8.4935 | 8.8335 | 9.0162 | 9.0285 |
| 0.0010 | 0.0033 | 7.9326 | 8.4960 | 8.8353 | 9.0168 | 9.0285 |
| 0.0010 | 0.0034 | 7.9326 | 8.4962 | 8.8354 | 9.0168 | 9.0285 |
| 0.0010 | 0.0040 | 7.9346 | 8.5027 | 8.8403 | 9.0189 | 9.0290 |
|  |  |  |  |  |  |  |
|  | **Δδ [ppm]** | **0.0408** | **0.1404** | **0.0595** | **0.0152** | **0.0107** |

| **c(1) [M]** | **c(C_60_) [M]** | **Proton 9** | **Proton 11** | **Proton 8** | **Proton 10** | **Proton 6/7** |
| --- | --- | --- | --- | --- | --- | --- |
| 0.0010 | 0.0000 | 9.1042 | 9.1188 | 9.1300 | 9.1740 | 9.1941 |
| 0.0010 | 0.0003 | 9.0992 | 9.1200 | 9.1344 | 9.1730 | 9.2084 |
| 0.0010 | 0.0006 | 9.0964 | 9.1220 | 9.1397 | 9.1695 | 9.2294 |
| 0.0010 | 0.0010 | 9.0942 | 9.1245 | 9.1464 | 9.1679 | 9.2515 |
| 0.0010 | 0.0013 | 9.0941 | 9.1256 | 9.1493 | 9.1681 | 9.2609 |
| 0.0010 | 0.0018 | 9.0948 | 9.1265 | 9.1512 | 9.1697 | 9.2675 |
| 0.0010 | 0.0021 | 9.0953 | 9.1267 | 9.1517 | 9.1705 | 9.2692 |
| 0.0010 | 0.0025 | 9.0955 | 9.1267 | 9.1517 | 9.1707 | 9.2697 |
| 0.0010 | 0.0027 | 9.0974 | 9.1277 | 9.1531 | 9.1739 | 9.2699 |
| 0.0010 | 0.0031 | 9.0982 | 9.1275 | 9.1531 | 9.1750 | 9.2714 |
| 0.0010 | 0.0033 | 9.0984 | 9.1275 | 9.1530 | 9.1756 | 9.2705 |
| 0.0010 | 0.0034 | 9.0984 | 9.1274 | 9.1529 | 9.1757 | 9.2706 |
| 0.0010 | 0.0040 | 9.0997 | 9.1275 | 9.1533 | 9.1776 | 9.2714 |
|  |  |  |  |  |  |  |
|  | **Δδ [ppm]** | **-0.0045** | **0.0087** | **0.0233** | **0.0036** | **0.0773** |

The ^1^H chemical shifts of all protons in the obtained spectra were fitted using the Bindfitv0.5 software ([http://supramolecular.org](http://supramolecular.org/)) with the theoretical model H:G = 1:2. For assignment of protons see the synthetic section for compound **1**.


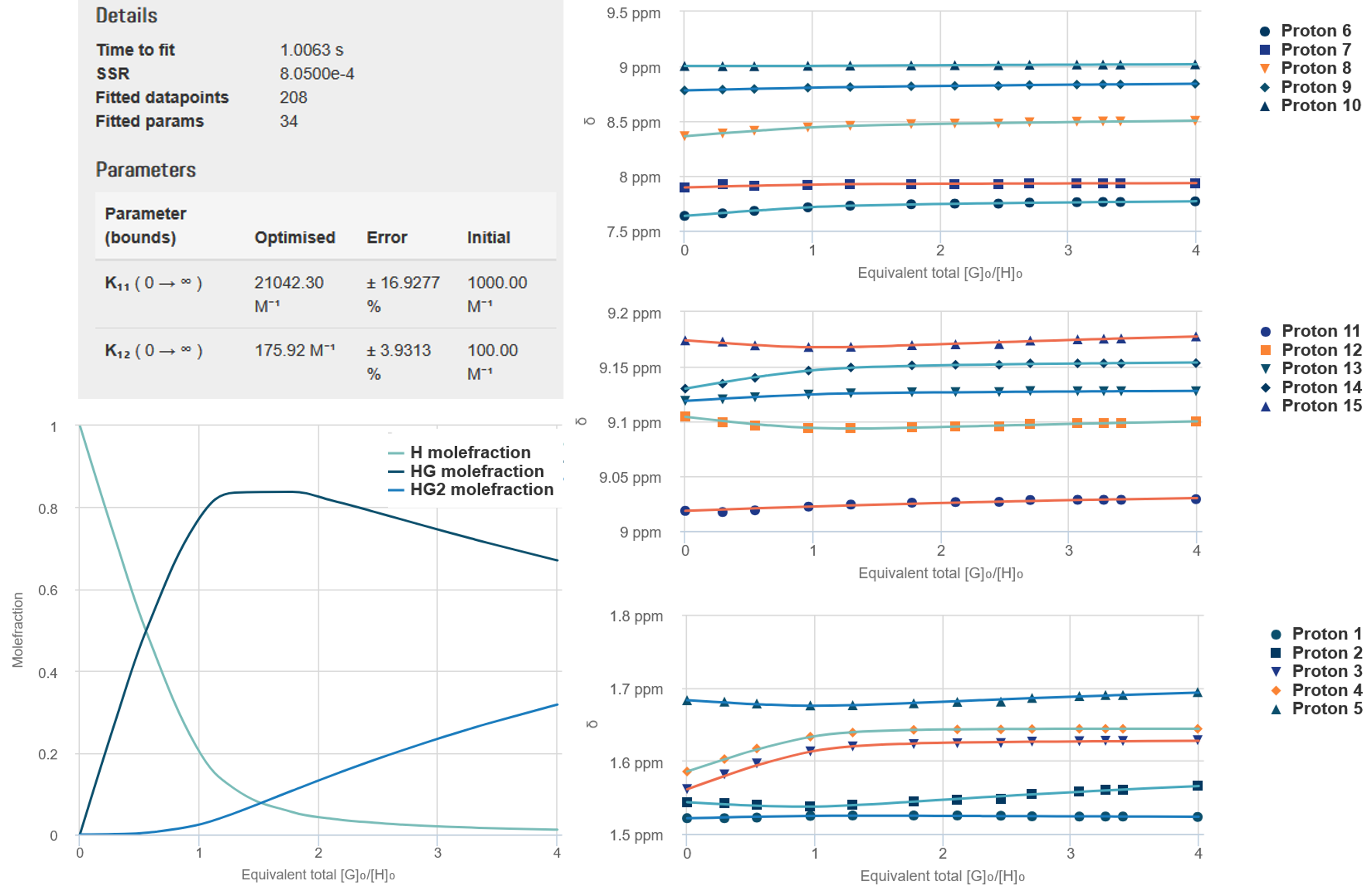


**Figure S20.** Fitting results (top left), molar fractions plot (bottom left), and ^1^H chemical shifts with fits (right) obtained from BindFit v.05 for the titration of **1** with C_60_.


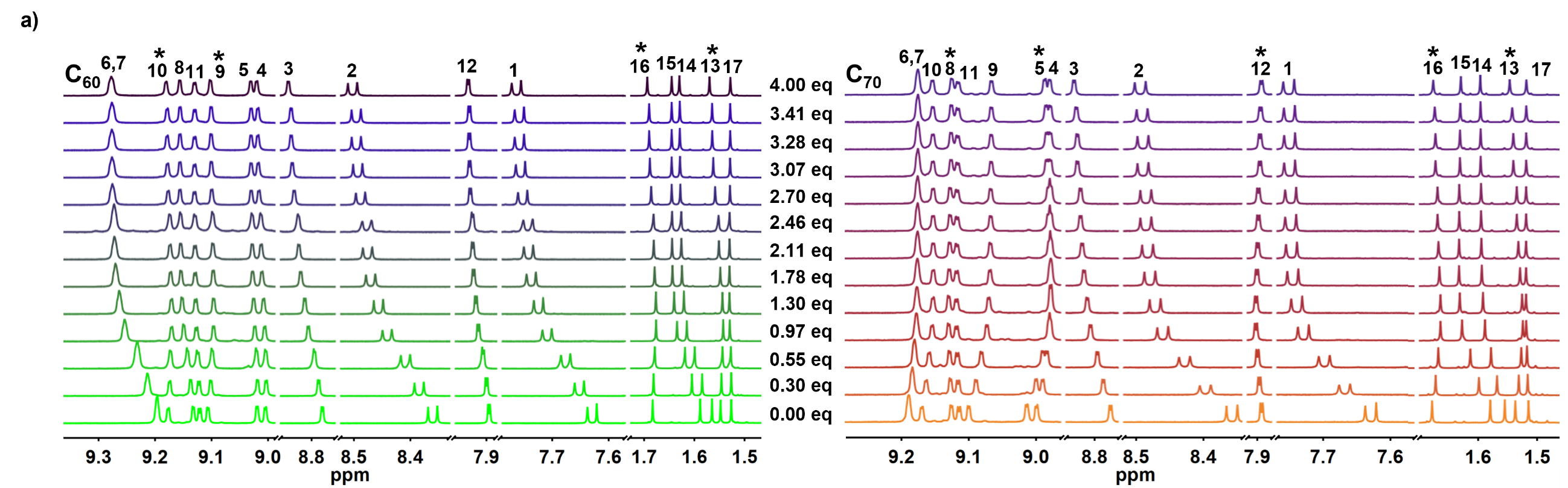

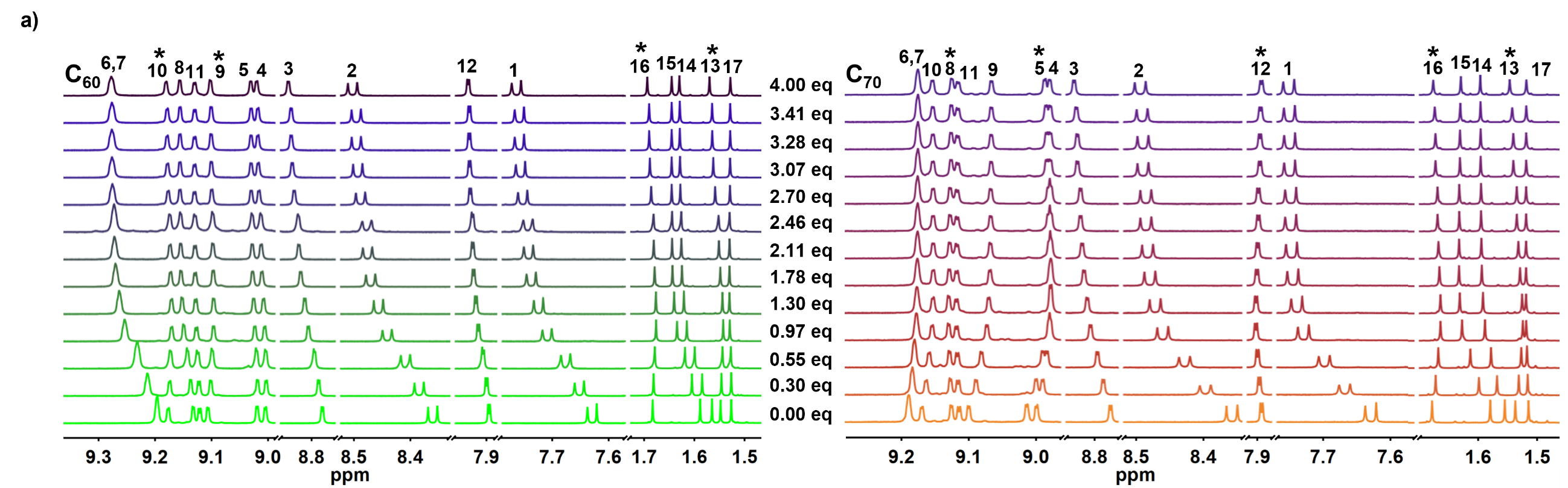


**Figure S21.** Stacked ^1^H NMR spectra of different ratios of **1** and C_60_ at constant concentration of **1** (toluene-d_8_, 295 K); protons labelled with numbers, signals displaying a reversal in chemical shift displacement marked with *****.

*Procedure for C_70_ titration*: 1.36 mg of **1** were dissolved 2.50 mL toluene-d_8_ and the resulting stock solution was used for the preparation of host and guest solutions, as follows:

- Host solution: 0.5 mL of the stock solution was filled into an NMR tube
  (*c*_Host_ = 3.33 × 10^-4^ M).
- Guest solution: 2.00 mL of the stock solution was filled into a 3 mL GC-vial containing 2.24 mg of C_70_. The solution was sonicated for 10 min
  [(*c*_Host_ = 3.33 × 10^-4^ M), *c*_Guest_ = 1.33 × 10^-3^ M, *c*_Host_/*c*_Guest_ = 1/4)].

Distinct aliquots of the guest solution were added to the host solution. Subsequently, the ^1^H NMR spectrum of the mixture was acquired at a temperature of 23 °C.

**Table S15.** ^1^H chemical shifts of the detected signals at different c(**1**) to c(C_70_) ratios and total shifts Δδ after addition of 4 aliquotes of C_60_ (1:C_70_ = 1:4).

| **c(1) [M]** | **c(C_70_) [M]** | **Proton 17** | **Proton 13** | **Proton 14** | **Proton 15** | **Proton 16** | **Proton 1** |
| --- | --- | --- | --- | --- | --- | --- | --- |
| 0.00033 | 0.00000 | 1.51560 | 1.53780 | 1.55540 | 1.57980 | 1.67730 | 7.62830 |
| 0.00033 | 0.00010 | 1.51710 | 1.53200 | 1.56840 | 1.59890 | 1.67140 | 7.66700 |
| 0.00033 | 0.00018 | 1.51820 | 1.52780 | 1.57850 | 1.61310 | 1.66690 | 7.69720 |
| 0.00033 | 0.00032 | 1.51960 | 1.52510 | 1.58870 | 1.62720 | 1.66330 | 7.72820 |
| 0.00033 | 0.00043 | 1.51980 | 1.52640 | 1.59200 | 1.63090 | 1.66330 | 7.73810 |
| 0.00033 | 0.00059 | 1.51980 | 1.53000 | 1.59390 | 1.63220 | 1.66490 | 7.74390 |
| 0.00033 | 0.00070 | 1.51980 | 1.53270 | 1.59470 | 1.63200 | 1.66660 | 7.74580 |
| 0.00033 | 0.00082 | 1.51980 | 1.53540 | 1.59510 | 1.63170 | 1.66820 | 7.74700 |
| 0.00033 | 0.00090 | 1.51970 | 1.53540 | 1.59520 | 1.63170 | 1.66820 | 7.74700 |
| 0.00033 | 0.00102 | 1.51980 | 1.54100 | 1.59590 | 1.63050 | 1.67160 | 7.74860 |
| 0.00033 | 0.00109 | 1.51950 | 1.54110 | 1.59590 | 1.63050 | 1.67160 | 7.74860 |
| 0.00033 | 0.00114 | 1.51930 | 1.54290 | 1.59610 | 1.63000 | 1.67280 | 7.74900 |
| 0.00033 | 0.00133 | 1.51930 | 1.54720 | 1.59650 | 1.62910 | 1.67540 | 7.74990 |
|  |  |  |  |  |  |  |  |
|  | **Δδ [ppm]** | **0.00370** | **0.00940** | **0.04110** | **0.04930** | **-0.00190** | **0.12160** |

| **c(1) [M]** | **c(C_70_) [M]** | **Proton 12** | **Proton 2** | **Proton 3** | **Proton 4** | **Proton 5** |
| --- | --- | --- | --- | --- | --- | --- |
| 0.00033 | 0.00000 | 7.89250 | 8.35650 | 8.77510 | 8.99810 | 9.01260 |
| 0.00033 | 0.00010 | 7.89570 | 8.39610 | 8.78580 | 8.98900 | 8.99870 |
| 0.00033 | 0.00018 | 7.89850 | 8.42690 | 8.79450 | 8.98280 | 8.98870 |
| 0.00033 | 0.00032 | 7.90080 | 8.45920 | 8.80480 | 8.97850 | 8.97850 |
| 0.00033 | 0.00043 | 7.90070 | 8.47050 | 8.80950 | 8.97700 | 8.97700 |
| 0.00033 | 0.00059 | 7.89940 | 8.47830 | 8.81440 | 8.97600 | 8.97600 |
| 0.00033 | 0.00070 | 7.89820 | 8.48170 | 8.81720 | 8.97780 | 8.97780 |
| 0.00033 | 0.00082 | 7.89720 | 8.48420 | 8.81950 | 8.97840 | 8.97840 |
| 0.00033 | 0.00090 | 7.89720 | 8.48430 | 8.81960 | 8.97850 | 8.97850 |
| 0.00033 | 0.00102 | 7.89490 | 8.48850 | 8.82420 | 8.98310 | 8.97730 |
| 0.00033 | 0.00109 | 7.89480 | 8.48850 | 8.82420 | 8.98280 | 8.97580 |
| 0.00033 | 0.00114 | 7.89400 | 8.48980 | 8.82580 | 8.98380 | 8.97770 |
| 0.00033 | 0.00133 | 7.89270 | 8.49260 | 8.82910 | 8.98580 | 8.97840 |
|  |  |  |  |  |  |  |
|  | **Δδ [ppm]** | **0.00020** | **0.13610** | **0.05400** | **-0.01230** | **-0.03420** |

| **c(1) [M]** | **c(C_70_) [M]** | **Proton 9** | **Proton 11** | **Proton 8** | **Proton 10** | **Proton 6/7** |
| --- | --- | --- | --- | --- | --- | --- |
| 0.00033 | 0.00000 | 9.09860 | 9.11300 | 9.12430 | 9.16820 | 9.18690 |
| 0.00033 | 0.00010 | 9.08780 | 9.11450 | 9.12580 | 9.16210 | 9.18210 |
| 0.00033 | 0.00018 | 9.07970 | 9.11570 | 9.12710 | 9.15760 | 9.17910 |
| 0.00033 | 0.00032 | 9.07160 | 9.11690 | 9.12830 | 9.15310 | 9.17580 |
| 0.00033 | 0.00043 | 9.06890 | 9.11690 | 9.12820 | 9.15190 | 9.17460 |
| 0.00033 | 0.00059 | 9.06710 | 9.11650 | 9.12740 | 9.15140 | 9.17470 |
| 0.00033 | 0.00070 | 9.06640 | 9.11620 | 9.12670 | 9.15120 | 9.17450 |
| 0.00033 | 0.00082 | 9.06600 | 9.11600 | 9.12610 | 9.15140 | 9.17520 |
| 0.00033 | 0.00090 | 9.06600 | 9.11580 | 9.12620 | 9.15150 | 9.17510 |
| 0.00033 | 0.00102 | 9.06520 | 9.11510 | 9.12480 | 9.15180 | 9.17400 |
| 0.00033 | 0.00109 | 9.06530 | 9.11510 | 9.12470 | 9.15180 | 9.17400 |
| 0.00033 | 0.00114 | 9.06500 | 9.11490 | 9.12430 | 9.15190 | 9.17490 |
| 0.00033 | 0.00133 | 9.06500 | 9.11440 | 9.12360 | 9.15240 | 9.17400 |
|  |  |  |  |  |  |  |
|  | **Δδ [ppm]** | **-0.03360** | **0.00140** | **-0.00070** | **-0.01580** | **-0.01290** |

The ^1^H chemical shifts of all protons in the obtained spectra were fitted using the Bindfitv0.5 software ([http://supramolecular.org](http://supramolecular.org/)) with the theoretical model H:G = 1:2. Above, the protons are named in the order of appearance in the spectrum from upfield to downfield.


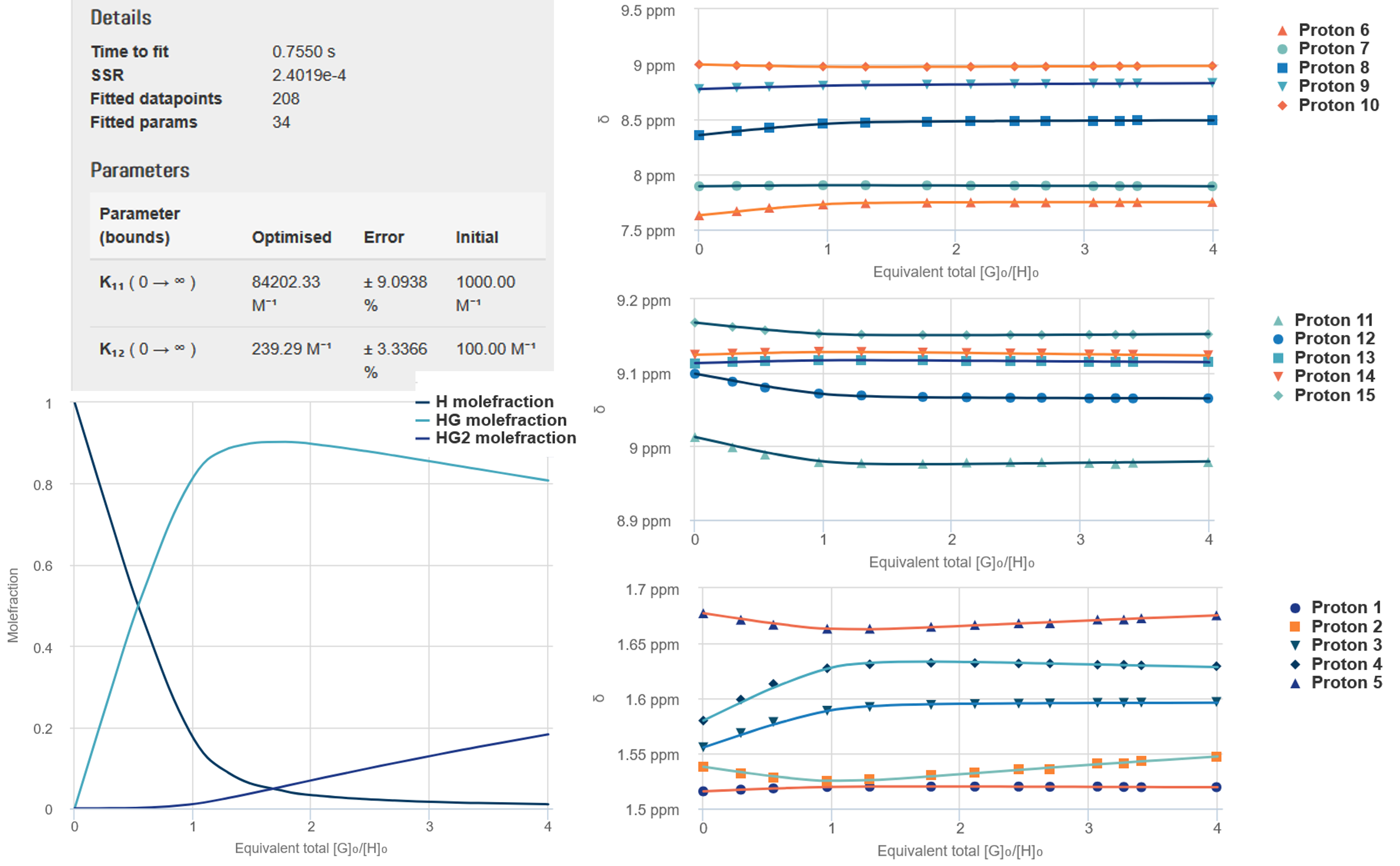


**Figure S22.** Fitting results (top left), molar fractions plot (bottom left), and ^1^H chemical shifts with fits (right) obtained from BindFit v.05 for the titration of **1** with C_70_.


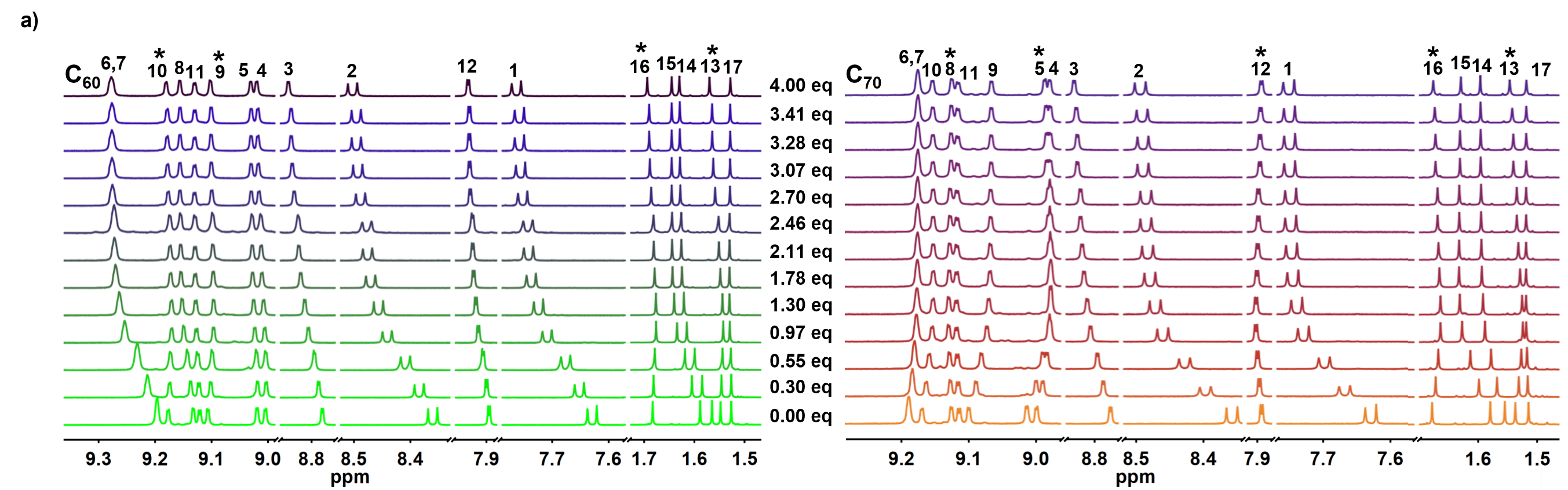


**Figure S23.** Stacked ^1^H NMR spectra of different ratios of **1** and C_70_ at constant concentration of **1** (toluene-d_8_, 295 K); protons labelled with numbers, signals displaying a reversal in chemical shift displacement marked with *****.

# **9. CD titration experiments**

*Procedure for C_60_ titration*: 3 mL of a solution of **1** (*c*_Host_ = 10.88 × 10^-6^ M, toluene) was treated with distinct aliquots of a solution of C_60_ (*c*_Guest_ = 3.26 × 10^-4^ M, toluene), thoroughly mixed, and its CD spectrum recorded. The binding constant *K*_a_ was determined from the measured Δε-values in the wavelength region of 367–362 nm. The quality of the obtained data rapidly declined after addition of more than four equivalents of C_60_, likely due to the absorption of the fullerene in the investigated region of wavelengths.

**Table S16.** Δε values determined from CD measurements during titration of **1** with C_60_.

| **c(1) [M]** | **c(C_60_) [M]** | **367 nm** | **366 nm** | **365 nm** | **364 nm** | **363 nm** | **362 nm** |
| --- | --- | --- | --- | --- | --- | --- | --- |
| 1.09E-05 | 0.00 | -363.79 | -380.74 | -391.40 | -400.09 | -403.15 | -403.44 |
| 1.09E-05 | 2.72E-06 | -362.49 | -377.17 | -388.29 | -395.99 | -399.46 | -398.24 |
| 1.09E-05 | 5.44E-06 | -360.08 | -374.98 | -385.89 | -392.36 | -394.59 | -394.66 |
| 1.09E-05 | 8.16E-06 | -358.20 | -373.13 | -382.92 | -390.05 | -393.02 | -390.58 |
| 1.09E-05 | 1.09E-05 | -356.99 | -370.58 | -381.00 | -386.09 | -387.87 | -385.12 |
| 1.09E-05 | 1.36E-05 | -355.22 | -368.66 | -378.55 | -383.98 | -385.54 | -381.88 |
| 1.09E-05 | 1.63E-05 | -354.12 | -366.94 | -376.62 | -380.98 | -382.39 | -380.24 |
| 1.09E-05 | 1.90E-05 | -354.16 | -366.08 | -373.16 | -378.39 | -381.09 | -375.63 |
| 1.09E-05 | 2.18E-05 | -352.21 | -363.93 | -371.84 | -377.31 | -378.35 | -373.49 |
| 1.09E-05 | 2.45E-05 | -351.17 | -363.35 | -370.38 | -375.43 | -374.91 | -372.30 |
| 1.09E-05 | 2.72E-05 | -349.65 | -361.28 | -369.86 | -373.08 | -373.17 | -369.52 |
| 1.09E-05 | 2.99E-05 | -348.96 | -359.79 | -367.68 | -370.65 | -370.80 | -365.76 |
| 1.09E-05 | 3.26E-05 | -348.91 | -359.22 | -365.85 | -368.33 | -369.49 | -363.55 |
| 1.09E-05 | 3.54E-05 | -347.63 | -357.85 | -363.56 | -368.58 | -367.97 | -361.39 |
| 1.09E-05 | 3.81E-05 | -345.24 | -357.08 | -363.05 | -366.78 | -365.69 | -357.95 |
| 1.09E-05 | 4.08E-05 | -345.57 | -355.65 | -360.31 | -364.53 | -364.89 | -357.47 |
| 1.09E-05 | 4.35E-05 | -346.00 | -356.11 | -361.00 | -364.35 | -363.50 | -358.09 |

The above displayed data was fitted with BindFit v0.5. The fits, molar fractions and determined binding value are displayed below.


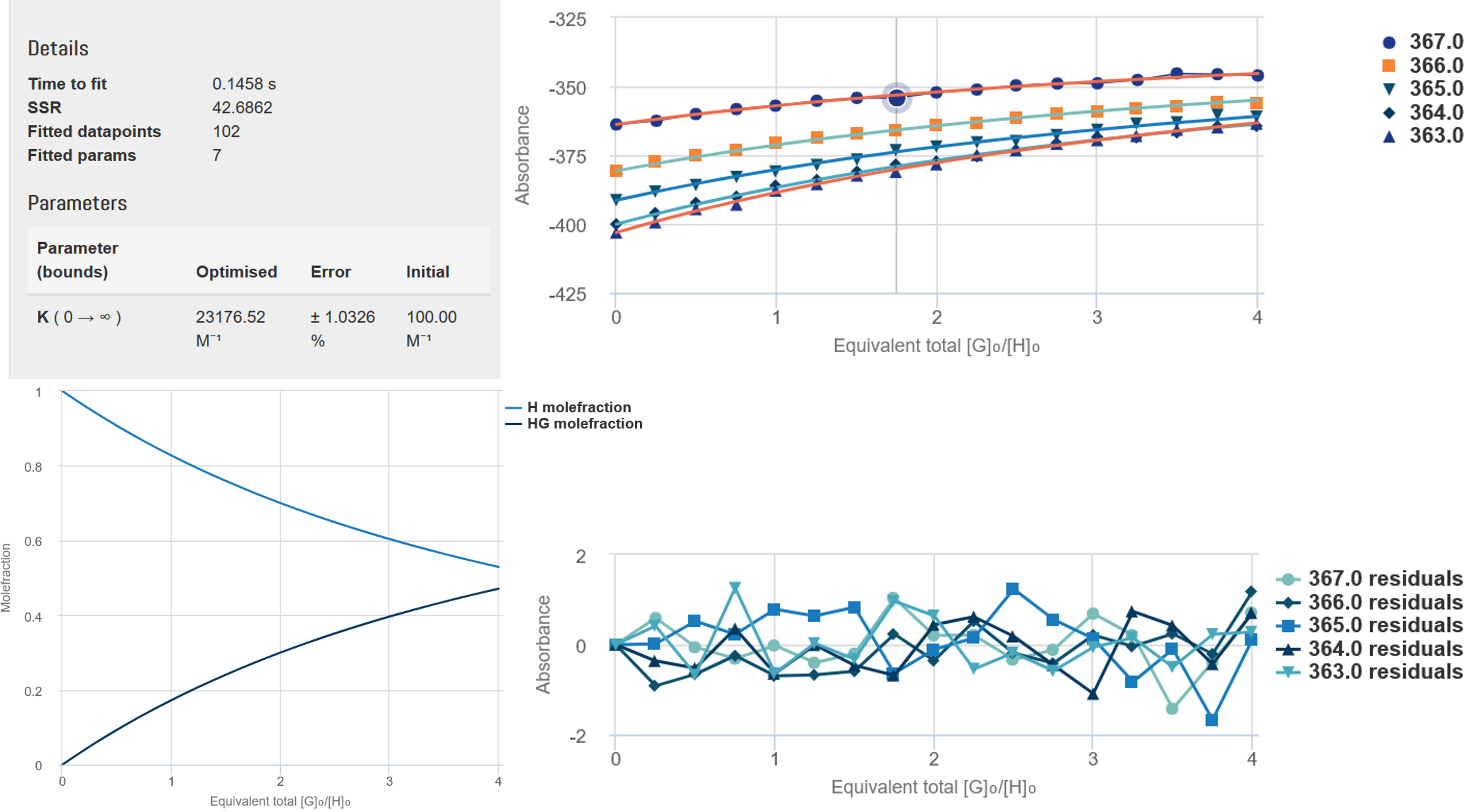


**Figure S24.** Fitting results (top left), molar fractions plot (bottom left), chemical shifts with fits (top right), and residuals (bottom left) obtained from BindFit v.05 for the titration of **1** with C_60_.

*Procedure for C_70_ titration*: 3 mL of a solution of **1** (*c*_Host_ = 9.64 × 10^-6^ M, toluene) was treated with distinct aliquots of a solution of C_70_ (*c*_Guest_ = 2.89 × 10^-4^ M, toluene), thoroughly mixed, and its CD spectrum recorded. The binding constant *K*_a_ was determined from the measured Δε-values in the wavelength region of 367–362 nm. The quality of the obtained data rapidly declined after addition of more than two equivalents of C_70_, likely due to the absorption of the fullerene in the investigated region of wavelengths.

**Table S17.** Δε values determined from CD measurements during titration of **1** with C_70_.

| **c(1) [M]** | **c(C_70_) [M]** | **367 nm** | **366 nm** | **365 nm** | **364 nm** | **363 nm** | **362 nm** |
| --- | --- | --- | --- | --- | --- | --- | --- |
| 9.64E-06 | 0.00 | -357.41 | -372.49 | -383.78 | -391.16 | -394.81 | -393.16 |
| 9.64E-06 | 2.41E-06 | -353.43 | -367.57 | -375.44 | -381.70 | -383.58 | -380.41 |
| 9.64E-06 | 4.82E-06 | -350.35 | -360.47 | -368.90 | -372.95 | -372.92 | -368.25 |
| 9.64E-06 | 7.23E-06 | -346.47 | -355.58 | -361.78 | -364.61 | -363.99 | -359.63 |
| 9.64E-06 | 9.64E-06 | -343.50 | -352.42 | -357.69 | -358.49 | -356.75 | -351.25 |
| 9.64E-06 | 1.20E-05 | -341.66 | -349.08 | -352.10 | -354.14 | -350.71 | -344.26 |
| 9.64E-06 | 1.45E-05 | -339.34 | -345.29 | -348.88 | -347.87 | -344.64 | -339.50 |
| 9.64E-06 | 1.69E-05 | -336.04 | -344.40 | -345.33 | -344.28 | -340.60 | -333.73 |
| 9.64E-06 | 1.93E-05 | -336.50 | -341.81 | -341.31 | -340.32 | -336.35 | -325.68 |


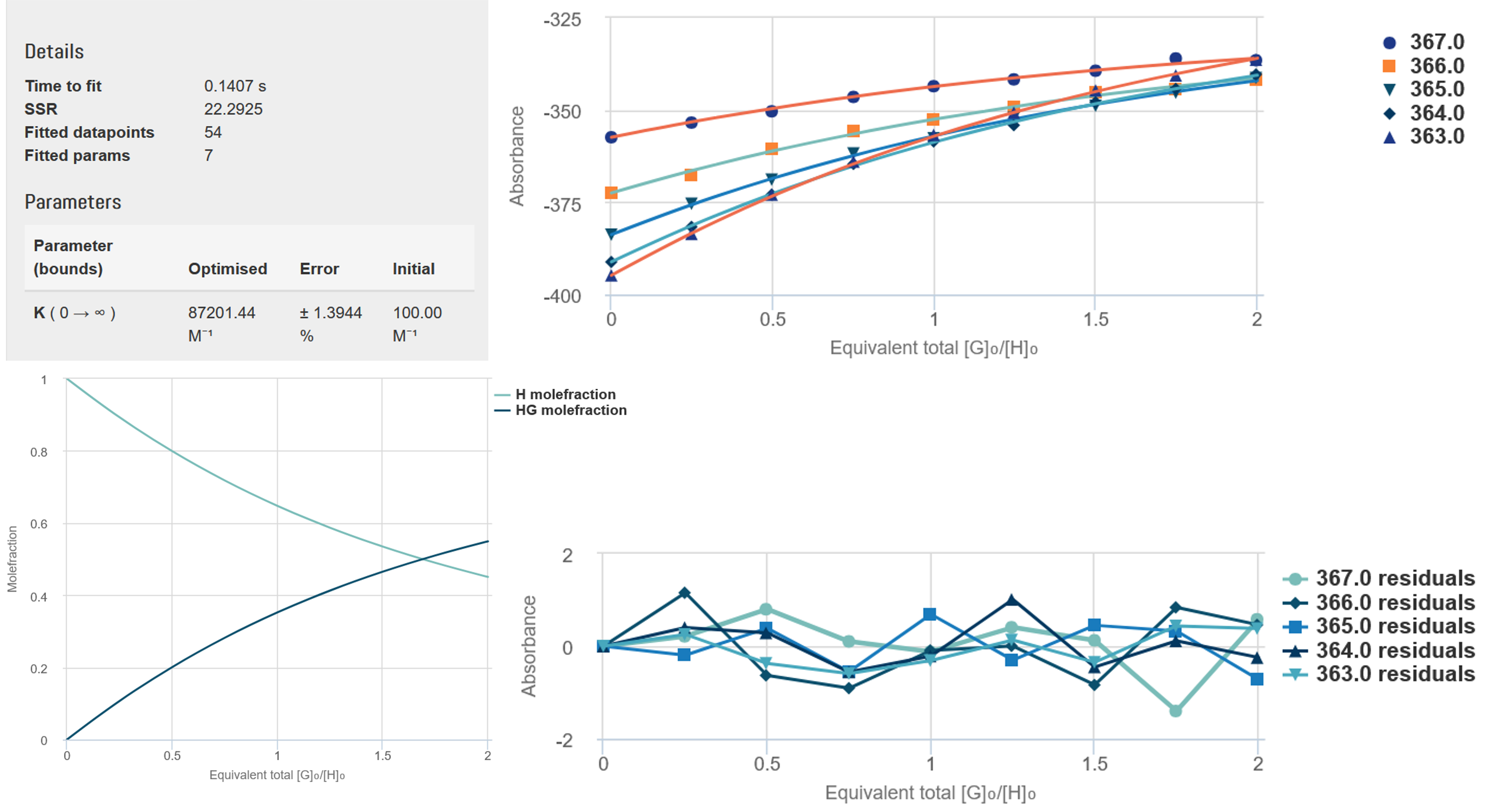


**Figure S25.** Fitting results (top left), molar fractions plot (bottom left), chemical shifts with fits (top right), and residuals (bottom left) obtained from BindFit v.05 for the titration of **1** with C_70_.

# **10. Comparison of binding properties with reported molecules**

**Table S18.** Comparison of the interaction of literature reported nanographenes with fullerenes C_60_ and C_70_ and the results of this work.

| **Structure** | **Binding mode** | ***K*_a_** | **DOI** |
| --- | --- | --- | --- |
|  | 1:1 | 18.5 ± 0.3 M^-1^ for C_60_  53.1 ± 2.4 M^-1^ for C_70_ | 10.1055/s-0041-1722848 |
|  | 1:1 | 94.7 ± 0.9 M^-1^ for C_60_  154.3 ± 4.1 M^-1^ for C_70_ | 10.1021/acs.orglett.5c04019 |
|  | 1:1 | 61 ± 1 M^-1^ for C_60_  400 ± 17 M^-1^ for C_70_ | 10.1039/D4QO02071E |
|  | 1:1 | 112 ± 0.4 M^-1^ for C_60_  121 ± 3 M^-1^ for C_70_ | 10.1021/jacs.4c09185 |
|  | 1:1 | 388 ± 1.4 M^-1^ for C_60_  579.2 ± 2.8 M^-1^ for C_70_ | 10.1073/pnas.2426059122 |
|  | 1:1 | 1141±17 M^-1^ for C_60_ | 10.1021/jacs.3c10122 |
| 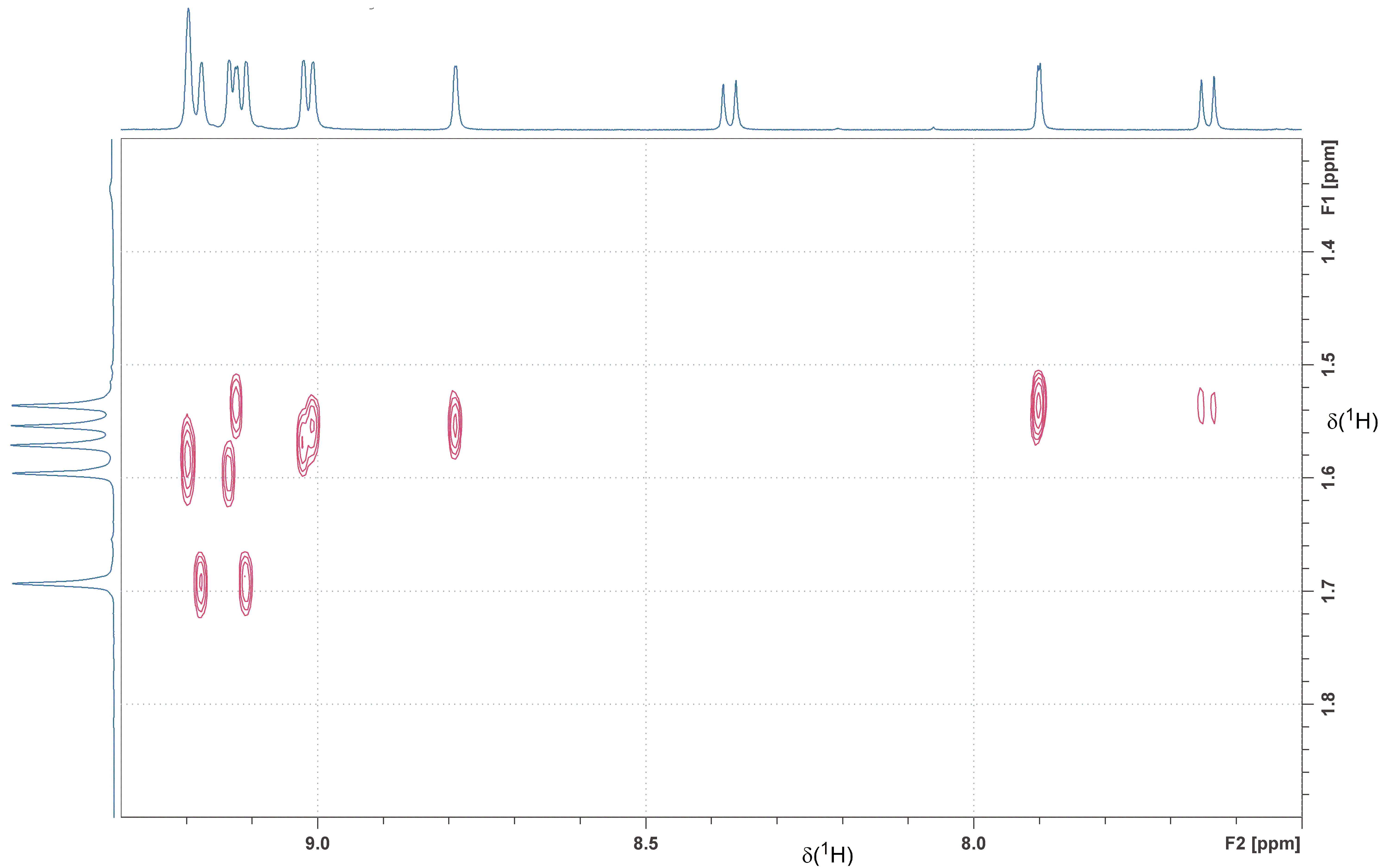  | 1:1 | 1170 ± 2.17 M^-1^  all for C_60_ | 10.1002/anie.202112834 |
|  | 1:2 | *K*_11_ = 1692 ± 2.92 M^-1^  *K*_12_ = 1157 ± 12.7 M^-1^ |  |
|  | 2:1 | *K*_11_ = 1710 ± 8.89 M^-1^  *K*_12_ = 3173 ± 13.9 M^-^ |  |
|  | 1:1 | 3900 ± 100 M^-1^ for C_60_  30000 ± 3000 M^-1^ for C_70_ | 10.1021/jacs.4c09224 |
|  | 1:1 | 9500 M^-1^ for C_60_  37200 M^-1^ for C_70_ | 10.1002/anie.202400172 |
|  | 1:1 | 15400 M^-1^ for C_60_  41000 M^-1^ for C_70_ | 10.1002/anie.202516881 |
|  | 1:1 | 23200 ± 200 M^-1^ for C_60_  87200 ± 100 M^-1^ for C_70_ | This Work |
|  | 1:2 | *K*_11_ =21000 ± 3600 M^-1^  *K*_12_ =180 ± 10 M^-1^ for C_60_  *K*_11_ = 84200 ± 7700 M^-1^  *K*_12_ = 240 ± 10 M^-1^ for C_70_ |  |

# **11. ^1^H and ^13^C NMR spectra and HR MS spectra**


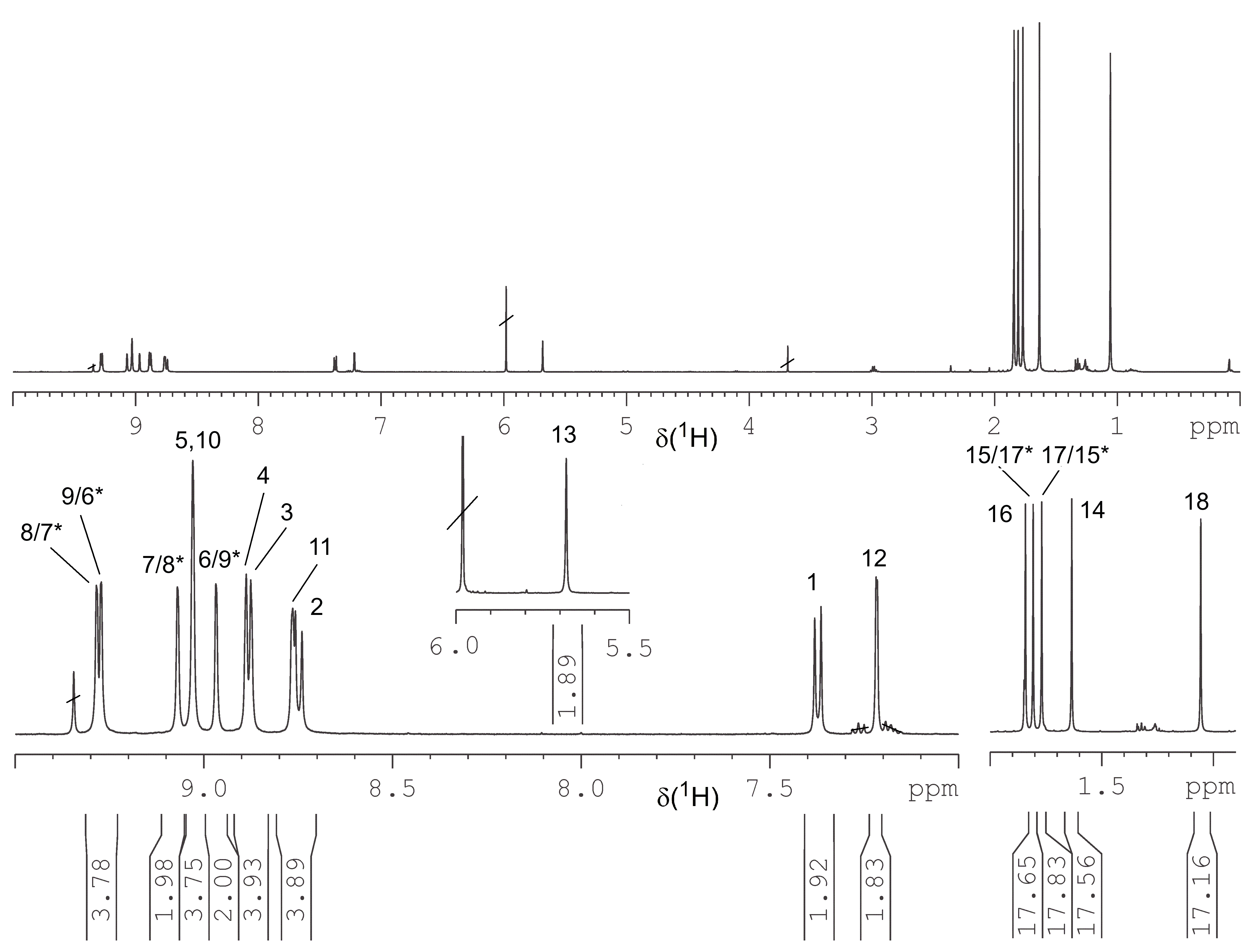


**Figure S26.** ^1^H NMR spectrum of **2** (500 MHz, C_2_D_2_Cl_4_) and enlarged regions. There are two alternative assignments for H_6_ – H_9_, H_15_ and H_17_ (with or without *).


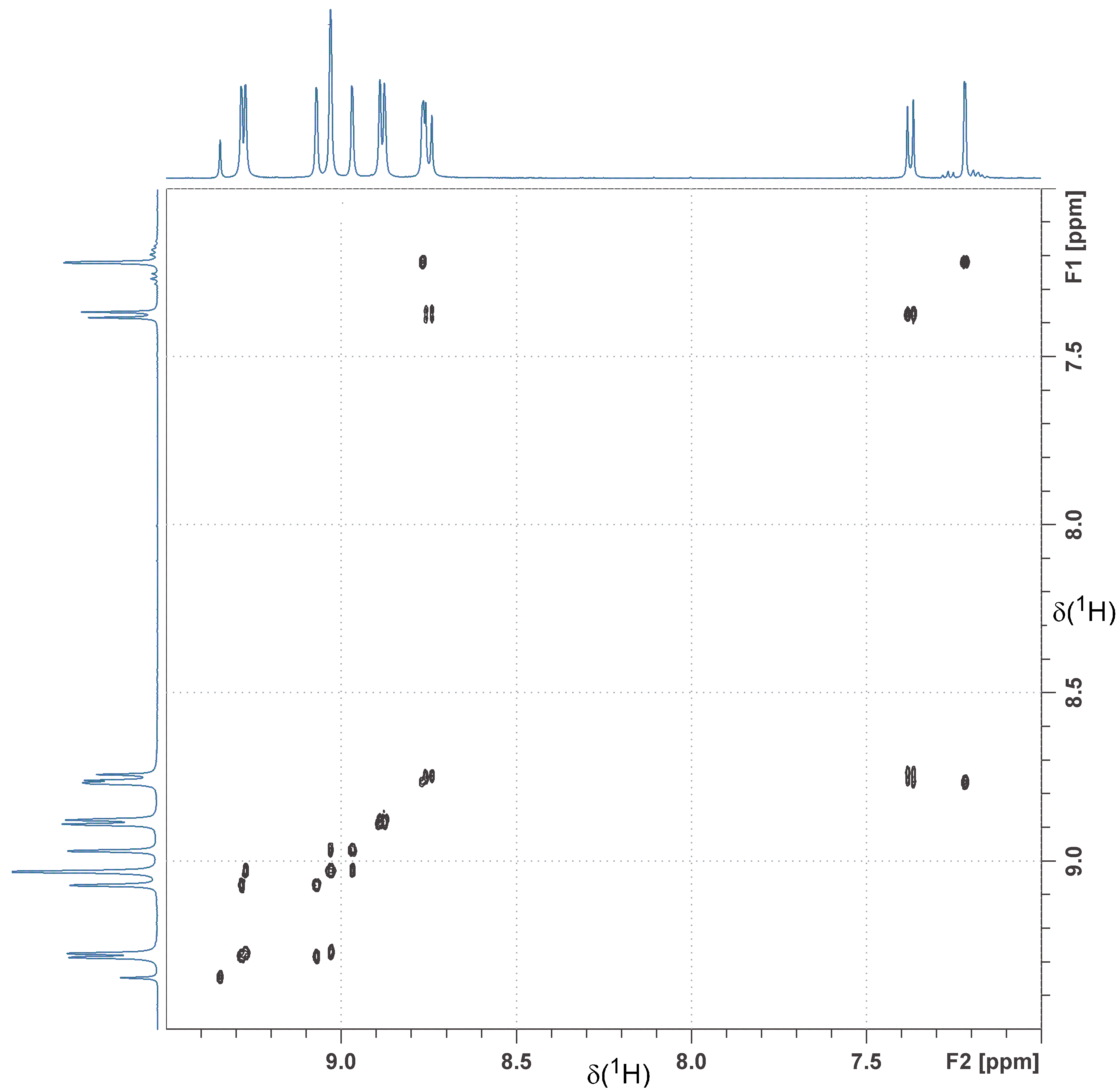


**Figure S27.** Long-range ^1^H-^1^H COSY spectrum (region of aromatic protons) of **2** (500 MHz, C_2_D_2_Cl_4_).


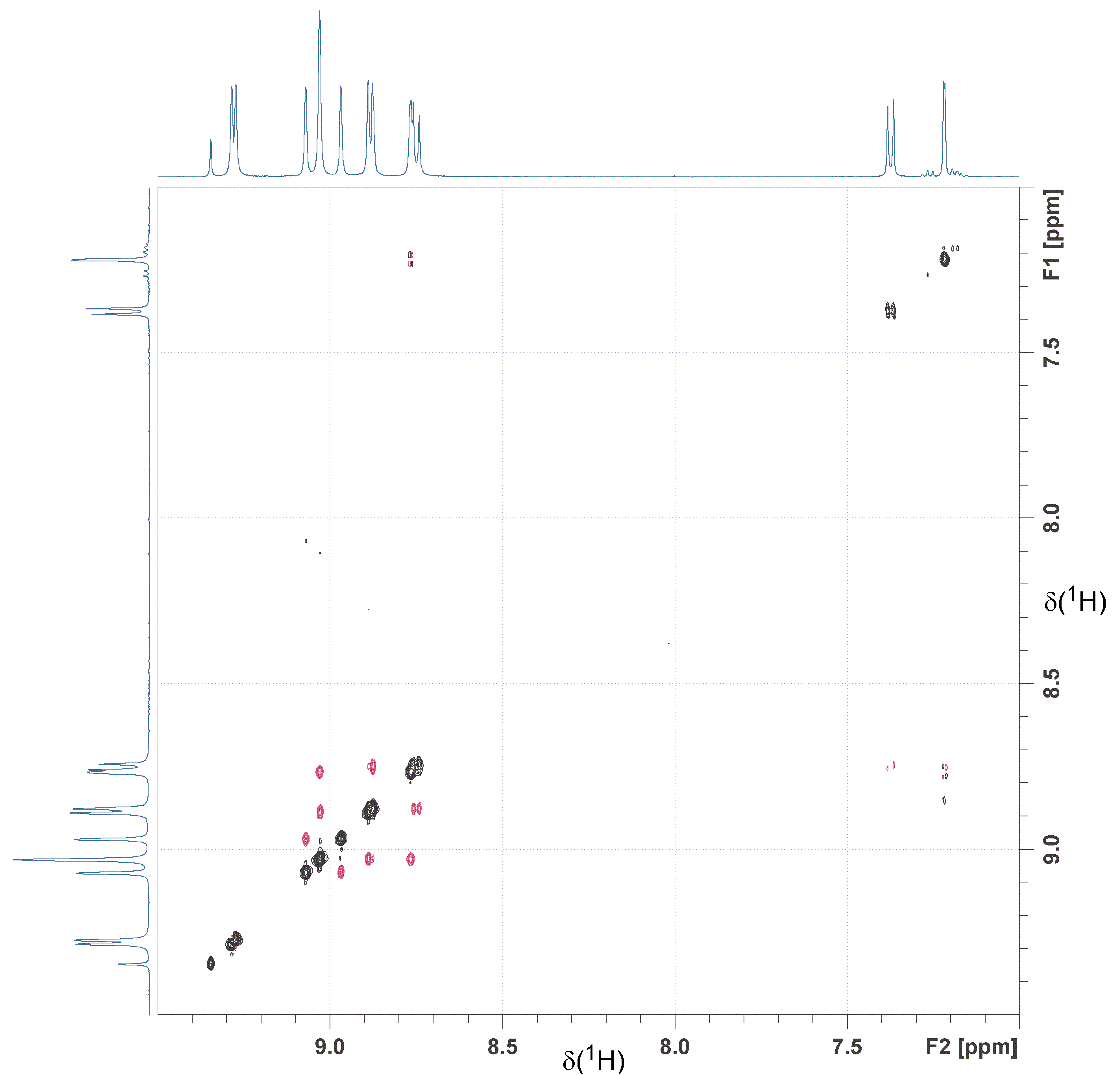


**Figure S28.** ^1^H-^1^H ROESY spectrum (region of aromatic protons) of **2** (500 MHz, C_2_D_2_Cl_4_).


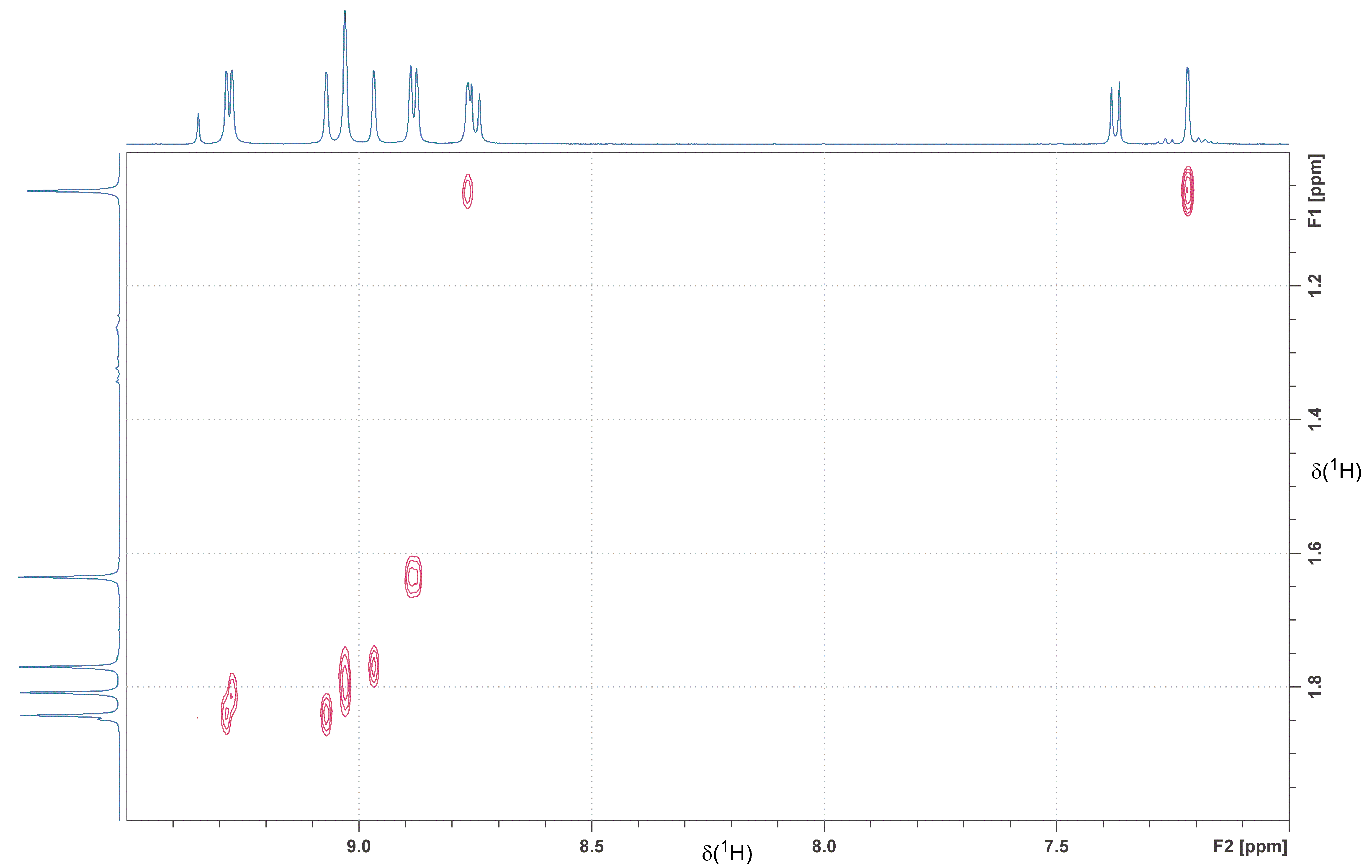


**Figure S29.** ^1^H-^1^H ROESY spectrum (correlations of methyl and aromatic protons) of **2** (500 MHz, C_2_D_2_Cl_4_).


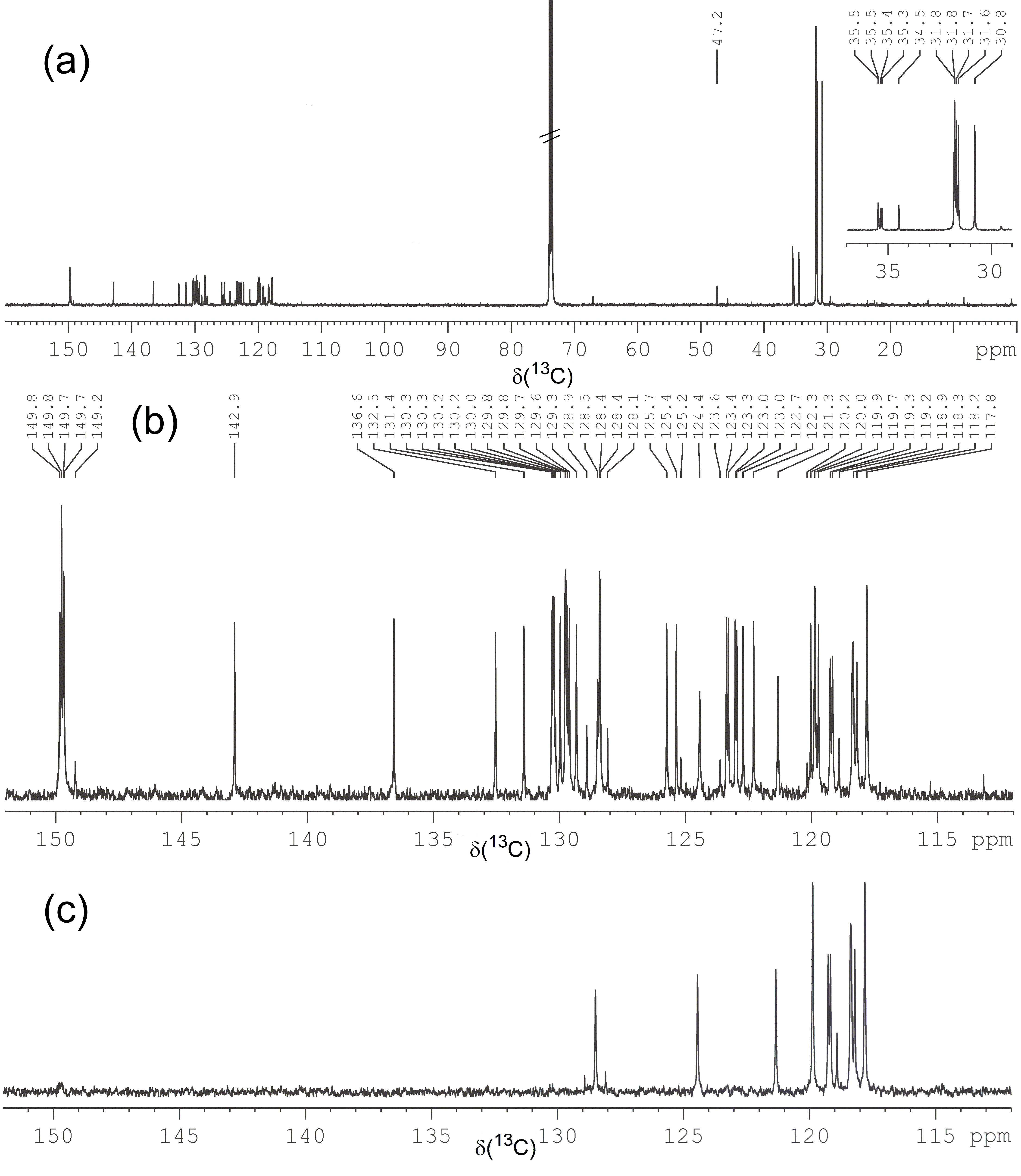


**Figure S30.** ^13^C NMR spectrum of **2** (125 MHz, C_2_D_2_Cl_4_) (a) and region of aromatic carbons (b). (c) depicts the DEPT135 spectrum (region of aromatic CH carbons).


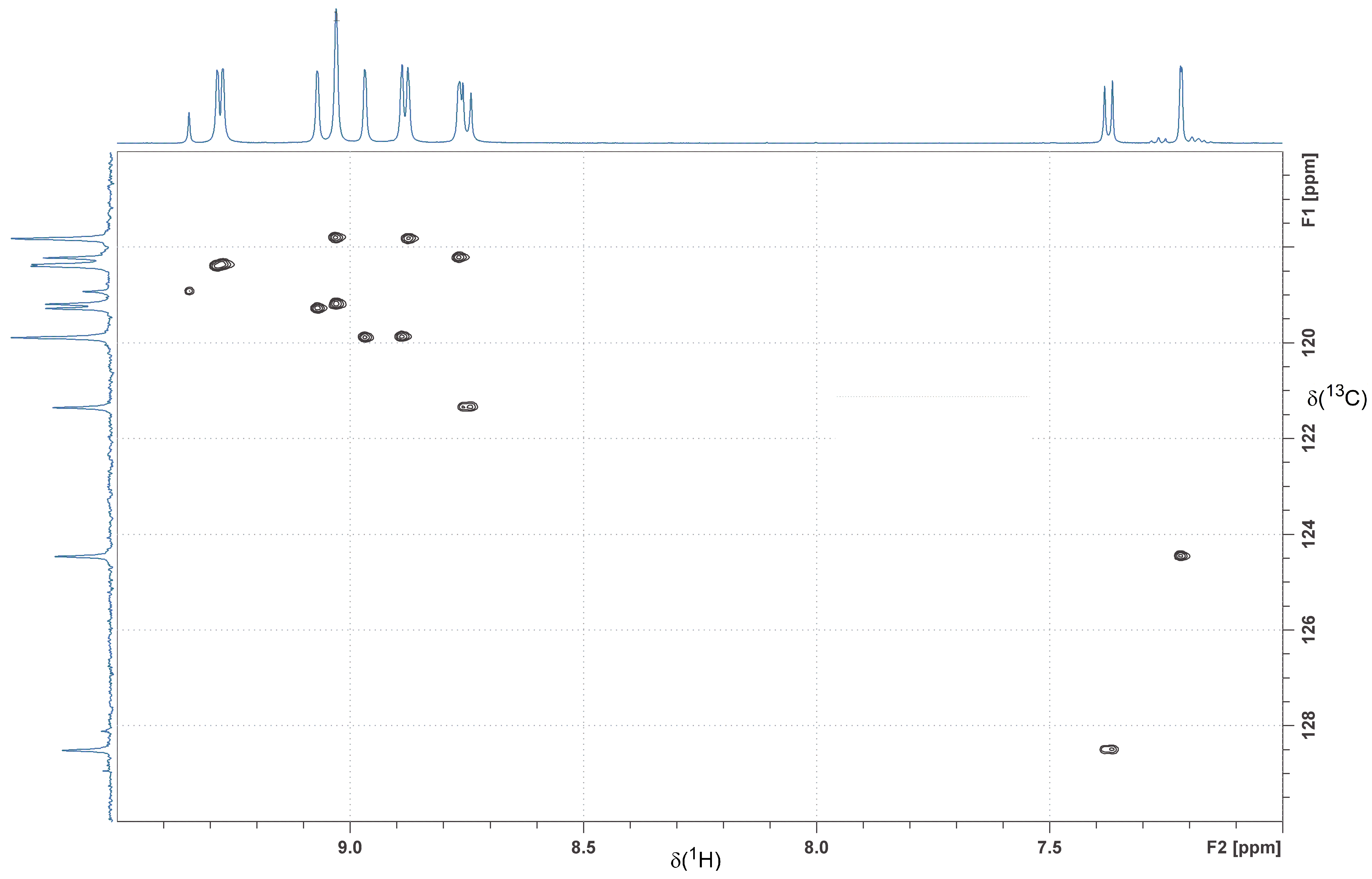


**Figure S31.** ^1^H-^13^C HSQC spectrum (region of aromatic CH carbons) of **2** (125 MHz, C_2_D_2_Cl_4_). The F1 axis shows the DEPT135 spectrum.


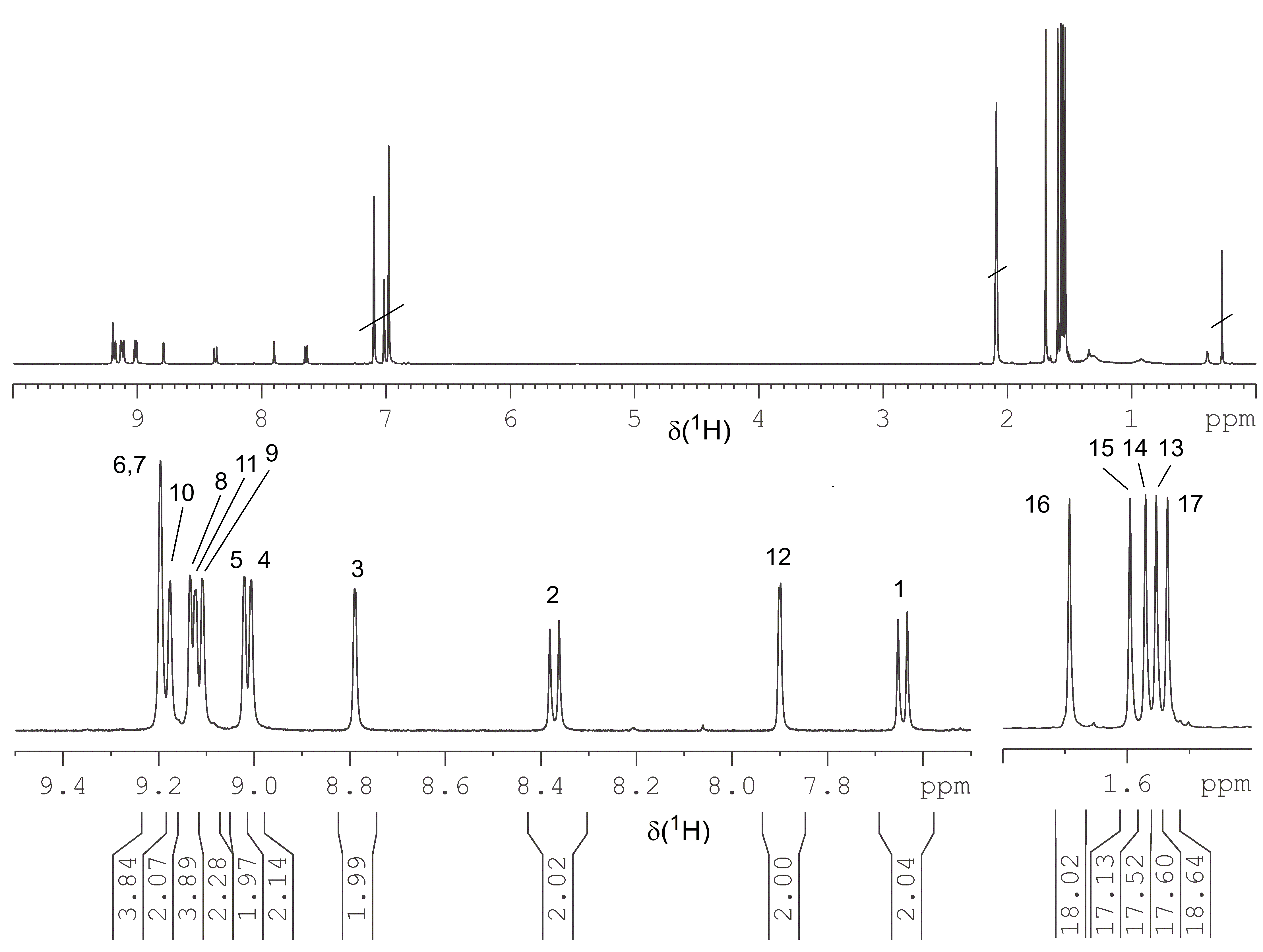


**Figure S32.** ^1^H NMR spectrum of **1** (500 MHz, toluene-d_8_) and enlarged regions.


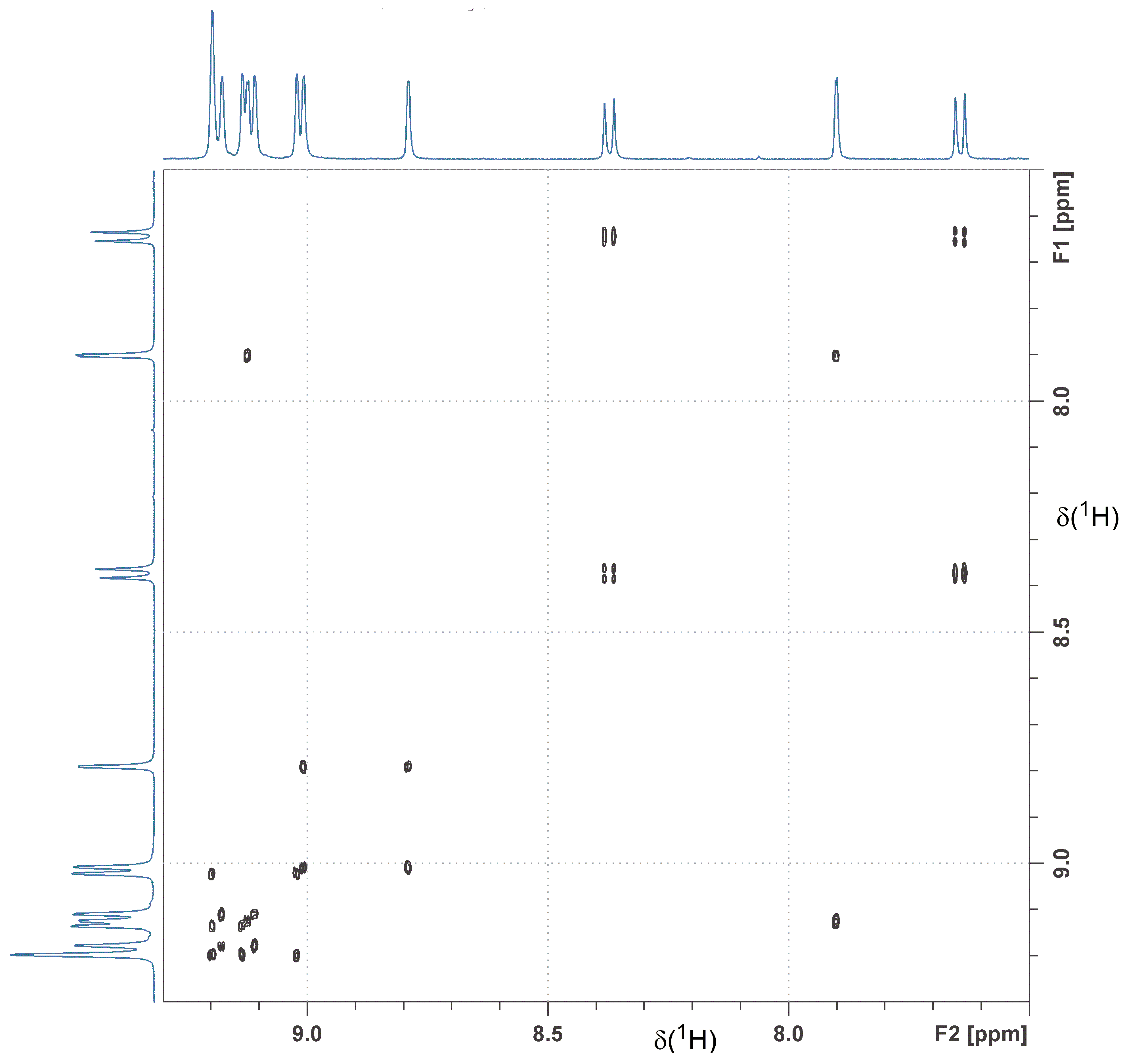


**Figure S33.** Long-range ^1^H-^1^H COSY spectrum (region of aromatic protons) of **1** (500 MHz, toluene-d_8_).


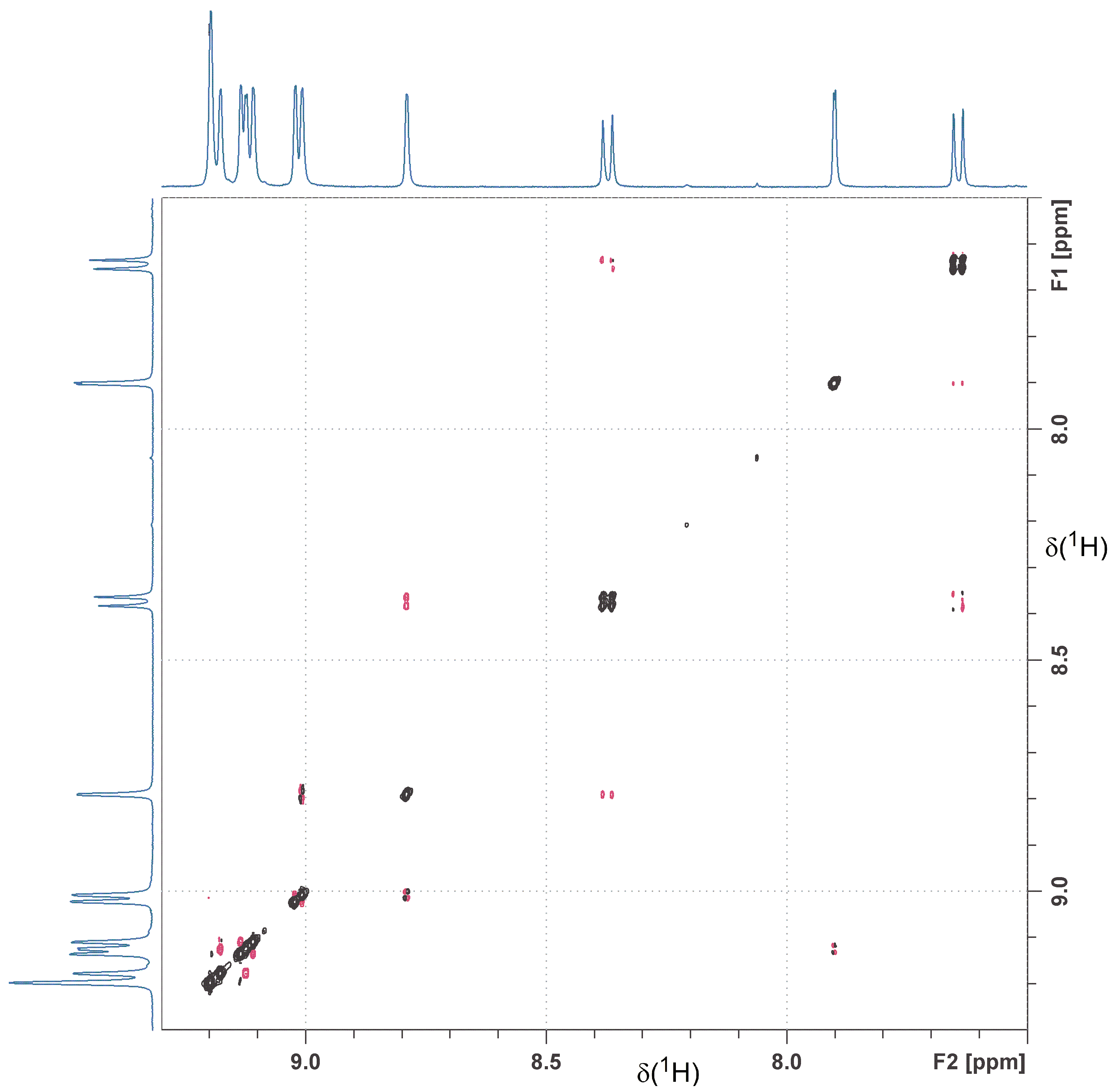


**Figure S34.** ^1^H-^1^H ROESY spectrum (region of aromatic protons) of **1** (500 MHz, toluene-d_8_).


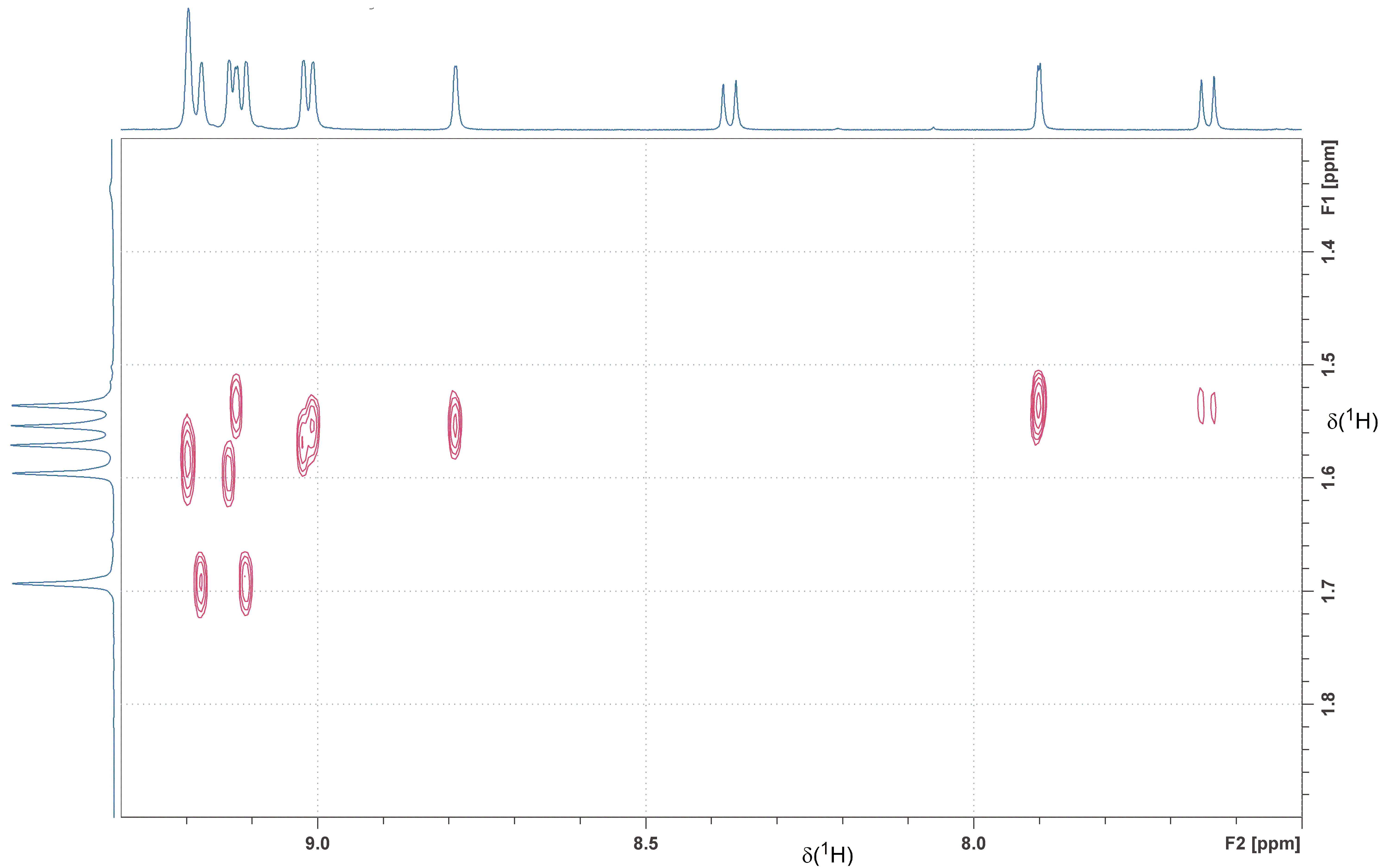


**Figure S35.** ^1^H-^1^H ROESY spectrum (correlations of methyl and aromatic protons) of **1** (500 MHz, toluene-d_8_).


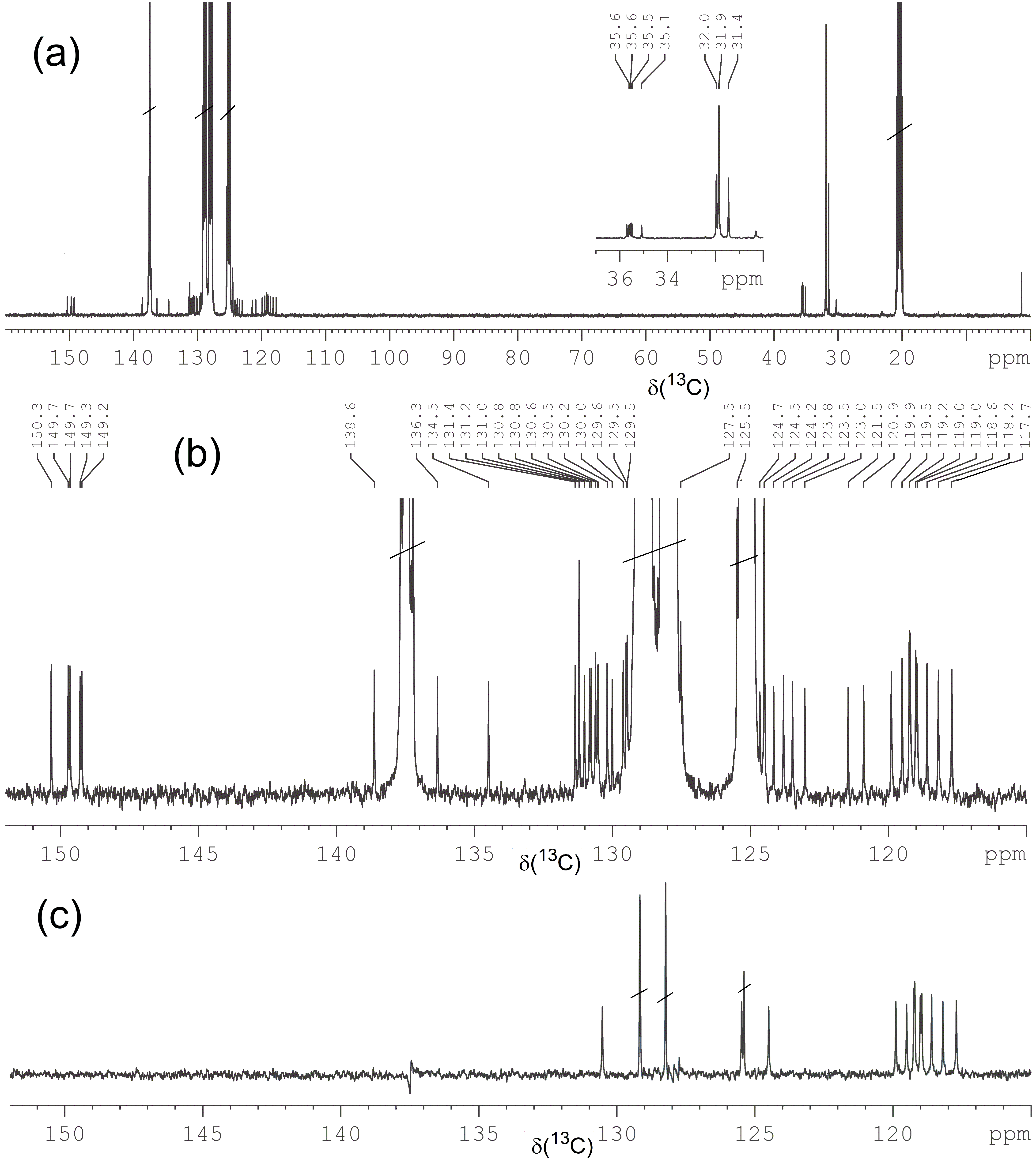


**Figure S36.** ^13^C NMR spectrum of **1** (125 MHz, toluene-d_8_) (a) and region of aromatic carbons (b). (c) depicts the DEPT135 spectrum (region of aromatic CH carbons).


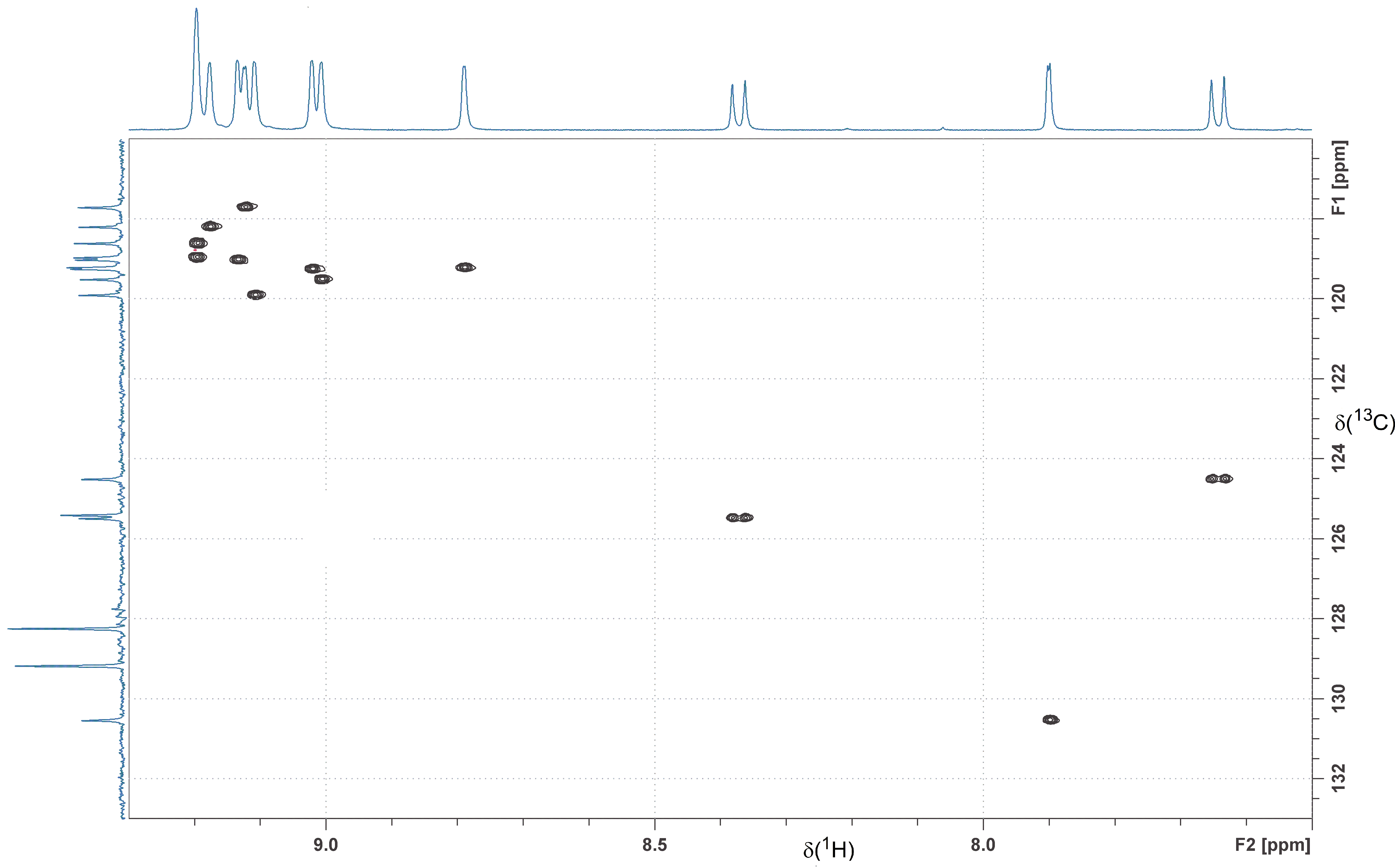


**Figure S37.** ^1^H-^13^C HSQC spectrum (region of aromatic CH carbons) of **1** (125 MHz, toluene-d_8_). The F1 axis shows the DEPT135 spectrum.


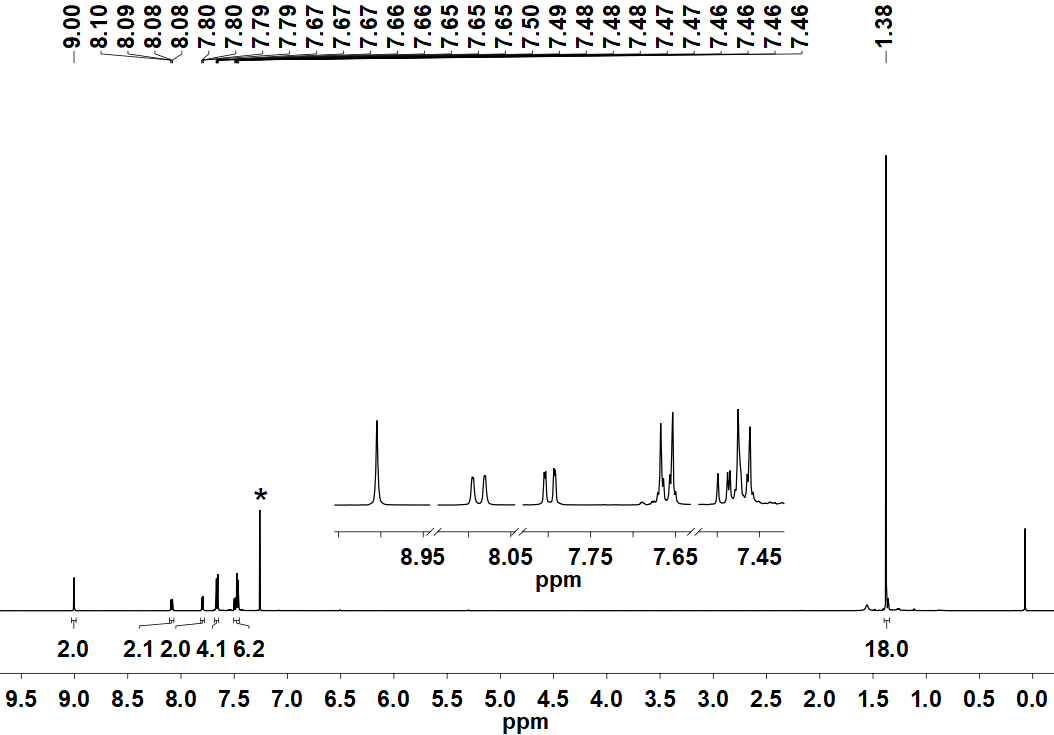


**Figure S38.** ^1^H NMR spectrum of compound **3** (600 MHz, CDCl_3_); solvent residue marked with *.


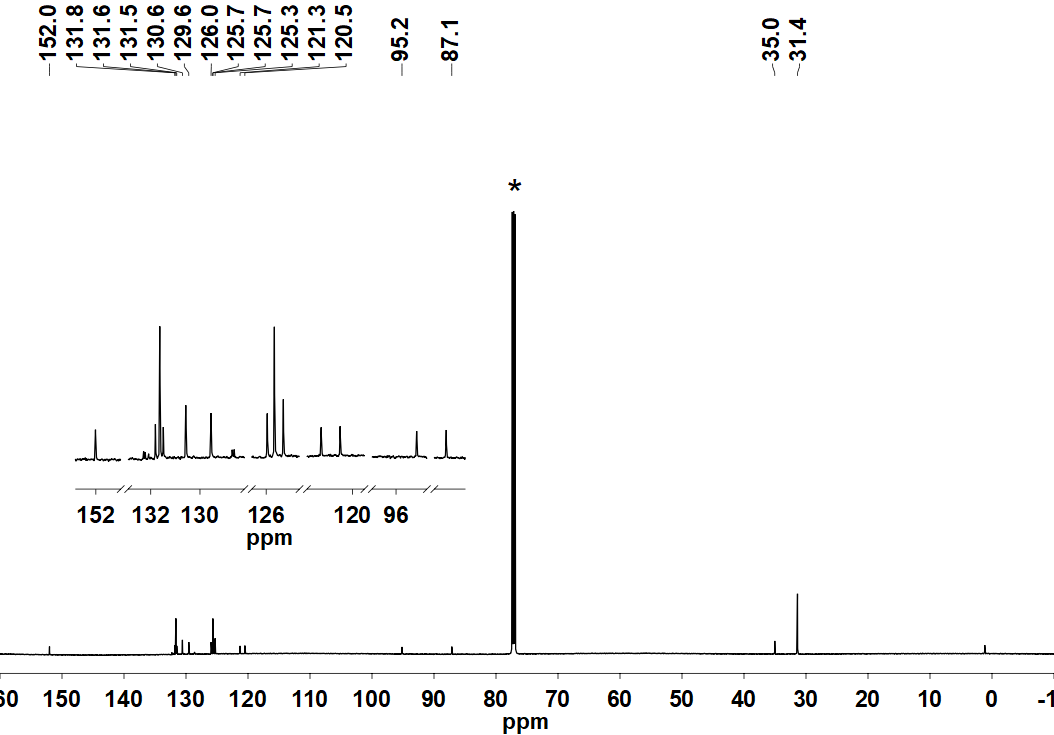


**Figure S39.** ^13^C NMR spectrum of compound **3** (150 MHz, CDCl_3_); solvent residue marked with *.


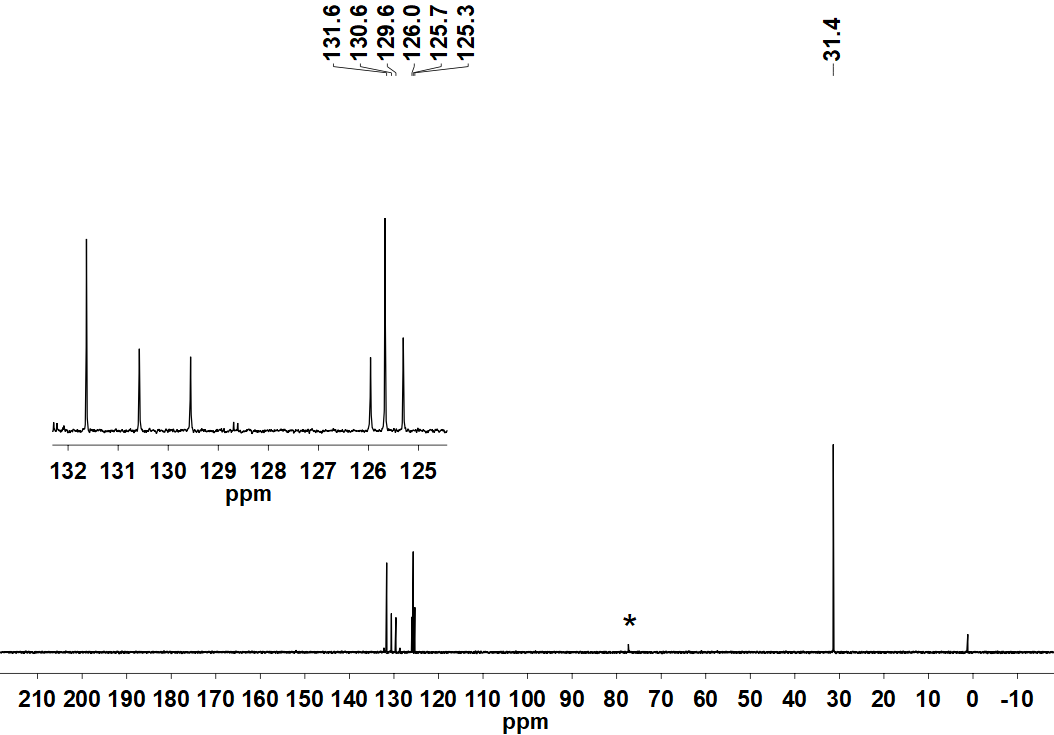


**Figure S40.** ^13^C DEPT NMR spectrum of compound **3** (150 MHz, CDCl_3_); solvent residue marked with *.


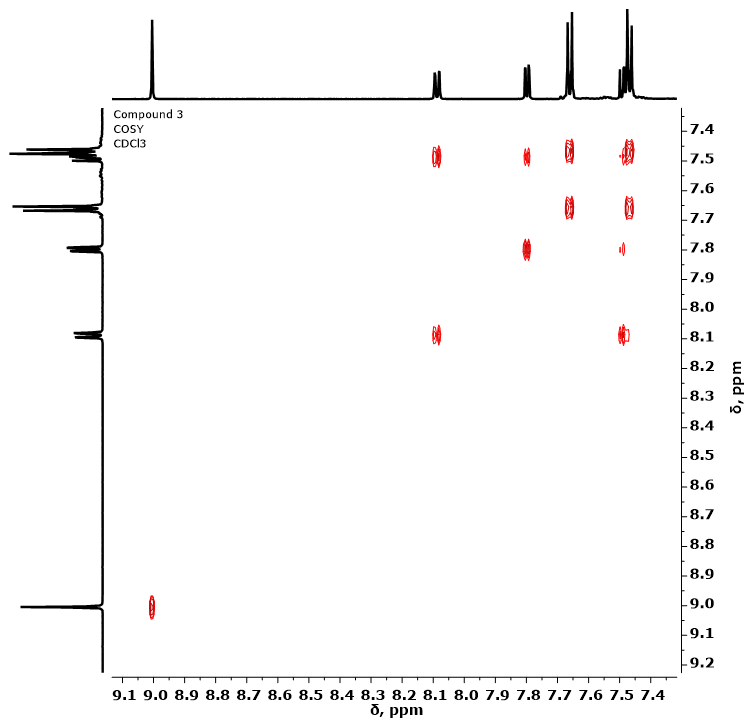


**Figure S41.** ^1^H-^1^H COSY NMR spectrum of compound **3** (600 MHz, CDCl_3_).


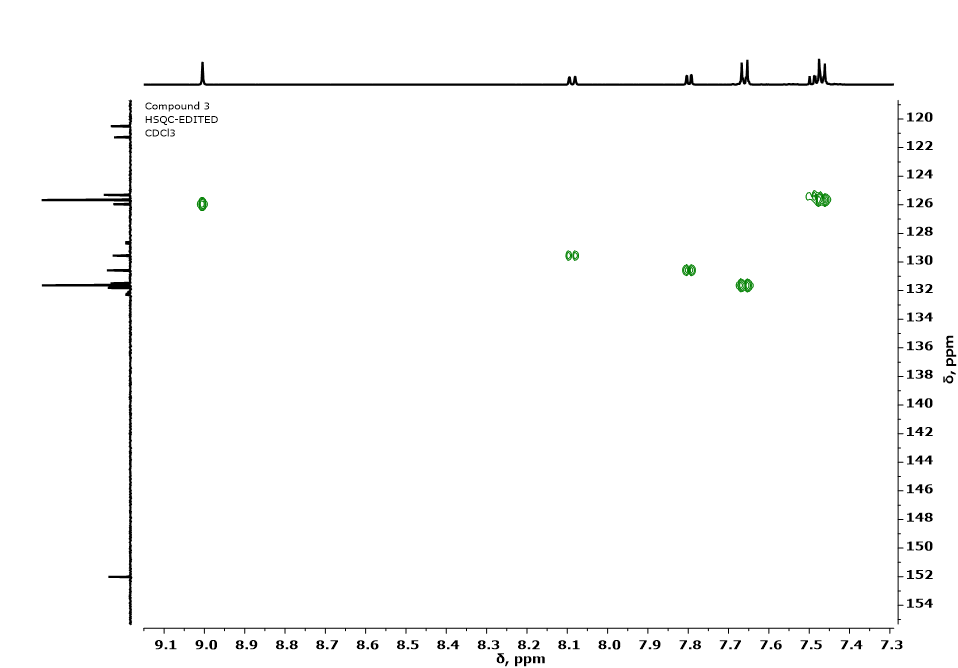


**Figure S42.** ^1^H-^13^C HSQC NMR spectrum of compound **3** (150 MHz, CDCl_3_).


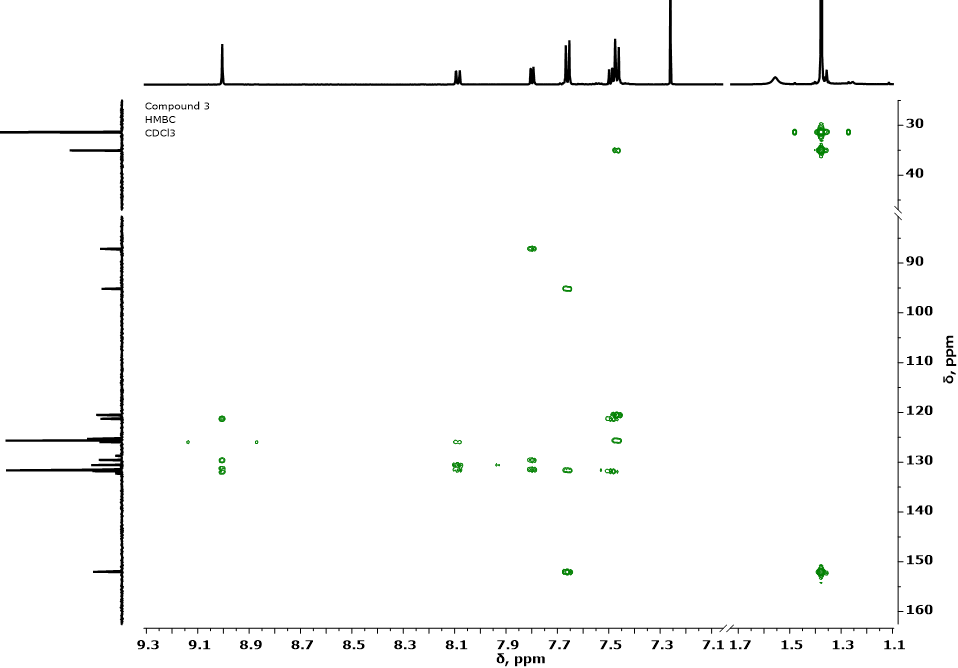


**Figure S43.** ^1^H-^13^C HMBC NMR spectrum of compound **3** (150 MHz, CDCl_3_).


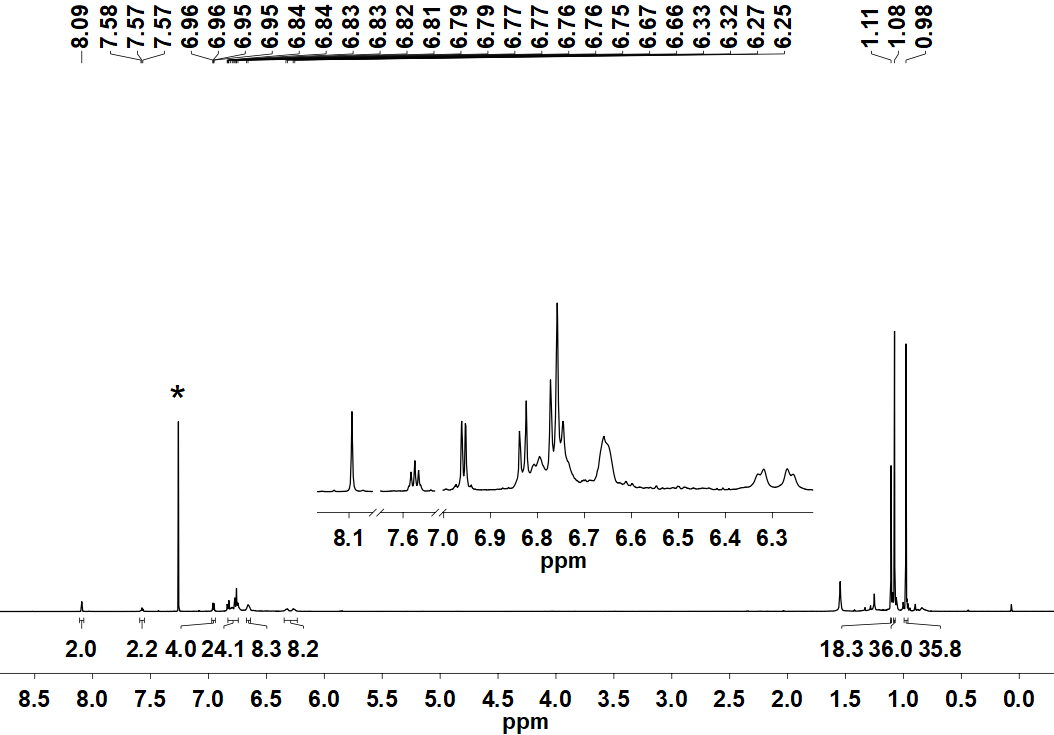


**Figure S44.** ^1^H NMR spectrum of compound **4** (600 MHz, CDCl_3_); solvent residue marked with *.


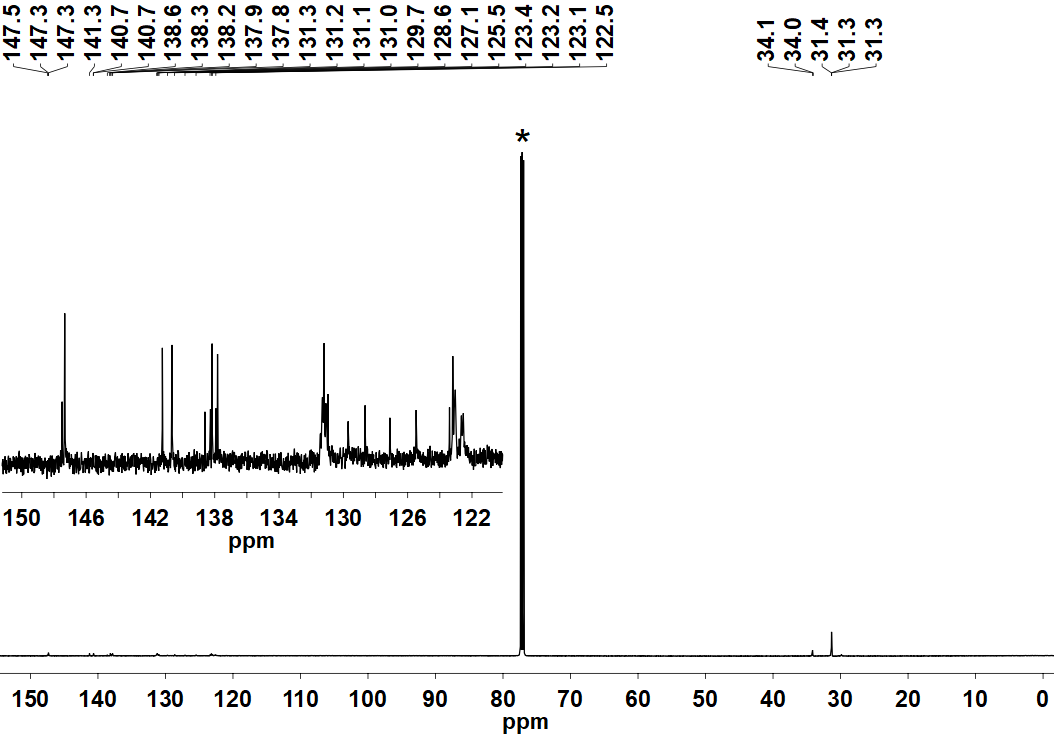


**Figure S45.** ^13^C NMR spectrum of compound **4** (150 MHz, CDCl_3_); solvent residue marked with *.


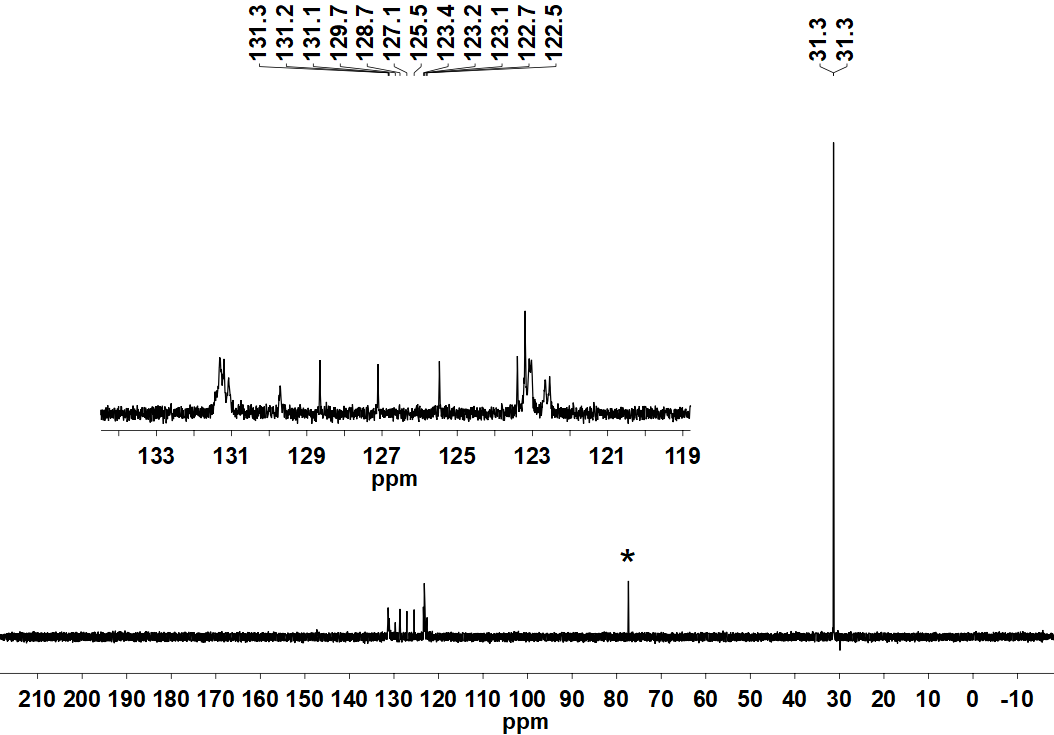


**Figure S46.** ^13^C DEPT NMR spectrum of compound **4** (150 MHz, CDCl_3_); solvent residue marked with *.


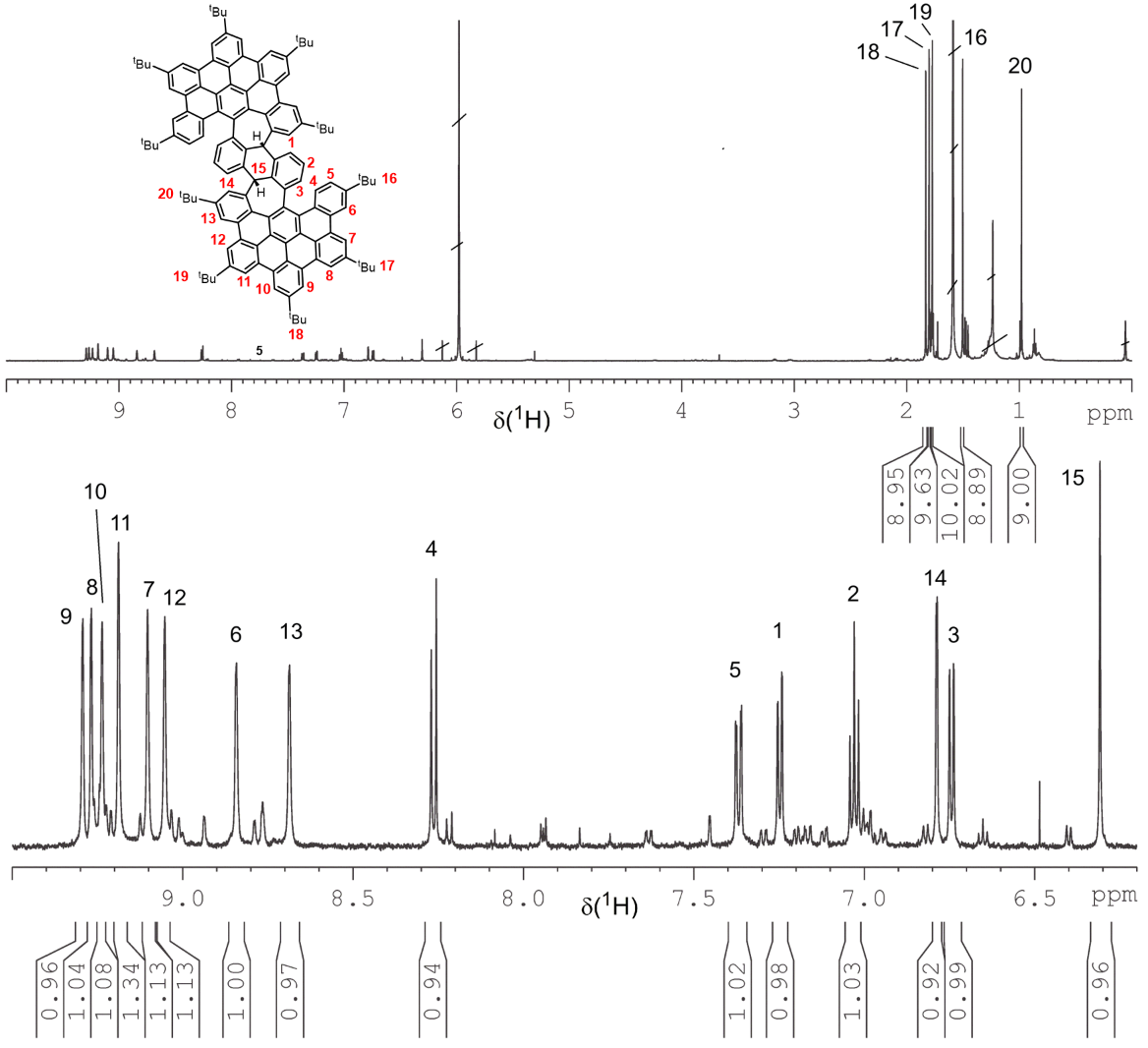


**Figure S47.** ^1^H NMR spectrum of **5** (600 MHz, C_2_D_2_Cl_4_) and enlarged region. Note: Compound **5** was specifically isolated at low temperature (−78 °C) to gain insight into the reaction pathway. The structure corresponds to an intermediate during the synthesis of compound **2**. The existence of sp^3^ C–H supports an early-stage formation of the seven-membered ring, instead of a pathway involving a double [6]helicene intermediate. Due to the limited quantity obtained under these conditions, further purification would have led to significant material loss and was therefore not pursued.


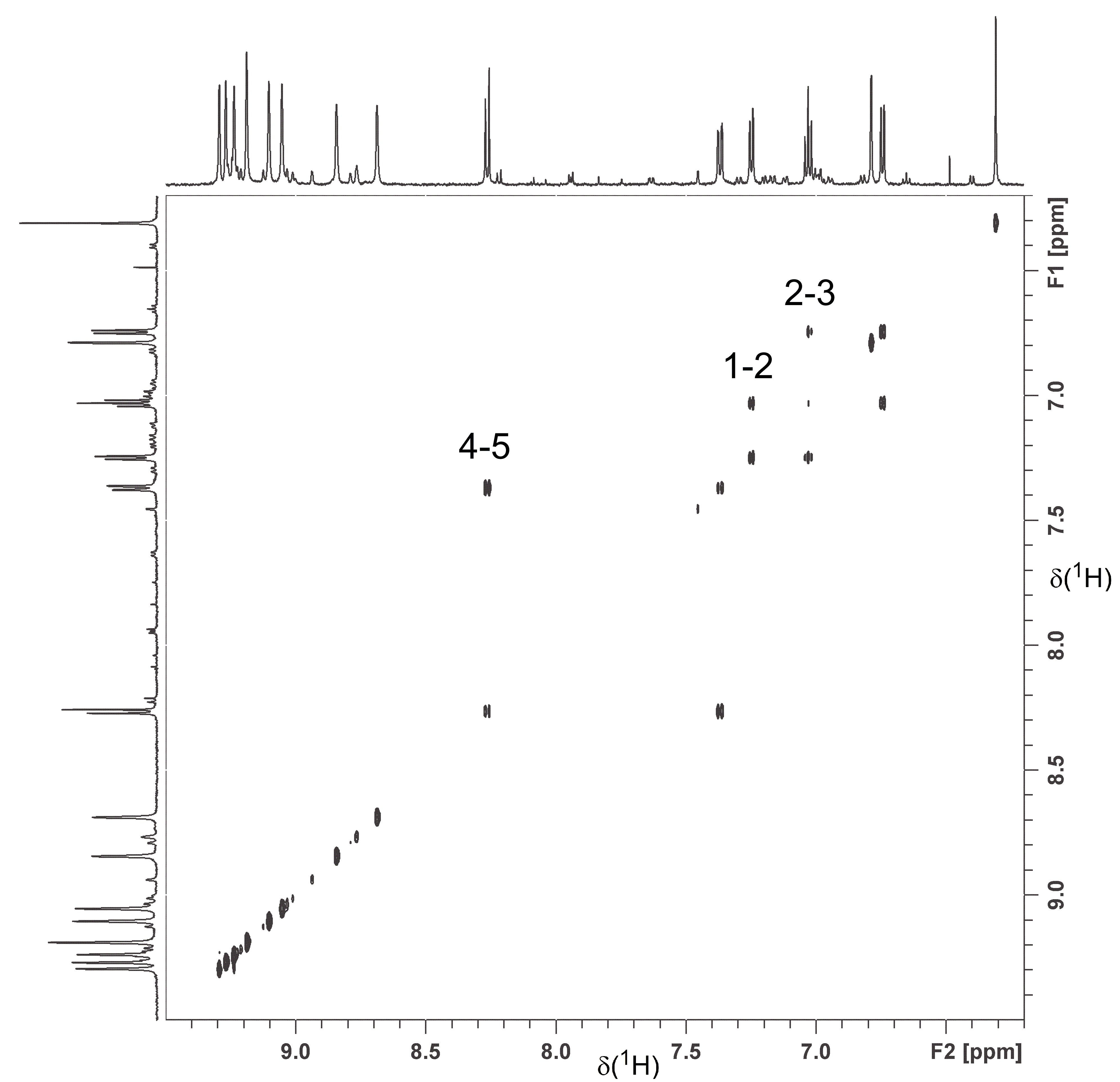


**Figure S48.** ^1^H-^1^H COSY spectrum (region of aromatic protons) of **5** (600 MHz, C_2_D_2_Cl_4_).


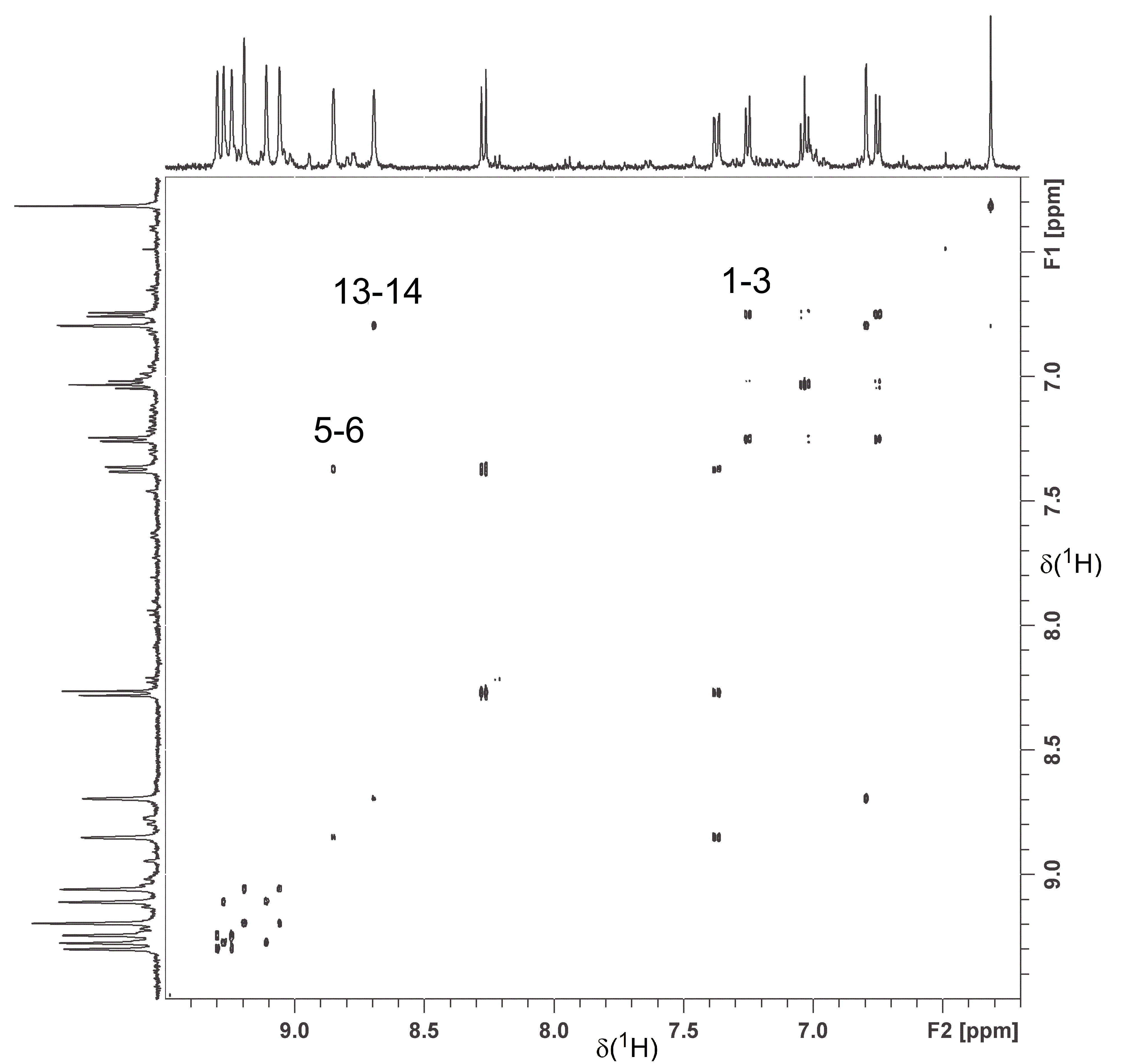


**Figure S49.** Long-range ^1^H-^1^H COSY spectrum (region of aromatic protons) of **5** (500 MHz, C_2_D_2_Cl_4_).


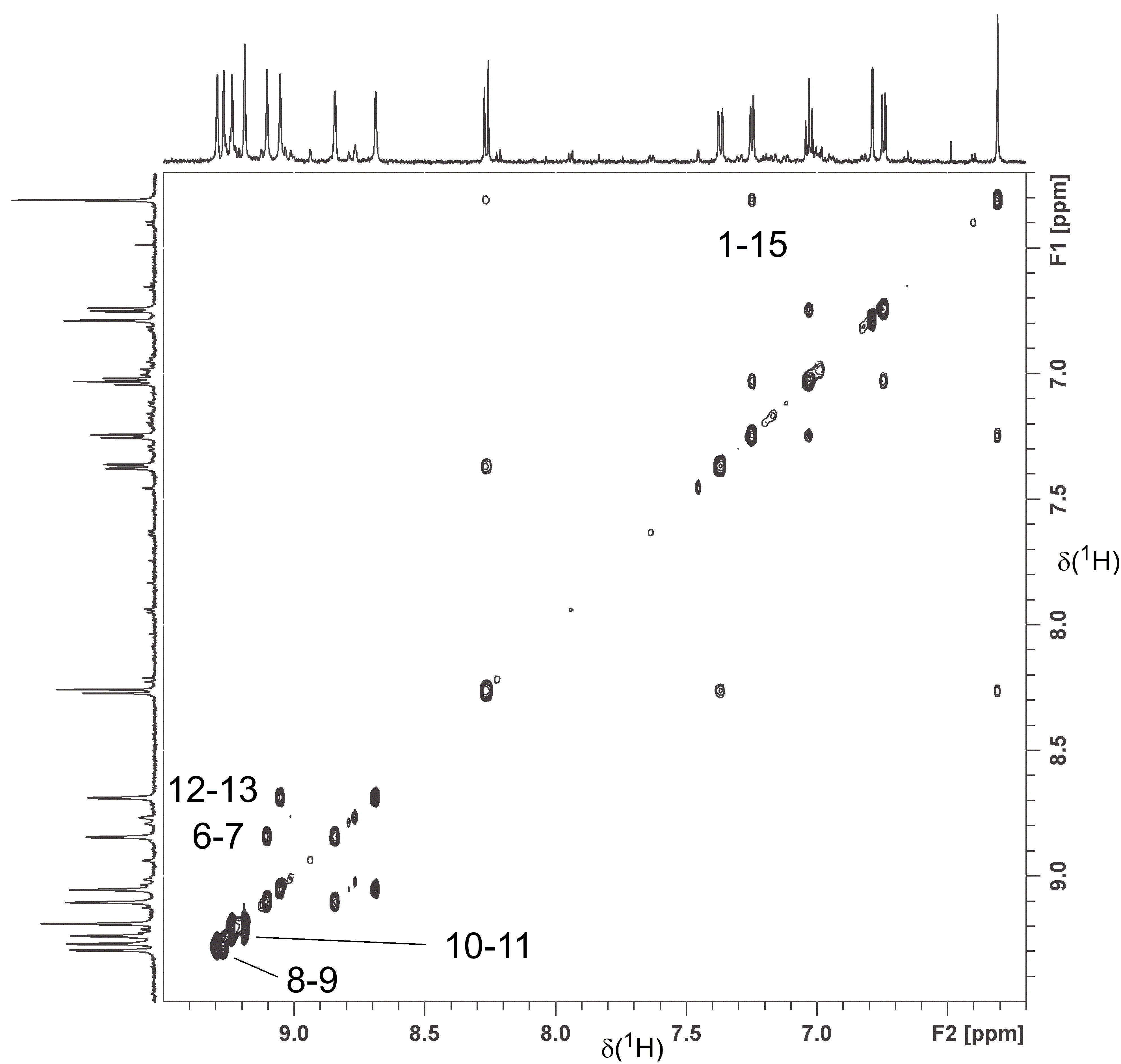


**Figure S50.** ^1^H-^1^H NOESY spectrum (region of aromatic protons) of **5** (600 MHz, C_2_D_2_Cl_4_).


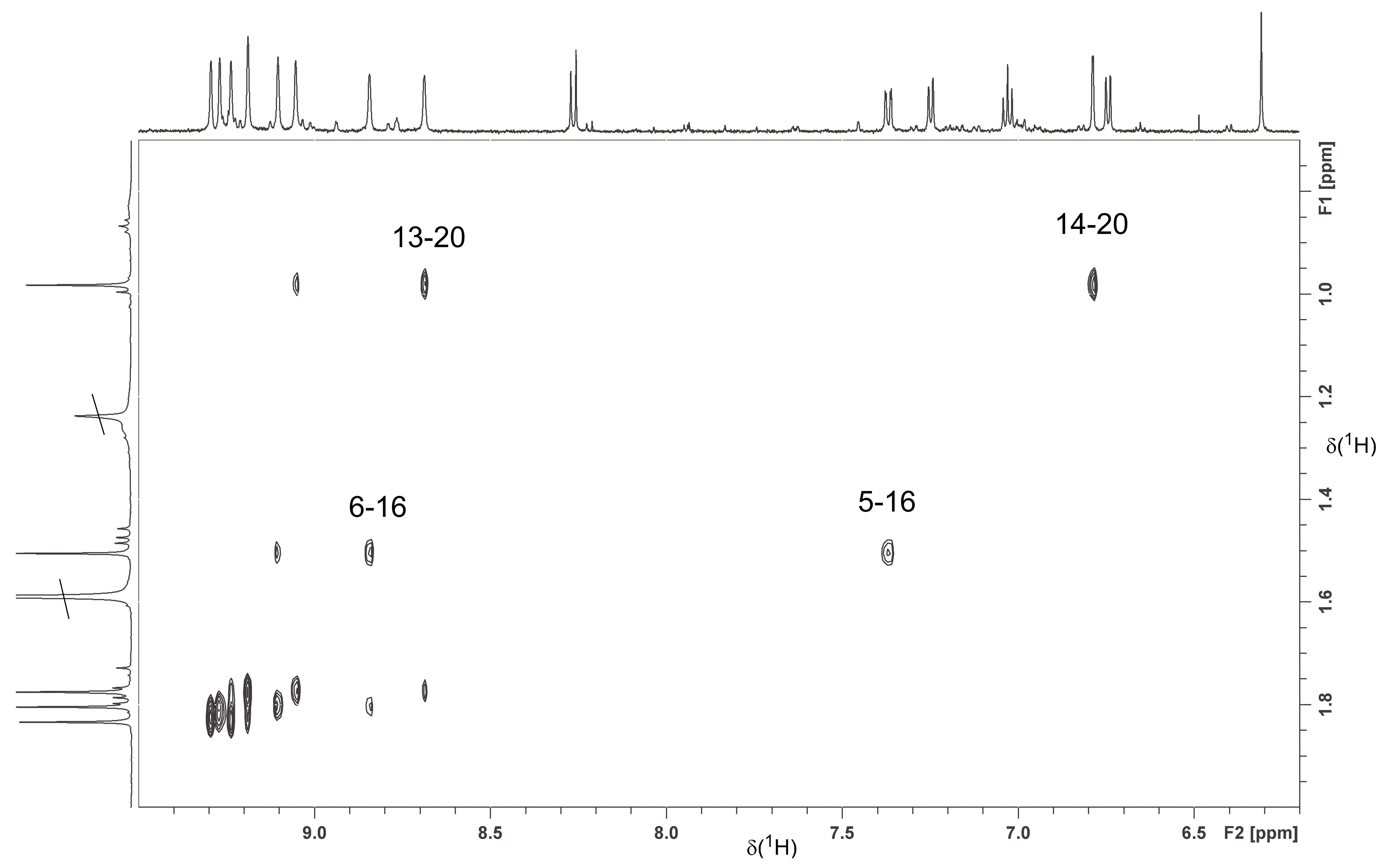


**Figure S51.** ^1^H-^1^H NOESY spectrum (correlations of methyl and aromatic protons) of **5** (600 MHz, C_2_D_2_Cl_4_).


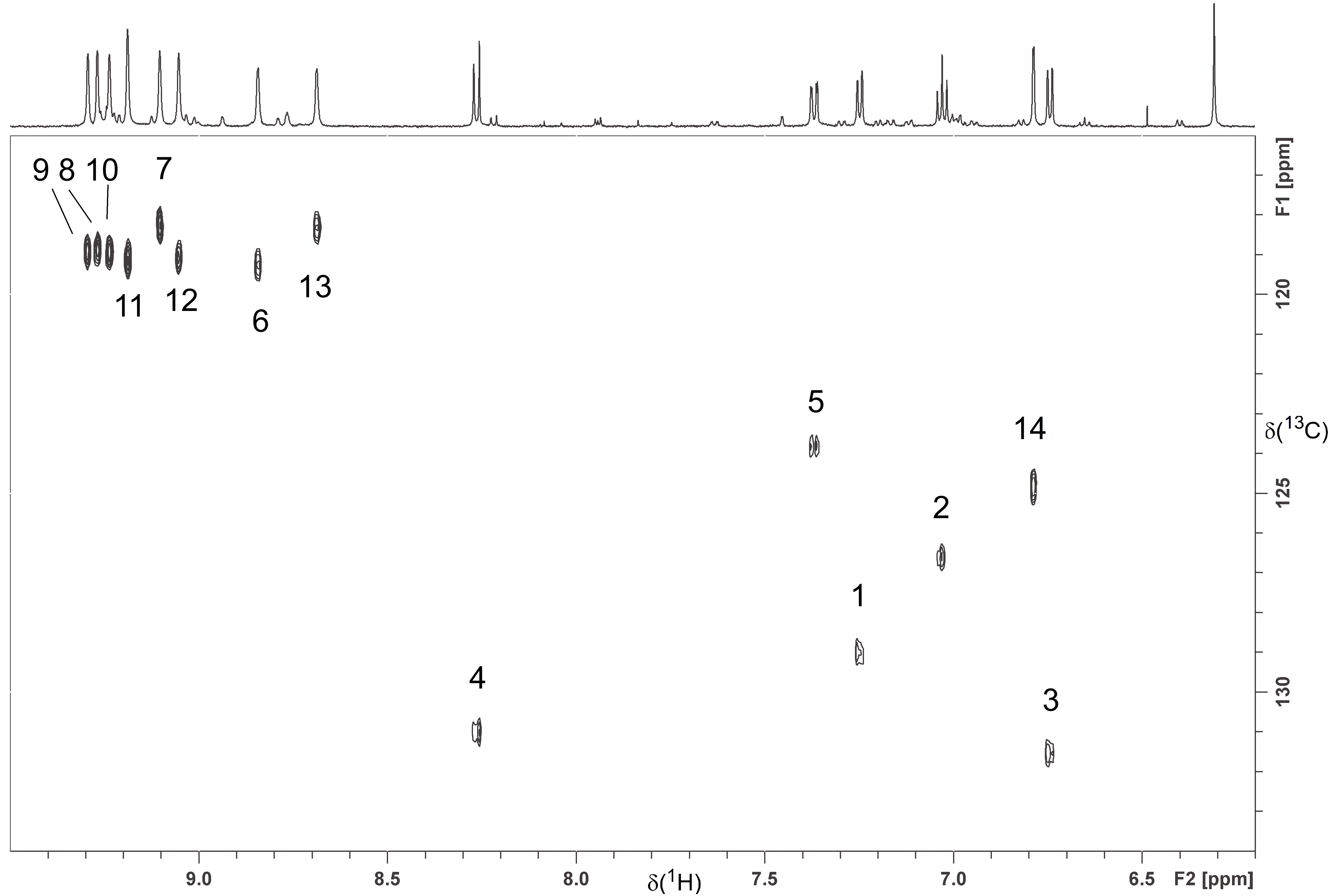


**Figure S52.** ^1^H-^13^C HSQC spectrum (region of aromatic CH carbons) of **5** (600/150 MHz, C_2_D_2_Cl_4_).





**Figure S53.** High-resolution MALDI-TOF MS spectrum of compound **3**.





**Figure S54.** High-resolution MALDI-TOF MS spectrum of compound **4** with dioxygen adduct.





**Figure S55.** High-resolution MALDI-TOF MS spectrum of compound **2**.





**Figure S56.** High-resolution MALDI-TOF MS spectrum of compound **1**.

# **12. Chiral resolution**

Compound **2** was subjected to chiral HPLC in CHCl_3_:*n*-hexane (20:80) on a (*R,R*)-Whelk-O 1 5 μm Kromasil column (25 cm x 21.1 mm). The separation chromatogram is depicted in Figure **S57**.


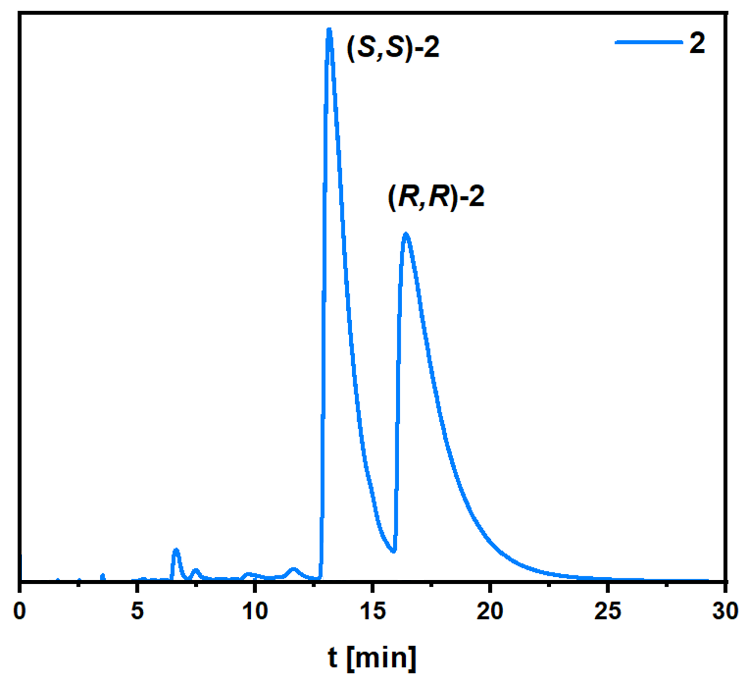


**Figure S57.** Chromatogram of the chiral separation of **2**.

Compound **1** was subjected to chiral HPLC in CHCl_3_:*n*-hexane (20:80) on a (*R,R*)-Whelk-O 1 5 μm Kromasil column (25 cm x 21.1 mm). The separation chromatogram is depicted in Figure **S58**.





**Figure S58.** Chromatogram of the chiral separation of **1**.

**13. References**

[1] CrysAlisPRO, Oxford Diffraction/Agilent Technologies UK Ltd., **2016**.

[2] O. V. Dolomanov, L. J. Bourhis, R. J. Gildea, J. A. K. Howard, H. Puschmann,
“OLEX2: a complete structure solution, refinement and analysis program”, *J. Appl. Crystallogr.* **2009**, *42*, 339–341.

[3] G. M. Sheldrick, “SHELXT – integrated space-group and crystal-structure determination”, *Acta Crystallogr. Sect. A* **2015**, *71*, 3–8.

[4] G. M. Sheldrick, “Crystal structure refinement with SHELXL”, *Acta Crystallogr. Sect. C* **2015**, *71*, 3–8.

[5] C. F. Macrae, I. Sovago, S. J. Cottrell, P. T. A. Galek, P. McCabe, E. Pidcock, M. Platings, G. P. Shields, J. S. Stevens, M. Towler, P. A. Wood, “Mercury 4.0: from visualization to analysis, design and prediction”, *J. Appl. Crystallogr.* **2020**, *53*, 226–235.

[6] A. L. Spek, “Single-crystal structure validation with the program PLATON”, *J. Appl. Crystallogr.* **2003**, *36*, 7–13.

[7] K. Hedberg, L. Hedberg, D. S. Bethune, C. A. Brown, H. C. Dorn, R. D. Johnson, M. de Vries, “Bond lengths in free molecules of buckminsterfullerene, C_60_, from gas-phase electron diffraction”, *Science* **1991**, *254*, 410–412.

[8] H.-B. Bürgi, E. Blanc, D. Schwarzenbach, S. Liu, Y. Lu, M. M. Kappes, J. A. Ibers, “The Structure of C_60_: Orientational Disorder in the Low-Temperature Modification of C_60_”, *Angew. Chem. Int. Ed. Engl.* **1992**, *31*, 640–643.

[9] S. Liu, Y. J. Lu, M. M. Kappes, J. A. Ibers, “The structure of the C_60_ molecule: X-ray crystal structure determination of a twin at 110 K”, *Science* **1991**, *254*, 408–410.

[10] M. J. Frisch, G. W. Trucks, H. B. Schlegel, G. E. Scuseria, M. A. Robb, J. R. Cheeseman, G. Scalmani, V. Barone, G. A. Petersson, H. Nakatsuji, et al., Gaussian 16, Rev. C.01, Gaussian, Inc., Wallingford CT, **2016**.

[11] S. Grimme, S. Ehrlich, L. Goerigk, “Effect of the Damping Function in Dispersion Corrected Density Functional Theory”, *J. Comput. Chem.* **2011**, *32*, 1456–1465.

[12] Avogadro v1.1.1, M. D. Hanwell, D. E. Curtis, D. C. Lonie, T. Vandermeersch, E. Zurek, G. R. Hutchison, “Avogadro: An advanced semantic chemical editor, visualization, and analysis platform”, *J. Cheminf.* **2012**, *4*, 17.

[13] J. Kruszewski, T. M. Krygowski, “Definition of aromaticity basing on harmonic oscillator model”, *Tetrahedron Lett.* **1972**, *13*, 3839–3842.

[14] T. M. Krygowski, “Crystallographic studies of inter- and intramolecular interactions reflected in aromatic character of pi-electron systems”, *J. Chem. Inf. Comput. Sci.* **1993**, *33*, 70–78.

[15] R. Herges, D. Geuenich, “Delocalization of Electrons in Molecules”, *J. Phys. Chem. A* **2001**, *105*, 3214–3220.

[16] P. v. R. Schleyer, C. Maerker, A. Dransfeld, H. Jiao, N. J. R. van Eikema Hommes, “Nucleus-Independent Chemical Shifts: A Simple and Efficient Aromaticity Probe”, *J. Am. Chem. Soc.* **1996**, *118*, 6317–6318.

[17] T. Lu, F. Chen, “Multiwfn: A multifunctional wavefunction analyzer”, *J. Comput. Chem.* **2012**, *33*, 580–592.

[18] T. Lu, “A comprehensive electron wavefunction analysis toolbox for chemists, Multiwfn”, *J. Chem. Phys.* **2024**, *161*, 082503.

[19] S. Klod, E. Kleinpeter, “Ab initio calculation of the anisotropy effect of multiple bonds and the ring current effect of arenes—application in conformational and configurational analysis”, *J. Chem. Soc. Perkin Trans. 2* **2001**, 1893–1898.

[20] X. Wang, Z. Liu, J. Wang, T. Lu, W. Xiong, X. Yan, M. Zhao, M. Orozco-Ic, “Electronic Structure and Aromaticity of an Unusual Cyclocarbon Precursor, C_18_Br_6_”, *Chem. Eur. J.* **2023**, *28*, e202300348.

[21] Z. Liu, T. Lu, S. Hua, Y. Yu, “Aromaticity of Hückel and Möbius Topologies Involved in Conformation Conversion of Macrocyclic [32]Octaphyrin(1.0.1.0.1.0.1.0): Refined Evidence from Multiple Visual Criteria”, *J. Phys. Chem. C* **2019**, *123*, 18593–18607.

[22] Z. Liu, T. Lu, Q. Chen, “An *sp*-hybridized all-carboatomic ring, cyclo[18]carbon: Bonding character, electron delocalization, and aromaticity”, *Carbon* **2020**, *165*, 468–475.

[23] X. Wang, Z. Liu, X. Yan, T. Lu, W. Zheng, W. Xiong, “Bonding Character, Electron Delocalization, and Aromaticity of Cyclo[18]Carbon (C_18_) Precursors, C_18_-(CO)n (n=6, 4, and 2): Focusing on the Effect of Carbonyl (-CO) Groups”, *Chem. Eur. J.* **2022**, *28*, e202103815.

[24] H. Eyring, “The Activated Complex in Chemical Reactions”, *J. Chem. Phys.* **1935**, *3*, 107–115.

[25] S. Glasstone, K. J. Laidler, H. Eyring, “The Theory of Rate Processes: The Kinetics of Chemical Reactions, Viscosity, Diffusion and Electrochemical Phenomena” McGraw-Hill: New York, **1941**.

[26] J. A. Berson, “The Stereochemistry of Sigmatropic Rearrangements. Tests of the Predictive Power of Orbital Symmetry Rules”, *Acc. Chem. Res.* **1968**, *1*, 152–160.

[27] D. G. Truhlar, B. C. Garrett, S. J. Klippenstein, “Current Status of Transition-State Theory” *J. Phys. Chem.* **1996**, *100*, 12771–12800.
